# The Within-Subject Association of Physical Behavior and Affective Well-Being in Everyday Life: A Systematic Literature Review

**DOI:** 10.1007/s40279-024-02016-1

**Published:** 2024-05-06

**Authors:** Irina Timm, Marco Giurgiu, Ulrich Ebner-Priemer, Markus Reichert

**Affiliations:** 1 Mental mHealth Lab, Institute of Sports and Sports Science, Karlsruhe Institute of Technology, Hertzstr. 16, 76187 Karlsruhe, Germany; 2 Department of Psychiatry and Psychotherapy, Central Institute of Mental Health, Medical Faculty Mannheim/Heidelberg University, Mannheim, Germany; 3 Department of eHealth and Sports Analytics, Faculty of Sport Science, Ruhr University Bochum, Gesundheitscampus-Nord 10, 44801 Bochum, Germany; 4 German Center for Mental Health (DZPG), partner site Mannheim, Mannheim, Germany

## Abstract

**Background:**

The interplay of physical activity (PA) with affective well-being (AWB) is highly critical to both health behaviors and health outcomes. Current prominent theories presume AWB to be crucial for PA maintenance, and PA is evidenced to foster mental health. However, thus far, PA-AWB associations have mainly been researched in laboratory settings and with interventional designs, but the everyday life perspective had not been focused on, mostly due to technological limitations. In the course of digitization, the number of studies using device-based methods to research the within-subject association of physical activity and affective well-being (PA-AWB) under ecological valid conditions increased rapidly, but a recent comprehensive systematic review of evidence across populations, age groups, and distinct AWB components remained inconclusive.

**Objectives:**

Therefore, we aimed to firstly review daily-life studies that assessed intensive longitudinal device-based (e.g., electronic smartphone diaries and accelerometry) and real-time PA-AWB data, secondly to develop and apply a quality assessment tool applicable to those studies, and thirdly to discuss findings and draw implications for research and practice.

**Methods:**

To this end, the literature was searched in three databases (Web of Science, PubMed, Scopus) up to November 2022. The systematic review followed the PRISMA guidelines and had been pre-registered (PROSPERO id: CRD42021277327). A modified quality assessment tool was developed to illustrate the risk of bias of included studies.

**Results:**

The review of findings showed that, in general, already short PA bouts in everyday life, which clearly differ from structured exercise sessions, are positively associated with AWB. In particular, feelings of energy relate to incidental (non-exercise and unstructured) activity, and PA-AWB associations depend on population characteristics. The quality assessment revealed overall moderate study quality; however, the methods applied were largely heterogeneous between investigations. Overall, the reviewed evidence on PA-AWB associations in everyday life is ambiguous; for example, no clear patterns of directions and strengths of PA-AWB relationships depending on PA and AWB components (such as intensity, emotions, affect, mood) emerged.

**Conclusions:**

The reviewed evidence can fuel discussions on whether the World Health Organization’s notion “every move counts” may be extended to everyday life AWB. Concurrently, the PA-AWB relationship findings endorse prominent theories highlighting the critical role of AWB in everyday PA engagement and maintenance. However, the review also clearly highlights the need to advance and harmonize methodological approaches for more fine-grained investigations on which specific PA/AWB characteristics, contextual factors, and biological determinants underly PA-AWB associations in everyday life. This will enable the field to tackle pressing challenges such as the issue of causality of PA-AWB associations, which will help to shape and refine existing theories to ultimately predict and improve health behavior, thereby feeding into precision medicine approaches.

**Supplementary Information:**

The online version contains supplementary material available at 10.1007/s40279-024-02016-1.

## Key Points

**Table:** 

The number of daily-life studies using device-based methods (e.g., electronic smartphone diaries and accelerometry) to research the within-subject association of physical activity and affective well-being (PA-AWB) in everyday life has increased rapidly across the last 15 years.
Already short PA bouts in everyday life relate positively to AWB, feelings of energy appear to play a dominant role, and PA-AWB associations depend on population characteristics. However, overall, the reviewed evidence on PA-AWB association characteristics in everyday life is ambiguous.
The quality assessment revealed overall moderate risk of bias; however, methods applied were largely heterogeneous between studies. Therefore, future research in the PA-AWB field should advance and harmonize methodological approaches to overcome challenges in the interpretation of heterogeneous study outcomes.

## Introduction

Physical activity is indispensable for human health, but worldwide and across ages physical activity is declining [1, 2]. Therefore, to foster prevention and treatment of physical and mental disorders, the World Health Organization addresses the prevention of physical inactivity as a major health priority [3, 4]. Towards this aim, a key role is attributed to the within-subject associations of physical activity and human affective well-being in everyday life. This association is critically involved in both physical and mental health processes for motivating, maintaining, and reinforcing physical activity and affective well-being [5–7]. Both immediate emotional responses to physical activity and rational thinking about its benefits are important for initiating and maintaining a physically active lifestyle. The relevance of these associations between physical activity and affective well-being has recently progressed toward dual-process models and hedonism theories [8] for research on behavioral processes. In contrast to traditional health behavior theories that mainly focused on the role of cognitive aspects as physical activity drivers, these recent theories suggest within-subject variance of human well-being in everyday life to be of critical importance for physical activity engagement [9–11]. For example, positive emotional responses that automatically occur as a result of physical activity, along with emotionally driven motivational states, are hypothesized to contribute to the maintenance of an active lifestyle [12, 13]. In this context, these behavioral processes are often described as “micro-temporal within-subject processes,” and they are currently being considered as a highly promising research-path to understand the drivers of regular physical activity engagement [5]. Similarly, the importance of physical activity and affective well-being associations for human mental health appears face valid, for example, with major depression disorder patients exhibiting both diminished mood and psychomotor retardation. Epidemiological studies clearly evidence physical activity to decrease the incidence of several mental disorders in the general population (e.g., [14, 15]). Randomized clinical trials show physical activity to improve treatment outcomes, with most prominent effects in affective disorders [16], and particularly when combined with pharmacotherapy and psychotherapy [17]. While it is still poorly understood how physical activity relates to emotional well-being, recent studies showed that staying physically active can be especially beneficial for people at risk of mental disorders and for those with conditions such as bipolar disorder [18]. Importantly, these benefits may be linked to the way exercise affects specific brain structures associated with mental disorders [19]. In other words, regular physical activity could potentially improve the health of these vulnerable brain areas, reducing the likelihood of experiencing mental disorders.

Within the last decades, laboratory research produced in-depth insights into physical activity and affective well-being associations summarized in several reviews and meta-analyses [20–24], but the everyday life perspective on the physical activity and affective well-being association has not been focused on for some time. Part of this neglect may lie in difficulties of capturing physical activity and affective well-being in the everyday life of humans (e.g., data captured during daily activities such as shopping, gardening, or commuting).

In recent years, this obstacle has been overcome through a group of methods often referred to as ambulatory assessment (AA) [25, 26]. This capitalizes on device-based physical activity measurement via accelerometers and self-reports via electronic diaries (e-diaries) on smartphones for affective well-being assessment [25, 26]. It allows us to capture multiple assessments within a person over time [27], to track data near real-time with increasing ecological validity of data yet reducing retrospective biases [28–30]. A major strength of AA lies in the focus on within-subject variance through the use of intensive longitudinal methods drawing from multiple assessments within persons [31].

In these studies, physical activity (PA) and sedentary behavior (SB) form the superordinate category physical behavior (PB). Energy expenditure-increasing activities performed by any skeletal muscle effort are called PA [32]. In contrast, activities at an energy expenditure < 1.5 metabolic equivalents while remaining in a sitting or reclined position but not during sleep are mainly considered SB [33]. By definition, affective well-being is a subcomponent of human subjective well-being [34], for example, characterized by trait versus state components and domain-specific versus general valuations. The umbrella term *affective well-being* includes core affect, a measure describing a neurophysiological state of an elementary simple primitive affective feeling represented in the circumplex model [35, 36]. Existing studies applied different questionnaires with established psychometric properties to quantify different components of affective well-being, such as the two-dimensional Positive And Negative Affect Scale (PANAS) [37] and the three-dimensional Multi Dimensional Mood Questionnaire [38]. Extensive discussions and empirical analyses on the advantages and limitations of different PA, SB, and affective well-being quantifications can be found elsewhere (see, e.g., [11, 34, 39, 40]). In this review, we refer to the intricate, two-way relationships between physical behavior (which includes physical activity and sedentary behavior) and indicators of affective well-being (measures of emotional health and mood) as “physical behavior–affective well-being (PB-AWB) associations”.

In the past decade, applying AA to research the PB-AWB association has gained tremendous interest as evidenced by the increasing number of studies on PB-AWB associations in recent years (see Electronic Supplementary Material (ESM) 1). While this increase in knowledge can, in principle, provide valuable insights into the understanding of within-subject associations of PB and psychological antecedents and consequences in natural settings, a recent comprehensive systematic review of evidence across populations, age groups, and distinct AWB components is not available thus far. There are two prior works that reviewed the PB-AWB relationship in daily life: the narrative review across a total of 14 studies conducted by Liao and colleagues [41], and the very recent systematic review across ten studies by Bourke and colleagues [42]. Against the background of these prior works, the present review across 66 studies significantly extends the state of knowledge by including studies published after 2015 (resulting in an additional 60 studies compared to the review conducted by Liao et al. [41]); comprehensively covering the relationship between PB, valence, energetic arousal, calmness, energy, and fatigue as AWB components (prior work by Liao and colleagues focused on positive and negative affect [41]); comprising studies in all available populations and across age groups (thereby extending the focus on children and adolescents in Bourke et al.’s work [42]); including studies using state-of-the-art AA methods (e.g., device-based PB assessments; prior work by Liao and colleagues included studies using retrospective PB assessments [41]); and finally, offering a very comprehensive and detailed analysis and providing an in-depth exploration of PB-AWB effects in everyday life.

To this end, we summarize findings of studies that collected data continuously and repeatedly within persons and in real life (so-called “intensive longitudinal methods” [29, 43]). We also developed a modified quality assessment (QA) tool to be used against the background of the large heterogeneity of methods applied in the recent field of AA research on PB-AWB associations following established guidelines for QA tools [44, 45]. Finally, we discuss the findings and draw implications for future real-life studies on PB-AWB associations.

## Methods

This review followed established procedures (PRISMA checklist [46]; for details, see ESM 2) and was registered (PROSPERO id: CRD42021277327).

### Literature Search Strategy

The electronic databases Web of Science, PubMed, and Scopus were systematically searched by selecting the fields’ title and/or abstract and keywords. The terms “ecological momentary assessment,” “mood,” “physical activity,” and “sedentary behavior” as well as their synonyms were searched as follows: “physical activity” or “exercise” or “sedentary behavior” or “sedentariness” or “physical inactivity” plus “mood” or “emotion” or “affect” or “affective states” or “valence” or “calmness” or “energetic arousal” plus “ambulatory assessment” or “ecological momentary assessment” or “experience sampling method” or “electronic sampling method” or “ambulatory monitoring” or “accelerometry” or “physical activity monitoring” or “interactive assessment” or “e-diary” or “electronic diary.” We applied the same search strategy for all three databases, and therefore Boolean operators were adapted to the specific requirements (see ESM 3 for the comprehensive search terms). The last search was conducted in November 2022. We also searched the reference lists of all eligible studies (backward search) to identify further studies.

### Study Eligibility

Studies applying intensive longitudinal device-based and real-time assessments to investigate PB-AWB associations were eligible for this review, and, in particular, articles were included if: (a) PB was captured via device-based measurements (e.g., with accelerometers), the rationale for this being to capture features as objectively as possible, i.e., without (retrospective) distortions from cognitive heuristics [28] (for detailed advantages and disadvantages of device-based versus self-reported PB methods, see [47, 48]); and (b) affective states were self-reported and assessed using an electronic device (e.g., via e-diaries), the rationale for this being that repeated real-life self-reports on electronic devices are the state-of-the-art procedure for a most reliable and ecologically valid assessment of psychological state, e.g., bypassing limitations of traditional paper–pencil diaries [28]; (c) the assessment duration, i.e., the number of days over which the study period extended, was equal to or greater than 1 day (24 h), the rationale for this being to enhance reliability of PB-AWB effects determined and minimize confounding, for example, through well-known diurnal patterns of AWB [49]; (d) momentary (short-term) relationships of PB and AWB had been analyzed (i.e., the aggregated time frames must not extend beyond 24 h; e.g., this criteria includes a study using PB within the last hour of an e-diary prompt as a predictor of AWB, but excludes a study using PB across the evening as a predictor of next-day AWB), the rationale for this being that against the background of well-known recall bias effects [28], we focused on studies investigating PB-AWB associations within 24 h: of note, we did not specify a minimum number of e-diary prompts per day; and (e) people with and without diseases of all ages were included, the rationale being that we aimed to provide a comprehensive review of PB-AWB associations across age groups and populations. Studies were excluded if: (a) PA or SB was captured in controlled (artificial) conditions (e.g., laboratory or research setting or interventions); (b) retrospective questionnaires (e.g., retrospective paper–pencil questionnaires on PB or AWB) were used, and (c) measurements had been taken at a single point in time only (e.g., for cross-sectional PB-AWB analyses). The search was limited to articles published in the English language but conducted independently of the year of publication of the papers. We excluded grey literature (e.g., unpublished manuscripts or dissertation studies) within our PROSPERO registration to ensure consistency in reporting and quality standards; peer-review ensures high quality standards, but including grey literature, where quality standards are not uniformly assessed, could introduce bias into the interpretation of results when mixing peer-reviewed with non-peer-reviewed studies [50, 51].

### Study Selection

**Fig. 1:**
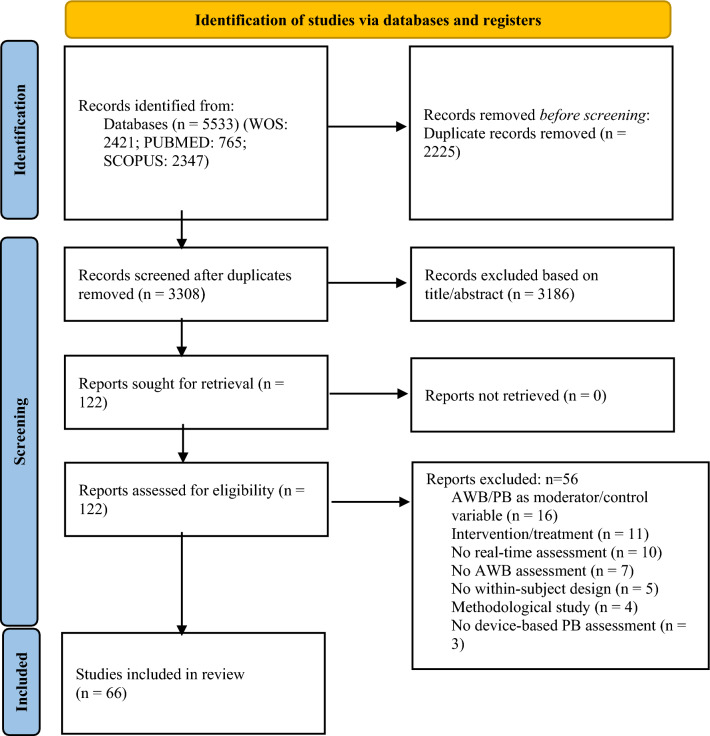
PRISMA flow diagram of the systematic search process [46]. *AWB* affective well-being, *PB* physical behavior

First, study selection was based upon the title initially screened. Second, the title and abstract of potentially eligible studies were screened independently by two researchers (MG, IT). Of the remaining relevant articles, the full text was read to assess potential eligibility. In cases of non-agreement between the two researchers (IT, MG), a third reviewer (MR) was involved to reach a final decision on study inclusion. The selection process is depicted in Fig. 1.

### Data Extraction

A data extraction template was developed to extract data from each study systematically (see Table 1). The data extraction was custom-developed to capture all relevant characteristics of the studies included, applying the following categories: study, country, sample size, sex, age, detailed participant characteristics, ABW assessment, PB assessment, assessment duration, sampling design. To indicate the total time frame of the AA study conduct, we use the term “assessment duration”. In particular, this defines the total time in which participants wore accelerometers and repeatedly answered e-diary prompts in their everyday life. For example, in several studies reviewed, the “assessment duration” covered a 1-week period. To indicate the time frame of PA aggregation for the statistical analysis, we adhered to the term “aggregated time frame”. In particular, this describes the time frame used for parameterization of PA, which does not automatically reflect a continuous bout of the same activity but rather an aggregation of all activities from being sedentary to highly PA. For example, in several studies reviewed, “aggregated time frame” equaled 15 min before and/or after the e-diary prompts. Accordingly, in these studies, researchers investigated associations of PA occurring 15 min before and/or after the e-diary rating with AWB. Details from each study included in the systematic review were extracted by two authors (IT, MG) independently. Thereafter, the two data extraction files were merged. Any discrepancies were discussed among the authors until consensus was reached, and in cases of non-agreement between the two researchers (IT, MG) the vote of a third reviewer (MR) was considered to reach a decision.

**Table 1:** Data extraction of the studies included in the review

Study	Country	*n*	Female %	Age, y (mean, range)	Participants characteristics sample; (study name)	Affect assessment AWB items (software, device); prompts/day	Physical behavior assessment (unit; time frame; direction; device; placement)	Assessment duration; sampling design (random, fixed, event-based, mixed)	Quality assessment
Bai et al. [189]	US	805	71.3	NR (18–25)	Adults (students)	Mood (WE study app, iPhone); 1 prompt/day	PA (metric); 1440 min; before; Apple Watch Series 0 or 1; wrist	50–216 days, fixed	8.5
Bossmann et al. [155]	GER	62	14.5	21.4 (19–30)	Adults (students)	Valence, energetic arousal, calmness (MyExperience, study smartphone); Every hour after waking up	PA (metric); 10 min; before; Movisens Move 1; chest	1 day, fixed	10
Bourke et al. [190]	AU	119	46.4	14.7 (NR)	Adolescents	Valence, energetic arousal, calmness (Qualtrics, participants’ smartphone); 5 (weekdays) or 9 (weekend days) prompts/day	PA (MVPA); 15 min; before; ActiGraph GT3X + ; dominant wrist	4 days, NR	11
Bourke et al. [191]	AU	119	46.4	14.7 (13–17)	Adolescents	Valence, energetic arousal, tense arousal (Qualtrics, participants’ smartphone); 5 (weekdays) or 9 (weekend days) prompts/day	PA (MVPA); 15 min; before; ActiGraph GT3X + ; dominant wrist	4 days, fixed with random component	14
Cabrita et al. [135]	NL	10	60	68.7 (65–83)	Elders	Pleasure (Activity Coach, study smartphone); 12 prompts/day	PA (metric); 10 min; before (accelerometer (NR); hip)	30 days, fixed	8.5
Curtiss et al. [192]	US	34	73.53	28.97 (18–55)	Adults (MDD, anxiety)	Positive affect, negative affect (Ethica App, participants’ smartphone); 5 prompts/day	PA (NR); 0 min; NR; NR; NR	14 days, NR	4.5
Cushing et al. [129]	US	26	42.3	15.96 (13–18)	Adolescents	Positive affect, negative affect, fatigue, energy (PETE app, study smartphone); 4 prompts/day	PA (MVPA) and SB; 30 min; bidirectional; ActiGraph wActi Sleep-BT; nondominant wrist	20 days, fixed	11
Cushing et al. [81]	US	26	42.3	15.67 (13–18)	Adolescents	Anger, anxiety, depression (PETE app, study smartphone); 4 prompts/day	PA (MVPA); 30 min; bidirectional; ActiGraph wActi Sleep-BT; nondominant wrist	20 days, fixed	9.5
DeMasi et al. [193]	US	53	49	19.83 (NR)	Adults (students)	Mood, energy (Open Sensing Framework App, participants’ smartphone); 4 prompts/day	PA (metric); 1440 min; before; smartphone’s accelerometer; no fixed position	56 days, random	9
Difrancesco et al. [194]	NL	359	63.7	49.5 (NR)	Adults (MDD, anxiety)	Positive affect, negative affect (NR, participants’ or study smartphone); 5 prompts/day	PA (LPA, MVPA) and SB; 180 min; bidirectional; GENEActive; nondominant wrist	14 days, fixed	7
Dunton et al. [162]	US	119	52	NR (9–13)	Children (Healthy PLACES)	Positive affect, negative affect (MyExperience, study smartphone); 3–7 prompts/day	PA (MVPA); 30 min; bidirectional; ActiGraph GT2M; right hip	8 days (2 waves × 4 days), fixed with random component	10
Elavsky et al. [195]	US	121	100	51.5 (40–60)	Adults	Positive affect, negative affect (Purdue Momentary Assessment Tool, PDA); 4 prompts/day	SB; 180–360 min; bidirectional; ActiGraph GT1M; nondominant hip	15 days, mixed	10
Giurgiu et al. [90]	AU; GER	92	63	33.7 (22–62)	Adults (university employee)	Valence, energetic arousal, calmness (movisensXS, study smartphone); 8–21 prompts/day	PA (metric) and SB; 15–30 min; before; Movisens Move 3; chest, hip, thigh	5 days, mixed	13
Giurgiu et al. [86]	AU; GER	92	65	33.73 (22–62)	Adults (university employee)	Valence, energetic arousal, calmness (movisensXS, study smartphone); 8–21 prompts/day	PA (metric); 80 min; before; Movisens Move 3 + EcgMove 3; chest, hip, thigh	5 days, mixed	12.5
Giurgiu et al. [94]	AU; GER	92	65	33.88 (22–62)	Adults (university employee)	Valence, energetic arousal, calmness (movisensXS, study smartphone); 8–21 prompts/day	SB; 30 min; after; Movisens Move 3 + EcgMove 3; chest, hip, thigh	5 days, mixed	14
Giurgiu et al. [89]	GER	103	55.1	22.1 (19.3–24.9)	Adults (students)	Valence, energetic arousal, calmness (movisensXS, study smartphone); 6 prompts/day	PA (LPA, MVPA) and SB; 60 min; Movisens Move 4; wrist, hip, thigh	5 days, mixed	12.5
Haaren et al. [156]	GER	29	NR	21.3 (NR)	Adults (students)	Valence, energetic arousal, calmness (My Experience, PDA); 5 prompts/day	PA (metric, LPA) and SB; 15/-30 min; before; Movisens Move 2; chest	2 days, fixed with random component	11.5
Hevel et al. [196]	US	103	62.5	72.4 (60–98)	Adults	Positive affect, negative affect, energy (movisensXS, study smartphone); 6 prompts/day	PA (metric); 15/-30 min; bidirectional; ActivePAL; thigh	10 days, fixed with random component	10.5
Jeckel and Sudeck [112]	GER	46	54.4	32 (21–59)	Adults	Valence, energetic arousal, calmness (MyExperience, study smartphone); 4 prompts/day and before and after activity	PA (metric); 15/-720 min; before; Movisens EcgMove; chest	6 days, mixed	12
Jeckel and Sudeck [197]	GER	46	54.4	32 (21–59)	Adults	Valence, energetic arousal, calmness (MyExperience, study smartphone); Activity-triggered prompts	PA (metric); 15 min; bidirectional; Movisens EcgMove; chest	6 days and 15 h, event	11
Kanning et al. [198]	GER	44	47.7	26.2 (NR)	Adults (students)	Valence, energetic arousal, calmness (Izybuilder, PDA); 19 prompts/day	PA (metric); 10 min; before; Becker Meditech Varioport-e; hip	1 day, fixed with random component	9.5
Kanning [199]	GER	87	54	24.6 (NR)	Adults (students)	Valence, energetic arousal, calmness (Izybuilder, PDA); 19 prompts/day	PA (metric); 10 min; before; Becker Meditech Varioport-e; hip	1 day, fixed with random component	10.5
Kanning et al. [154]	GER	74	49	60.1 (50–70)	Adults	Valence, energetic arousal, calmness (MyExperience, study smartphone); Activity-triggered prompts only (mean 6.4)	PA (metric); 10 min; before; Becker Meditech Varioport-e; hip	3 days, mixed	11
Kanning and Schoebi [87]	GER	65	57	24.6 (NR)	Adults (students)	Valence, energetic arousal, calmness (Izybuilder, PDA); Every 45 min during pre-defined 14- h period)	PA (metric); 5-/45 min; after; Becker Meditech Varioport-e; hip	1 day, fixed	10.5
Kanning et al. [173]	GER; US	202	100	41 (24–57)	Adults (mothers of 8 to 12-year-old children)	Happy, calm, stressed, angry, sad/depressed (NR, study smartphone); 4 (weekdays) or 8 (weekend days) prompts/day	PA (MVPA); 120 min; before; ActiGraph GT3X; hip	8 days, fixed with random component	9.5
Kanning et al. [200]	GER	308	50.3	27.4 (17–66)	Adults (students and employees)	Valence, calmness, energetic arousal (movisensXS, study smartphone); Sedentary-triggered prompts	SB; 30 min; before; Movisens Move 3 or Move 4; thigh	4–5 days, mixed	11.5
Kim et al. [125]	JP	113	28.3	13.6 (NR) undergraduates: 21.6 (NR) office workers: 41.0 (NR)	Adolescents, adults (undergraduates and office workers)	Depressive mood (NR, wristwatch); 5 (adolescents), 6 (office workers) or 10 (undergraduates) prompts/day	PA (metric); 60 min; before; wristwatch computer (Ruputer); nondominant wrist	2–7 days, mixed	11.5
Kim et al. [126]	JP	57	EG: 14.3 HC: 0	EG: 34 (22–42) HC: 40.7 (23–58)	Adults (with and without MDD)	Depressive mood (NR, wristwatch); 6 prompts/day	PA (metric); 60 min; before; wristwatch computer (Ruputer); nondominant wrist	EG: 18–67 days; HC: 7 days, mixed	10.5
Kim et al. [175]	JP; US	122	76.4	41.3 (19–63)	Adults	Valence, energetic arousal (NR, PDA); 6 prompts/day	PA (metric) and SB; 5-/60-/120 min; bidirectional; ActiHeart; chest	3 days, fixed with random component	9.5
Koch et al. [93]	GER	113	48	15.02 (12–17)	Adolescents (URGENY)	Valence, energetic arousal, calmness (MovisensXS, study smartphone); 4–7 (weekdays) or 8–17 (weekend days) prompts/day	PA (metric), 10 min; after; Movisens Move 2 + Move 3; right hip	7 days, mixed	13.5
Koch et al. [91]	GER	113	48	15.02 (12–17)	Adolescents (URGENY)	Valence, energetic arousal, calmness (MovisensXS, study smartphone); 4–7 (weekdays) or 8–17 (weekend days) prompts/day	PA (metric), 15 min; before; Movisens Move 2 + Move 3; right hip	7 days, mixed	12
Koch et al. [80]	GER; ESP; GB; NL	185	54.1	(14–45)	Adolescents, adults (with and without ADHD)	Positive and negative affect (movisens XS, NR); 12 prompts/day	PA (metric); 10 min; before; Movisens LightMove 3; non-dominant wrist	4 days, random	7.5
Kracht et al. [201]	US	284	54	12.6 (10–16)	Adolescents (TIGER Kids study)	Positive affect (LifeData Corporation App, study device) or participants’ smartphone); 2 (weekdays) or 6 (weekend days) prompts/day	SB; 30 min; before; ActiGraph GT3X + ; hip	7 days, fixed with a random component	13.5
Kuehnhausen et al. [202]	GER	82	45	117.2 (97–132) months	Children (FLUX)	Pleasantness, unpleasantness, activation, deactivation (NR, study smartphone); 4 prompts/day	PA (MVPA); 1440 min; before; ActiGraph GT3X + ; hip	28 days, NR	6.5
Langguth et al. [127]	GER	72	37	17.36 (12–26)	Adolescents	Depression (NR, paper–pencil or online diaries); 3 prompts/day	PA (MVPA); 1440 min; before; ActiGraph GT3X + ; hip	7 days, mixed	11.5
Le et al. [203]	AU	361	72.5	22.79 (NR)	Adults	Positive affect, negative affect, affective arousal (MetricWire, NR); 3–4 prompts/day	PA (LPA, MVPA) and SB; 1140 min; NR; ActiGraph wGT3X-BT; wrist	7–15 days, fixed with a random component	7.5
Li et al. [204]	UK	78	71.79	25.46 (NR)	Adults	Positive affect, negative affect, depression (movisensXS or Qumi, participants’ smartphone); 5 prompts/day	PA (LPA, MVPA) and SB; 0–180 min; Movisens EcgMove 3; chest	14 days, fixed with a random component	10
Liao et al. [205]	US	117	72.5	40.4 (NR)	Adults (MOBILE)	Positive affect, negative affect, energy, fatigue (MyExperience, study smartphone); 8 prompts/day	PA (MVPA, LPA); 15-/30 min; bidirectional; ActiGraph GT2 M; hip	4 days, fixed with random component	13.5
Liao et al. [206]	US	117	73	39.8 (NR)	Adults (MOBILE)	Positive affect, negative affect, energy, fatigue (MyExperience, study smartphone); 8 prompts/day	PA (MVPA); 6–12 months; after; ActiGraph GT2 M; NR	4 days × 3 waves, random	6
Madden et al. [95]	US	21	76.2	49 (NR)	Adults (MDD, bipolar, schizophrenia)	Positive affect, negative affect, energy, fatigue (custom software phone application, study smartphone); 7 prompts/day	PA (MVPA); 30 min; bidirectional; ActiGraph wGT3x-BT; right hip	4 days, fixed with random component	12
McLean et al. [207]	US	75	63	31 (NR)	Adults	Valence (Personal Analytics Companion, participants’ smartphone); 6 prompts/day	PA (metric); 60 min; before; Fitbit Flex; wrist	7 days, fixed with random component	9.5
Merikangas et al. [18]	US	242	61.9	48 (NR)	Adults (MDD, bipolar) (NIMH)	Mood, energy (NR, PDA); 4 prompts/day; 4 prompts/day	PA (metric); 240 min; bidirectional; Respironics and Actiwatch; nondominant wrist	14 days, fixed	6
Michalak et al. [208]	GER	71	60.6	39.33 (NR)	Adults (MDD)	Mood and depressive symptoms (Palm Tungsten T3, NR); 14 prompts/day	PA (metric); 60 min; before; Vitamove; trunk, right thigh	2 days, fixed	8.5
Pannicke et al. [209]	AT	37	75.7	23.5 (19–28)	Adults	Positive affect, negative affect (NR, participants’ smartphone); 6 prompts/day	PA (MVPA) and SB; 150 min; before; Actiheart; sternum, chest	7 days, fixed	11.5
Pinto et al. [210]	US	22	100	51.5 (NR)	Adults (breast cancer survivors)	Affective valence, sadness, anxiety, stress, fatigue (mEMA ilumivu, participants’ smartphone); 5 prompts/day	SB; 1440 min; after; ActiGraph GT3X; right hip	7 days × 5 waves, mixed	9.5
Poppe et al. [211]	BE	38	34.2	63.18 (50–81)	Adults (with type 2 diabetes mellitus)	Stress, sadness, fatigue (LimeSurvey, participants’ computer or tablet); 1 prompt/day	PA (LPA, MVPA) and SB; 1440 min; after; ActiGraph GT3X + ; right hip	10 days, event	11.5
Powell et al. [212]	UK	29	36	71.4 (46–85)	Adults (after joint replacement surgery)	Positive affect, negative affect (NR, PDA); median of 6 prompts/day	PA (metric); 60-/1440 min; bidirectional; Vitaport 3 system; thigh, chest	1 day, fixed with random component	11.5
Reichert et al. [92]	GER	106	62.4	23.4 (18–27)	Adults (URGENCY)	Valence, energetic arousal, calmness (movisensXS, study smartphone); 9–22 prompts/day	PA (metric); 10 min; after; Movisens Move 2; hip	7 days, mixed	13
Reichert et al. [88]	GER	106	62.4	23.4 (18–27)	Adults	Valence, energetic arousal, calmness (movisensXS, study smartphone); 9–22 prompts/day	PA (metric); 15/1440 min; before; Movisens Move 2; hip	7 days, mixed	11.5
Ruissen et al. [85]	CA	126	48.4	27.71 (18–40)	Adults	Positive affect, negative affect (MetricWire, participants’ smartphone); 6 prompts/day	PA (MVPA); n.a.^a^; bidirectional; Fitbit Blaze; wrist	14 days, fixed with random component	10.5
Schwerdtfeger et al. [83]	GER	124	51.6	31.67 (18–73)	Adults	Positive affect, negative affect (DialogPad, PDA); 13 prompts/day	PA (metric, MVPA, LPA) and SB; 1–30 min; after; ActiGraph GT1M; ankle	12 h, fixed with random component	13.5
Shin et al. [213]	KR	27	29.6	NR (19–44)	Adults	Joyful, good, nervous, tired, depressed, annoyed, upset (Google Forms, participants’ smartphone); 2 prompts/day	PA (metrics); 1440 min; after; Fitbit; NR	5 days, fixed	6.5
Smith et al. [131]	US	17	58.8	10.59 (NR)	Children (with overweight/obesity)	Negative affect, positive affect (ReTAINE, smartphone NR); 4–6 prompts/day and after meals	PA (LPA, MVPA) and SB; 30-/60-/120 min; bidirectional; Philips Actiwatch 2; wrist	14 days, mixed	9.5
Smith et al. [214]	US	77	41.6	15.36 (13–17)	Adolescents (with and without overweight)	Negative affect, positive affect (NR, study smartphone); 4 (weekdays) or 7 (weekend days) prompts/day	PA (metric, MVPA); 60 min; bidirectional; ActiGraph (NR); waist	7 days; fixed	10
Stavrakakis et al. [157]	NL	20	70 (each group)	depressed: 36.4 (22–49) nondepressed: 36.7 (24–46)	Adults (with and without MDD) (MOOVD)	Negative affect, positive affect (PsyMate, NR); 3 prompts/day	PA (metric); 360 min; bidirectional; Respironics ActiCal; nondominant wrist	30 days, fixed	10
Stevenson et al. [82]	(NR)	25	56	40 (NR)	Adults (alcohol use disorder)	Positive affect, negative affect (ilumivu, participants’ smartphone); 4 prompts/day	PA (metric); 60-/1440 min; before; Fitbit Charge; wrist	21 days, fixed with random component	10.5
Sudeck et al. [215]	GER	64	58.3	35.18 (20–63)	Adults	Valence, energetic arousal, calmness (movisensXS, study smartphone); 4 prompts/day	PA (metric); 15 min; before; Movisens Move 3; hip	4 days, fixed with random component	11.5
Takano et al. [216]	JP	41	22	NR	Adults (undergraduate students)	Positive affect, negative affect (NR, participants’ smartphone); 8 prompts/day	PA (metric); 15 min; before; Respironics Actiwatch; wrist	7 days, fixed with random component	11
Vetrovsky et al. [217]	CZ	28	75	68 (NR)	Adults	Fatigue (NR, participants’ smartphone); 1 prompt/day	PA (metric, MVPA); 720 min; after; ActiGraph wGT3X-BT; right hip	28 days, fixed	11.5
Walsh et al. [218]	US	111	60.36	22.01 (18–27)	Adults (bipolar)	Depression (NR, NR); 3 prompts/day	PA(MVPA) and SB; 1440 min; before; Philips Actiwatch Spectrum; nondominant wrist	20 days, fixed with random component	7
Wen et al. [132]	US	202	51.67	9.6 (8–12)	Children (MATCH)	Positive affect, negative affect (NR, study or participants’ smartphone); 3–7 prompts/day	PA (MVPA) and SB; 30-/, 60 min; bidirectional; ActiGraph GT3X; right hip	7 days, random	11.5
Wilhelm et al. [136]	CA	123	63	71.83 (64–85)	Elders	Negative affect (NR, paper–pencil); 3 prompts/day	PA (metric); 1440 min after; ActiGraph GT3X; hip	10 days, fixed	10
Williams et al. [219]	US	194	71	40.72 (20–74)	Adults	Positive affect, negative affect (online survey, NR); 1 prompt/day	PA (metric); 1440 min; after; Fitbit Charge; NR	100 days, fixed	7
Yang et al. [79]	US	185	Mothers: 100; Children: 53	Mothers: 41.03 (NR); children: 9.51 (NR)	Children, adults (MATCH)	Positive affect, negative affect (NR, participants’ or study smartphone); mothers: 4 (weekdays) or 8 (weekend days) prompts/day; children: 3 (weekdays) or 7 (weekend days) prompts/day	PA (MVPA) and SB; 45 min; bidirectional; ActiGraph GT3X or WGT3X-BT; hip	7 days, fixed with random component	11.5
Zenk et al. [78]	US	128	100	NR (25–64)	Adults	Positive affect, negative affect (NR, study smartphone); 5 prompts/day	PA (MVPA) and SB; 1440 min; bidirectional; ActiGraph GT1M; hip	7 days, fixed with random component	10
Zhaoyang and Martire [77]	US	152	58.04	65.39 (NR)	Elders (knee osteoarthritis)	Positive affect, negative affect (NR, hand held computer); 2 prompts/day	PA (MVPA) and SB; 1440 min; bidirectional; ActiGraph GT1M or GT3X; hip	22 days, fixed	7.5

*After* affective well-being prior to physical behavior, *Before* physical behavior prior to affective well-being, *Bidirectional* physical behavior prior to and after affective well-being, *Event-based* self-initiated prompts, *Fixed with random component* random prompts within pre-established intervals or semi-random prompts, *Fixed* fixed prompts, *LPA* light physical activity, *MDD* major depression disorder, *Metric* e.g., milli-g, acceleration counts, steps, posture, *min* minimum, *max* maximum, *Mixed* e.g., activity- or sedentary-triggered prompt, combined with random prompts, *MVPA* moderate to vigorous physical activity, *NR* not reported, *PDA* personal digital assistant, *Random* random prompts, *SB* sedentary behavior

^a^Due to its novel statistical approach, this study could not be reviewed within the data extraction framework which we custom-developed to the methods applied by most of the AA studies in the PB-AWB field

### Quality Assessment/Risk of Bias Assessment

Following the guidelines for QA measure of PRISMA and the National, Heart, Lung, and Blood Institute [44, 45], our modified QA primarily aimed to assess the “risk of bias” [46] of studies included to give an estimate of how likely certain study features may have led to ambiguous results, but the QA also includes a valuation of the comprehensiveness of information given to enable replication of results. For example, and in particular, in studies of PB-AWB associations in everyday life aiming to assess associations of sedentariness and AWB, a well-known risk of bias is the (lack of reporting of the) body position of the accelerometer device [47], which may place a study at enhanced likelihood for misleading results, for example, devices attached to the hip are limited in their validity of capturing sitting versus standing postures [52]. However, according to recent guidelines [45], our QA is not primarily intended to reflect the hierarchical quality of studies, for example, via between-study rankings, but rather to detect potential flaws and thus better reflect the internal validity of studies for the risk of bias assessment. Therefore, this QA is not well suited to judge absolute discrepancies between studies. To capture all relevant features of intensive longitudinal device-based and real-time assessment studies on PB and AWB, we built upon the Checklist for Reporting EMA Studies (CREMAS) [53], reporting guidelines for AA studies in psychopathology research [54], and the National Institute of Health Study Quality Assessment Tools [44]. For example, our modified QA tool included categories such as accelerometer technology used, e-diary sampling schema applied, and compliance rates received (for details, see ESM 4). In line with the PRISMA guidelines and an established scoring approach [55], we set up three evaluation levels: high, moderate, and low risk of bias. The modified QA consists of 16 questions, with a total score of 16. In particular, a score in the range of 16–12 indicates a strong quality (i.e., low risk of bias), a score in the range of 11–6 reflects moderate quality (i.e., moderate risk of bias), and a score in the range of 5–0 indicates weak quality (i.e., high risk of bias). For details on the evaluation process, see ESM 4. Following established procedures [56], we calculated the inter-rater reliability based on a single-rating, absolute agreement, two-way mixed-effects model with two raters across 66 studies (subjects), which indicated good reliability (intraclass correlation coefficient (ICC) = 0.777; confidence interval (CI): 0.52–0.88). Each article's quality was assessed independently by two researchers (MG, IT). Any discrepancies were discussed among the authors until consensus was found.

## Results

### Study and Sample Characteristics

After removing duplicates, the systematic literature search yielded a total of 2225 relevant studies and 66 studies remained in the final selection (see Fig. 1). The additional reference screening did not yield any further studies to be included. Of these 66 studies, 62 drew from independent datasets. Participants were recruited from 11 different countries, mainly from the USA (26) and Germany (24). The mean age of participants in the studies ranged from 9.51 to 72.4 years. The total sample size of the selected studies varied between 10 and 805 participants. For an overview, see Table 1.

### Quality Assessment/Risk of Bias Assessment

**Fig. 2:**
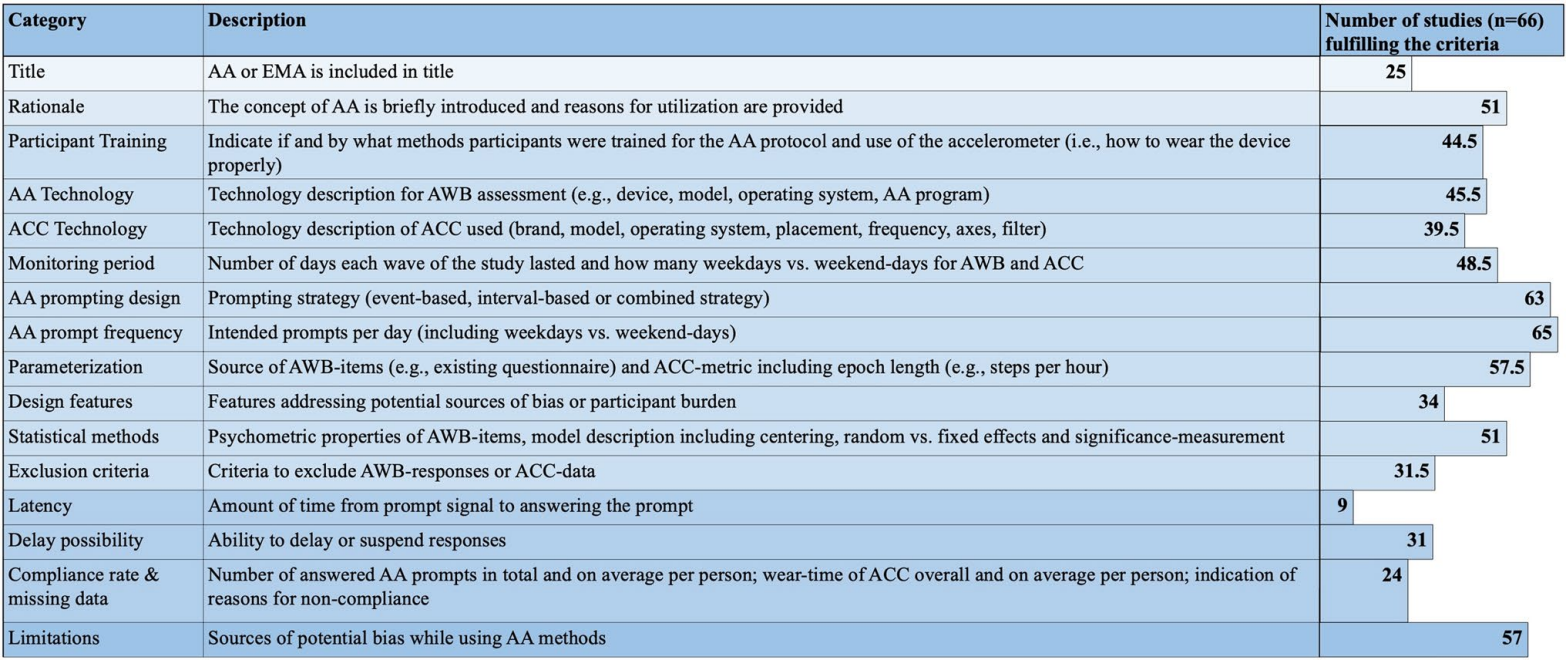
Quality assessment (QA) description and number of studies fulfilling the criteria. The modified QA is displayed with 16 different categories. The numbers of studies that report information on the respective category are listed on the right. *AA* ambulatory assessment, *EMA* ecological momentary assessment, *AWB* affective well-being, *ACC* accelerometry

For AA categories, one study was classified at high risk of bias, 50 studies showed moderate risk of bias, and 15 studies showed a low risk of bias. The mean risk of bias score was 10.27 (SD = 2.14; min = 4.5, max = 14) within a range of 0–16. Comprehensive information was provided for prompt frequency (i.e., 65/66 studies), further sampling design details, and parameterization of accelerometer data (see Fig. 2); thus, most of the information was provided for technical details such as PA epoch lengths. The epoch length choice in accelerometer studies influences activity classification accuracy. Longer epoch length may misclassify short vigorous activities as moderate. Modern technology allows for shorter epoch lengths than 60 s, which are recommended, while the ideal epoch length for health outcomes remains unclear [47]. Conversely, more than half of the studies (40/66) did not report details regarding the accelerometer sampling frequency. The sampling frequency is crucial for accelerometer data accuracy. It should be at least twice as high as the highest movement frequency component to prevent aliasing effects; for further discussion see [57, 58]. Most of the studies (48/66) did not detail how accelerometer data had been filtered, with only 18 out of 66 studies reporting whether a high- or low-pass filter were set, i.e., critical information for risk of bias assessment [47]. Before converting raw data, filters are commonly applied to remove non-human movement acceleration frequencies. Different filters are available for data processing, and their selection significantly affects the results. Providing information on the specific filters used is crucial since there are no internationally accepted standards for signal processing [59]. This lack of standardization makes direct comparisons of outcome metrics across devices difficult [60]. Moreover, nearly half of the studies (32/66) did not report details on non-wear-time definition applied, and most of the studies only sparsely reported on compliance rates, missing data, and latency (Fig. 2). For a detailed rating see ESM 5.

### Physical Behavior and Affective Well-Being Assessment

*Physical behavior* To obtain PB measurements, the majority of the included studies (24) used accelerometer devices from the manufacturer “ActiGraph” [61], followed by 16 studies using devices from the “movisens GmbH” [62], as well as other accelerometers (e.g., “varioport-e” [63]; 4) and smartwatches (e.g., “Fitbit” [64]; 5). The devices were mainly placed on the participant’s hip (30), followed by the wrist (18) and chest (11). Seven studies used multiple placement positions. The parameterization of PB included movement-based volume variables (i.e., raw acceleration data (13); activity counts (12)), time-based amount variables (e.g., minutes spent in moderate to vigorous physical activity (MVPA); 27), energy expenditure variables (e.g., metabolic equivalent; 6), as well as postural and activity-based variables (e.g., standing, stepping; 11).

*Affective well-being* The assessment of AWB (mainly implemented on smartphone e-diaries) differed between studies; 19 studies used a short version of the Multidimensional Mood Questionnaire (MDMQ; [65]). This questionnaire has been specifically adapted and validated for AA studies [38], and captures the three dimensions valence, energetic arousal, and calmness; 19 studies based their items on existing (non-AA) questionnaires like some form of the PANAS [37, 66–69], mostly assessing the two dimensions positive and negative affect; three studies applied the circumplex model [70], two studies the Profile of Mood States (POMS; [71]), and two studies the Depression and Anxiety Mood Scale (DAMS; [72]). Nineteen studies used self-developed items that were not based on standardized questionnaires, or no source was reported (see ESM 6).

### Assessment Duration and Frequency

*Physical behavior* The PB assessment duration differed among studies; mainly short periods of time were recorded, i.e., up to 7 days (42). Most of the studies aggregated PB across 30-min time frames (16), 1,440 min (16), or 15 min (13) before or after the e-diary prompt for their statistical analyses on the PB-AWB association. Of note, “aggregated time frame” refers to the time frame of PA aggregation for the statistical analysis, describing the time frame used for parameterization of PA (see Sect. 2.4 for details).

*Affective well-being* The majority of the studies (23) used a time-based sampling strategy with random components such as prompts occurring at random times within pre-established intervals or semi-random prompts. Sixteen studies chose a fixed time interval. In two studies, participants were responding to self-initiated queries (similar to an event-based sampling strategy). A combination of an event-based sampling strategy together with random or fixed prompts was applied in six studies. One study used an activity-triggered sampling scheme, while three studies utilized a sedentary-triggered design including fixed and random prompts. Two studies employed a geolocation-triggered sampling scheme including fixed and semi-random prompts. Three studies did not report the sampling schema applied. In line with the study inclusion criteria (d) (for details see Sect. 2.2), the number of prompts per day ranged from once to 23 times. Most studies applied a prompt frequency of one to seven prompts per day (42) and had an assessment duration of 1–7 days (52).

### Populations Studied

Most studies reviewed researched adult populations (50), followed by investigations of children and adolescents (16; aged 8–26 years) and elderly persons (3; aged 64–85 years). The total number of participants was 7441. Most of the reviewed studies (36) investigated healthy adult populations. They comprised a total of 4,388 participants. Interestingly, only a few studies were conducted in patient groups, for example, major depressive disorder (7), bipolar disorders (3), anxiety disorders (2), alcohol disorders (1), or attention deficit hyperactivity disorder (1), with a total of 1,104 participants. The studies that solely examined elderly people (60 years and older) had an age range of 64–85 years and a total of 285 subjects (3). In this cohort, there were other physical diseases such as knee osteoarthritis. Studies examining children (5) included participants across an age range of 8–13 years. In total, 518 subjects were studied. The studies that examined adolescents (11) included participants across an age range of 10–26 years, with a total of 1020 subjects being studied. A limited number of studies focused on participants with physiological health impairments. In particular, two were conducted in overweight or type 2 diabetes participants, one study investigated participants after joint replacement surgery, and one study dealt with breast cancer survivors or low active participants (2).

### Schematic Overview of the Findings

**Fig. 3:**
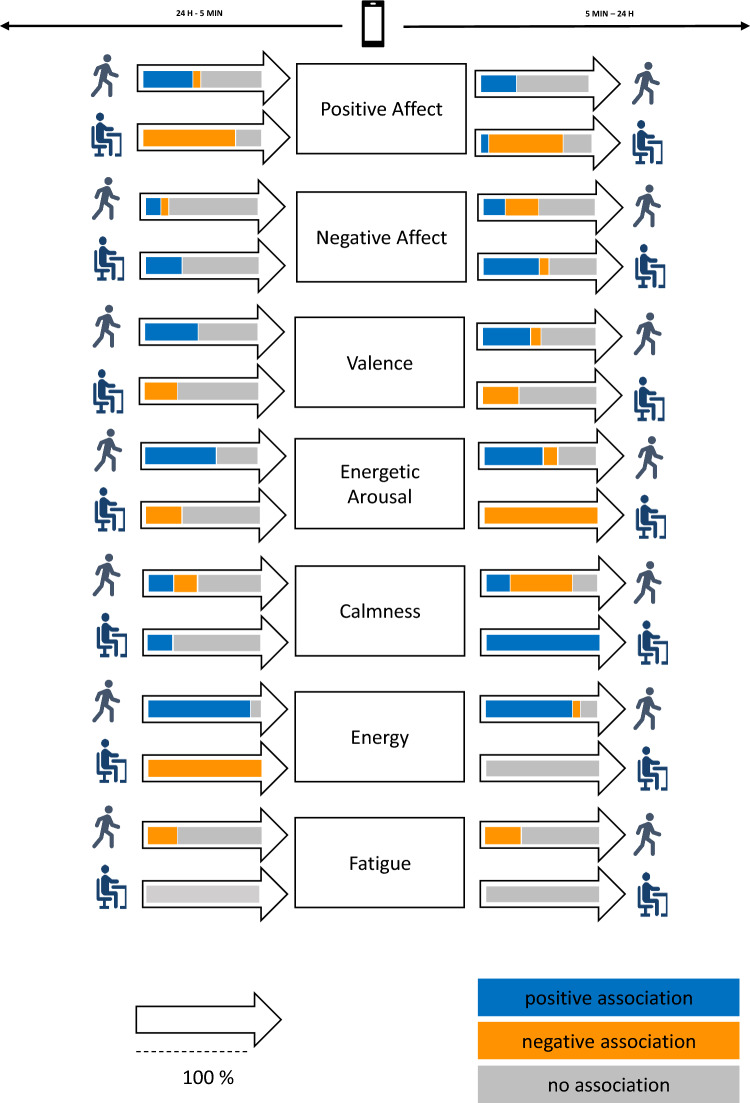
Graphical overview across the multilayered study findings reviewed. The direction of the arrows indicates the nature of the association (i.e., PA and SB being associated with subsequent affective well-being vs. affective well-being being associated with subsequent PA and SB). The color composition of the arrows represents the direction and statistical significance of the association in relation to the number of effects reviewed in percentage. That is, blue represents the relative number of effects revealing positive significant associations in % [positive beta coefficient and *P* value < 0.05]; orange indicates the relative number of effects showing negative significant associations [negative beta coefficient and *P* value < 0.05]; and gray indicates the relative number of effects receiving non-significant associations [*P* value ≥ 0.05]. For example, 45% of the effects in studies reviewed that investigated the association of PA with subsequent positive affect received a positive significant result (i.e., PA increased subsequent positive affect), 10% of the effects showed a significant negative result (i.e., PA decreased subsequent positive affect), and 45% of the effects showed a null finding (PA was not associated with subsequent positive affect); see the very top left arrow﻿. *AWB* affective well-being, *LPA* light physical activity, *MVPA* moderate to vigorous physical activity, *PA* physical activity, *PB* physical behavior, *SB* sedentary behavior

**Fig. 4:**
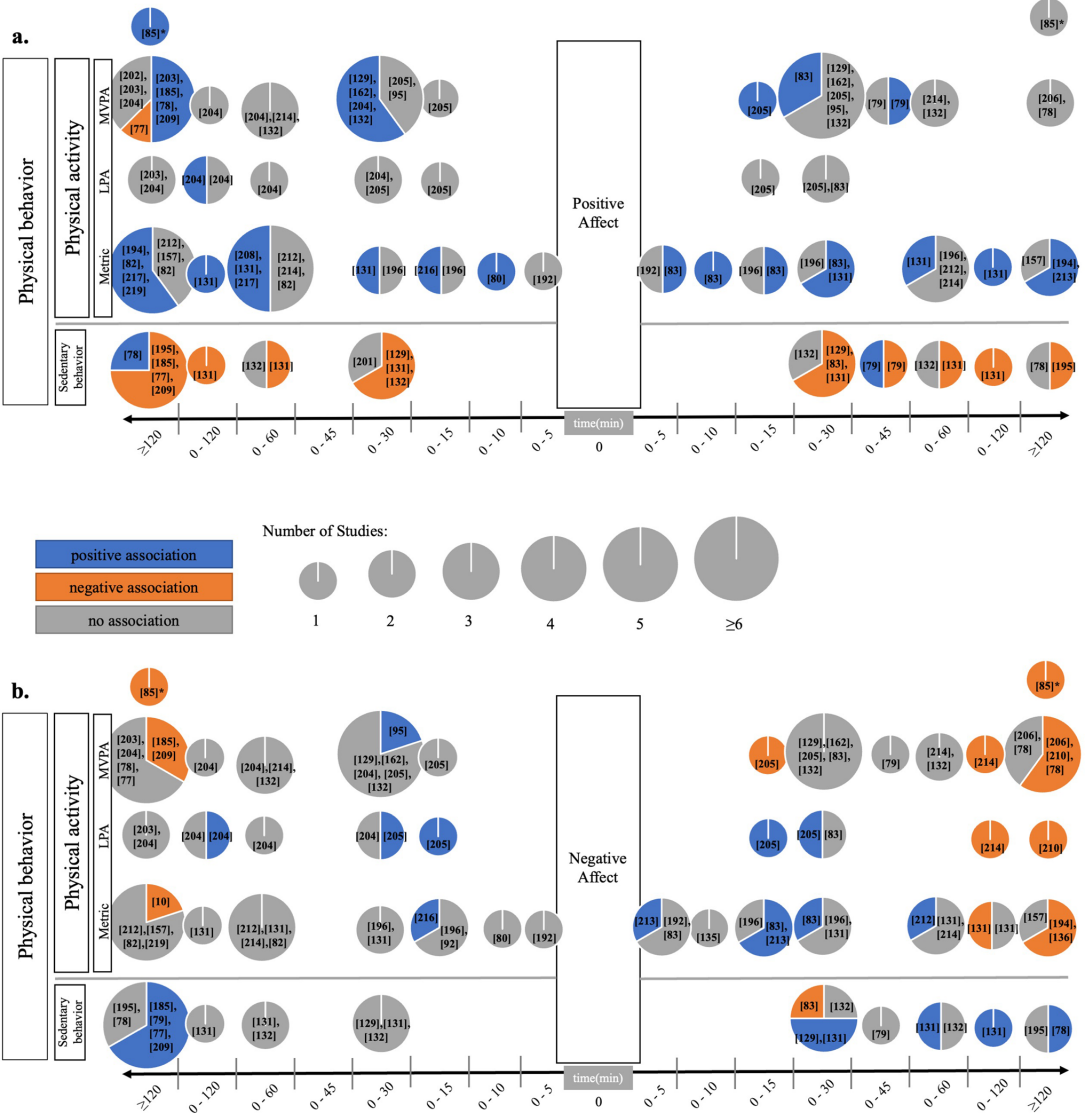
Associations of real-life PB and **a** positive affect and **b** negative affect. The *x*-axes depict the aggregated PB time frame in relation to the e-diary prompt, i.e., the time frame (in minutes) before vs. after the e-diary prompt across which PB had been aggregated (timing-order). The *y*-axes depict the PB categories applied, i.e., SB, PA parameterized in a metric unit (e.g., steps, counts, movement acceleration intensity), LPA, and MVPA. The pie charts contain three pieces of information. First, their total size represents the number of investigated relationships on the respective PB-AWB feature-combinations comprising PB and AWB quantifications, their direction, order, and timing of investigated relationships. Second, the color composition represents the investigated relationship directions, i.e., blue colors equal positive significant associations (positive beta coefficient and *P* value < 0.05), and orange colors show negative significant associations (negative beta coefficient and *P* value < 0.05) relative to all effects that investigated the respective feature combinations. Third, non-significant investigated relationships are colored grey (*P* value ≥ 0.05). *Due to the novel statistical approach (for details see Sect. 4.5), this study could not be reviewed within the framework that we custom-developed to the methods applied by most of the AA studies in the PB-AWB field. *AWB* affective well-being, *LPA* light physical activity, *MVPA* moderate to vigorous physical activity, *PA* physical activity, *PB* physical behavior, *SB* sedentary behavior

**Fig. 5:**
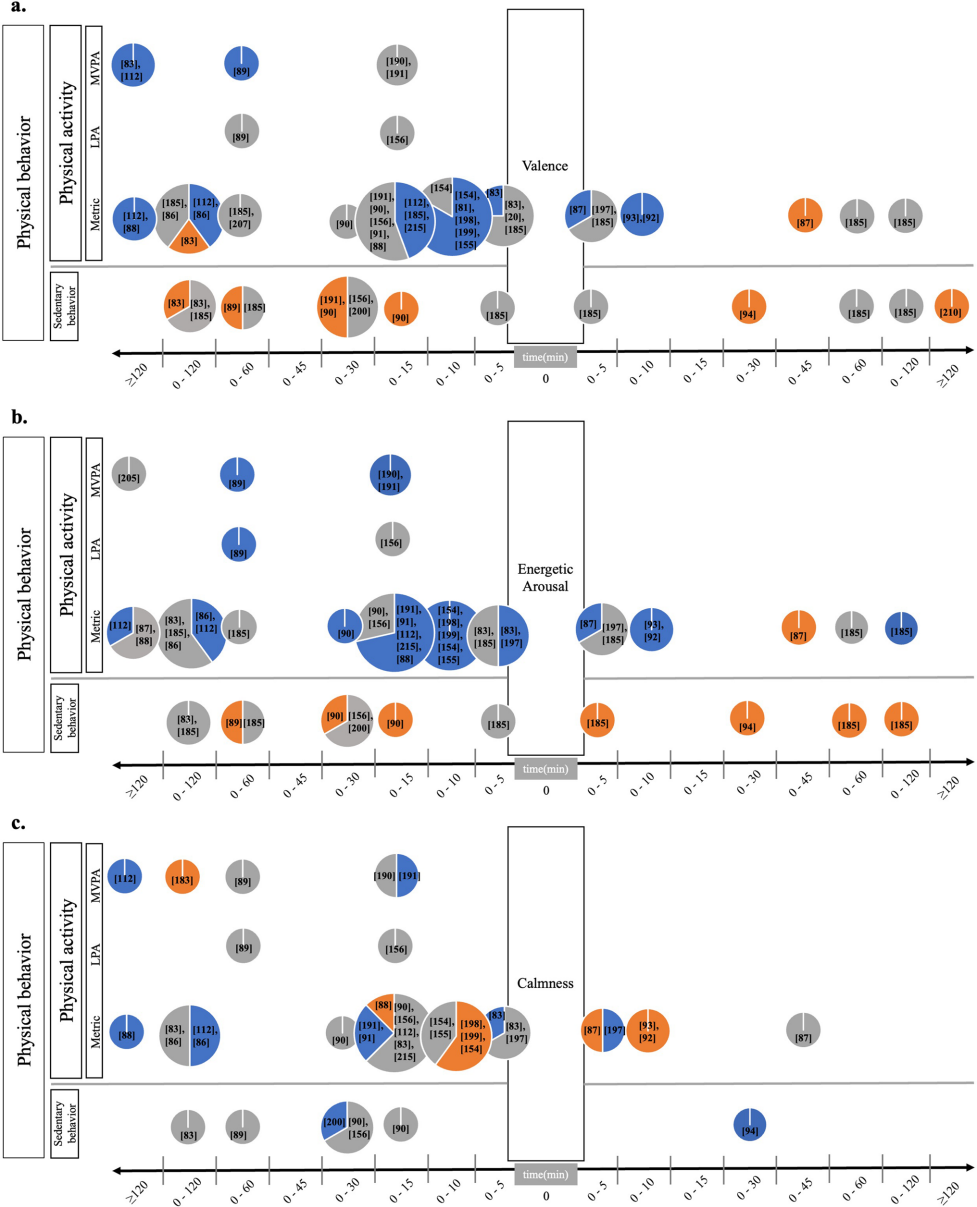
Associations of real-life PB and **a** valence, **b** energetic arousal, and **c** calmness. The *x*-axes depict the aggregated PB time frame in relation to the e-diary prompt, i.e., the time frame (in [minutes]) before vs. after the e-diary prompt across which PB had been aggregated (timing-order). The *y*-axes depict the PB categories applied, i.e., SB, PA parameterized in a metric unit (e.g., steps, counts, movement acceleration intensity), LPA, and MVPA. The pie charts contain three pieces of information. First, their total size represents the number of investigated relationships on the respective PB-AWB feature-combinations comprising PB and AWB quantifications, their direction, order, and timing of investigated relationships. Second, the color composition represents the investigated relationship directions, i.e., blue colors equal positive significant associations (positive beta coefficient and *P* value < 0.05), and orange colors show negative significant associations (negative beta coefficient and *P* value < 0.05) relative to all studies that investigated the respective feature combinations. Third, non-significant investigated relationships are colored grey (*P* value ≥ 0.05). *AWB* affective well-being, *LPA* light physical activity, *MVPA* moderate to vigorous physical activity, *PA* physical activity, *PB* physical behavior, *SB* sedentary behavior

**Fig. 6:**
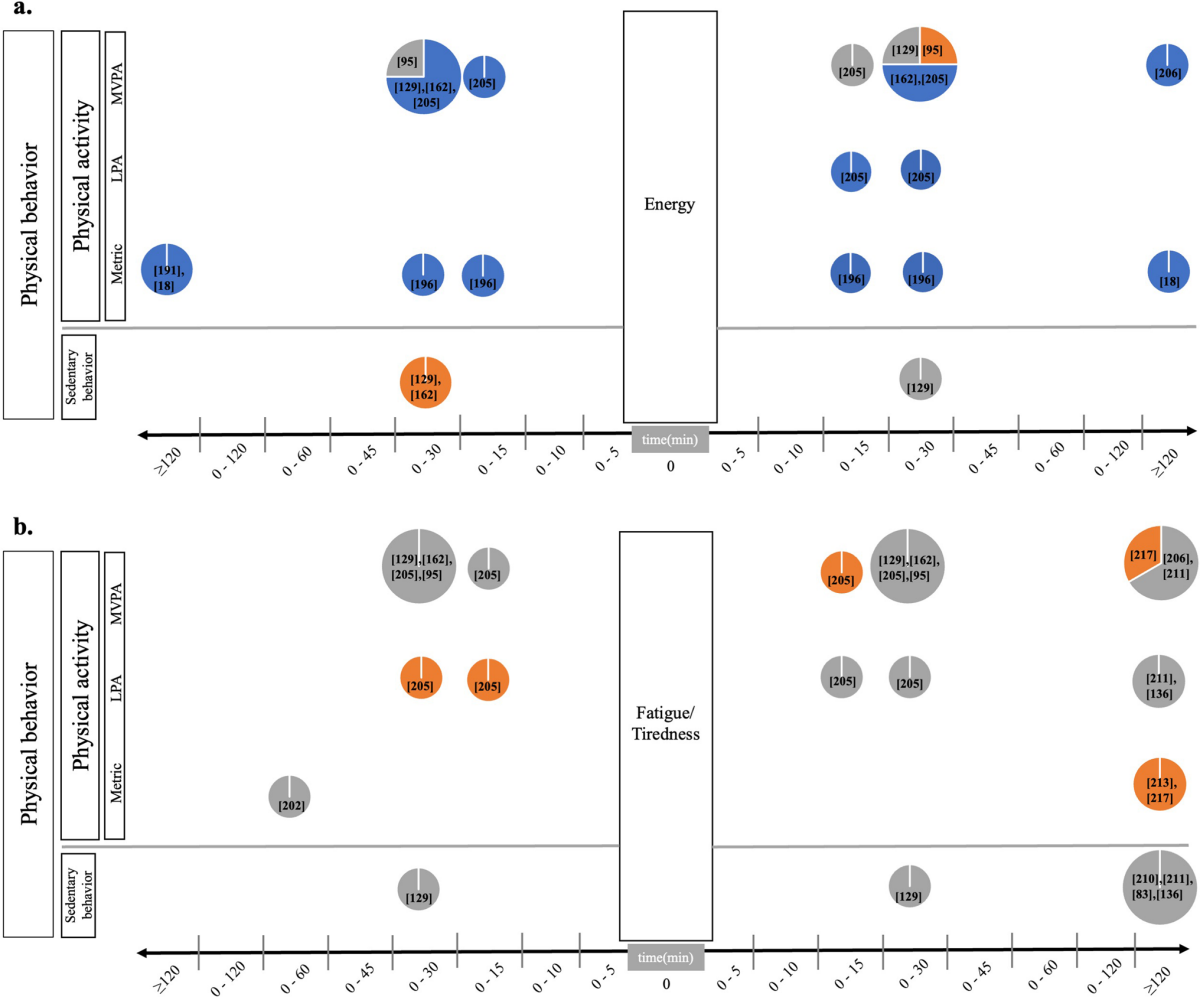
Associations of real-life PB and **a** energy and **b** fatigue/tiredness. The *x*-axes depict the aggregated PB time frame in relation to the e-diary prompt, i.e., the time frame (in minutes) before vs. after the e-diary prompt across which PB had been aggregated (timing-order). The *y*-axes depict the PB categories applied, i.e., SB, PA parameterized in a metric unit (e.g., steps, counts, movement acceleration intensity), LPA, and MVPA. The pie charts contain three pieces of information**.** First, their total size represents the number of investigated relationships on the respective PB-AWB feature-combinations comprising PB and AWB quantifications, their direction, order, and timing of investigated relationships. Second, the color composition represents the investigated relationship directions, i.e., blue colors equal positive significant associations (positive beta coefficient and *P* value < 0.05), and orange colors show negative significant associations (negative beta coefficient and *P* value < 0.05) relative to all studies that investigated the respective feature combinations. Third, non-significant investigated relationships are colored grey (*P* value ≥ 0.05). *AWB* affective well-being, *LPA* light physical activity, *MVPA* moderate to vigorous physical activity, *PA* physical activity, *PB* physical behavior, *SB* sedentary behavior

We created a series of figures that enable a graphical review of the multilayered findings on the association of PB and AWB in everyday life (Figs. 3, 4, 5, 6, see Sect. 3.7). Figure 3 introduces this methodological approach applied to review the studies’ findings. In particular, the affective well-being subcomponent quantifications most often used in the studies reviewed (i.e., positive affect, negative affect, valence, energetic arousal, and calmness, energy, fatigue/tiredness) are displayed at the center of Fig. 3. For each of these affective well-being subcomponents, their respective associations with PA and SB are visualized through colored arrows.

### Main Results

For a detailed review of the evidence, we summarized study results as a function of PB-AWB association features, i.e., the number of (non-)significant effects was plotted against their PB and AWB quantifications, their effect-directions, and their timing-orders. Some studies examined PB across multiple aggregated time frames within the same dataset. For data synthesis and to concentrate on the level of individual significant versus non-significant PB-AWB relationships found, various analyses within a study and across multiple aggregated time frames were incorporated into our results, which we called *investigated relationships* (and thus they were not summarized; e.g., a study that examined the relationship of PA and positive affect within the same data set for the aggregated time frames of 5, 10, and 60 min contributes three distinct investigated relationships into our Sect. 3). We also treated multiple effects from different studies (different papers) that used the same dataset individually, i.e., each result from each study (paper) counted as an individual investigated relationship in our results. More precisely, each investigated relationship from a study was treated as a distinct data point in our analysis, allowing us to maintain granularity in our examination of the relationships between PB and AWB. Translated into practice, some studies used the same data set to investigate different questions on the PB-AWB relationship in distinct papers; they counted as individual investigated relationships in our results, respectively. Of note, each PB-AWB association entered our analysis just once; i.e., while we included various investigated relationships from one data set reported in one paper or scattered across several papers, we did not include a single investigated relationship twice. The reviewed studies comprised a total of 242 investigated relationships for the PB-AWB direction, while less investigated relationships (i.e., 161) were available for the reverse AWB-PB association. The results are detailed in Figs. 4–6 (see also ESM 7). Moreover, to give an idea of the size of effects found in the studies reviewed, we provide a summary of practical effect sizes reported, a method also known as benchmarking and recommended for interpreting the PA effects seen in daily life [73] to indicate the meaningfulness of effects observed [74–76]. Of note, practical effect sizes had only been reported by a small portion of studies reviewed (14 studies).

#### Physical Behavior, Positive and Negative Affect

Figure 4a shows studies researching PB associations with positive affect. Most of the investigated relationships revealed either a positive association of PB with positive affect, i.e., higher PA was related to more positive affect (20/47), and more SB was related to less positive affect (7/9). Thirteen of 33 investigated relationships revealed a positive significant association of positive affect with PB, i.e., higher positive affect was related to more PA (13/33), and more positive affect was related to less SB (7/11). Sixty-nine of 102 investigated relationships in total showed non-significant associations. Only three investigated relationships showed opposite relationship directions [77–79]. Overall, this points to some evidence for a positive association of PB with positive affect in everyday life. Six studies reported practical effect sizes for the associations between PB and positive affect. In a study by Koch et al. [80], walking instead of sitting resulted in an increase of 3.2 points in positive affect on a scale with a range of 6–42. In Cushing et al.’s study, each one-unit increase in MVPA (minutes) beyond participants' usual level was associated with a 0.12-point increase in positive affect [81]. Additionally, every one-unit increase in sedentary time (minutes) beyond participants’ average level was linked to a decrease of 0.10 points in positive affect (scale 1–5) [81]. Similarly, Zhaoyang et al. [77] found days with an extra hour spent in sedentary behavior to be associated with a 0.1-point decrease in positive affect on a 7-point scale. If participants exceeded their daily average step count by 500 steps in Stevenson et al.’s study this was linked to a 0.02-point increase in positive affect on a scale of 0–10 [82]. Moreover, in Zenk et al.’s [78] study each 1-min increase in MVPA during the day was associated with a 2.2% higher likelihood of positive affect. Further findings by Schwerdtfeger et al. [83] suggest that a 3-point increase in positive affect (scale 6–30) corresponds to a 13–16% increase in bodily movement.

Figure 4b depicts studies investigating PB associations with negative affect. Overall, the picture appears heterogeneous. In particular, 12/82 investigated relationships revealed a positive significant association between PA with negative affect, i.e., increased PA led to higher negative affect (5/42), and an increased negative affect led to more PA (7/39). Conversely, 14/82 investigated relationships showed the reverse direction of significant associations, i.e., increased PA led to lower negative affect (3/42) and higher negative affect led to less PA (11/39). SB was significantly and positively related to negative affect in 8/21 investigated relationships and one study showed the reverse investigated relationship-direction [83]. In 68/103 investigated relationships there was no significant PB–negative affect relationship found. In a comprehensive and important work, Ruissen and colleagues reviewed [84] custom-developed and applied methods to better take into account composition and timing aspects of PA provoking AWB, thereby overcoming some limitations of prior studies in the field (for details see Sect. 4.6 [85]). In the resulting first study applying these procedures, Ruissen et al. reported a “recursive relationship between incidental affective states and MVPA, which is particularly strong at 7–9 h time intervals” [85]. In particular, Ruissen et al. found that the strongest cross-lagged effects of high positive incidental affect and low negative incidental affect predicting subsequent MVPA occur approximately 8 h prior to an MVPA episode. A similar timing was observed in the reverse direction of MVPA predicting subsequent affect [85]. This study's use of continuous-time modeling represents an innovative approach that promises to offer more detailed insights into the interplay between AWB and PB. Due to its alternative and sophisticated statistical approach, this study could not be reviewed within the framework that we custom-developed to the methods applied by most of the AA studies in the PB-AWB field. Thus, this study has been highlighted with an asterisk in Fig. 4a, b. Three studies reported practical effect sizes for the associations between PB and negative affect. A 3-point increase in negative affect (scale 5–25) corresponded to a 14% increase in bodily movement (counts/minute) in a study by Schwerdtfeger et al. [83]. Furthermore, in a study by Zenk et al. [78], individuals reporting negative affect (dichotomized) experienced a subsequent 38.6% decrease in MVPA and a 33.2-min increase in SB. Additionally, in Zhaoyang et al.’s study [77], spending an additional hour in SB was associated with a 0.04-point increase in negative affect on a 7-point scale.

#### Physical Behavior, Valence, Energetic Arousal, and Calmness

PB associations with valence are illustrated in Fig. 5a. Higher PA was significantly associated with more positively valenced mood (14/31) and more SB was significantly correlated with less positively valenced mood (5/11), or non-significant associations emerged (6/11). Two investigated relationships showed a reverse direction [86, 87]. Three of eight investigated relationships revealed a significant positive association of valence with PA, i.e., higher valence was related to more PA, and more valence was related to less SB (2/5). That is, overall, most of the investigated relationships revealed a significant positive association of PB with valence. Eight studies examined the practical effect sizes for PB associations with valence. Reichert et al.'s study [88] demonstrated that 2 h of exercise increased valence by 2.5 points on a 0–100 scale. Additionally, in the study by Giurgiu et al. [89], increasing MVPA by 20 min enhanced valence by 1.35 units. Conversely, decreasing SB by 20 min enhanced valence by 0.55 units, while increasing SB up to 20 min reduced valence by 1.12 units (scale 0–100) [89]. Being sedentary for 15 min instead of 5 min resulted in a decrease in valence by 3 units (scale 0–100) [90]. Breaking up SB with higher-intensity activities like moderate walking led to an average valence enhancement of 18.13 points, while low-intensity activities like standing enhanced valence by 8.29 points (scale 0–100) [86]. Furthermore, in Koch et al.’s study, choosing to walk instead of remaining seated or engaging in exercise resulted in an average increase in valence of 0.257 and 0.258, respectively, on a 1–7 scale [91]. In a reverse effect direction, a 10-point increase in valence (scale 0–100) resulted in a 4.5% increase in non-exercise activity [92]. Furthermore, a 1-point increase in a participant's valence on a 1–7 scale correlated with a substantial 19% rise in their non-exercise activity [93]. Additionally, higher valence ratings, as compared to lower ones on a 0–100 scale, were linked with reduced SB by 2.77 min [94].

For the association of PB with energetic arousal (see Fig. 5b), 18/28 investigated relationships showed that more PA correlated significantly with higher energetic arousal, and 10/28 investigated relationships were non-significant. Similarly, more SB was significantly correlated with lower energetic arousal (3/9), and 6/9 investigated relationships revealed no significant association between SB and energetic arousal. There was a positive association of energetic arousal with PA, i.e., higher energetic arousal was related to more PA (4/8), and more energetic arousal was related to less SB (4/4). Only one study showed a reverse investigated relationship direction [87]. Overall, this rather homogenous picture points toward a positive association between PB and energetic arousal. Eight studies reported practical effect sizes for PB associations with energetic arousal. In a study by Reichert et al. [88], there was an increase of 14.8 points on a 0–100 scale in energetic arousal when participants walked 15 min instead of remaining seated. Koch et al. [91] found that choosing to walk instead of remaining seated resulted in an average increase in energetic arousal by 0.136 (scale 1–7), while engaging in sports decreased energetic arousal by − 0.574 points on a 1 to 7-point scale. Breaking up SB with low-intensity activities, like standing, enhanced energetic arousal by 11.69 points, while higher intensities, like moderate walking, enhanced energetic arousal by 25.58 points on a scale of 0–100 [86]. Furthermore, a 20-min increase in MVPA enhanced energetic arousal by 1.31 units on a scale of 0–100 [89]. Conversely, reducing SB by 20 min increased energetic arousal by 1.68 units, while increasing SB up to 20 min resulted in a decrease of 3.39 units (scale 0–100) in energetic arousal [89]. Moreover, being sedentary for 15 min instead of 5 min led to a decrease in energetic arousal by 7.6 units (scale 0–100) [90]. Reichert et al. [92] reported in their study that feeling 10 points more energized (scale 0–100) was associated with a 15.2% increase in non-exercise activity. Additionally, a 1-point increase (scale 1–7) in energetic arousal led to a 20% increase in non-exercise activity [93]. Furthermore, higher energetic arousal ratings (e.g., 90), compared to lower ones (e.g., 20) on a 0–100 scale, were associated with a reduction in sedentary time of about 4.45 min [94].

Figure 5c depicts a heterogeneous picture of the results of the studies investigating PB associations with calmness. In particular, 7/26 investigated relationships revealed a significant positive association of PA with calmness; five studies showed the reverse investigated relationship-direction. SB was significantly and positively related to calmness in 1/1 investigated relationship. In addition, 1/5 investigated relationships revealed a significant positive association between calmness and PA; 3/5 of the studies showed the reverse investigated relationship-direction. Calmness was significantly and positively related to SB in 1/1 investigated relationship. Twenty-one of 32 investigated relationships showed no significant PB-calmness relationship. Interestingly, thus far only few studies investigated correlations of calmness with subsequent PB compared to other PB-AWB feature-combinations. Six studies provided practical effect sizes for the associations between PB and calmness. In a study by Reichert et al. [88], participants experienced a decrease of 7.2 points in calmness when choosing to walk for 15 min instead of remaining seated; 2 h of exercise increased calmness by 2.4 points (scale 0–100). Furthermore, choosing to walk instead of remaining seated or engaging in exercise resulted in an average decrease in calmness by − 0.117 and − 0.280, respectively, on a 1–7 scale [91]. Breaking-up SB with low-intensity activities such as standing was associated with an increase in calmness by 7.65 points. Higher PA intensities such as moderate walking were related to enhanced calmness by 16.74 points on a 0–100 scale [86]. In their study, Reichert et al. [92] showed that a 10-point increase in calmness (scale 0–100) led to a decrease in non-exercise activity of 9.7%. Moreover, when participants felt 1-point more calm (scale 1–7), their subsequent non-exercise activity was decreased by 15% [93]. In addition, higher ratings of calmness compared to lower ratings on a 0–100 scale were associated with higher amounts of sedentary time of about 5.54 min [94].

#### Physical Behavior, Energy, and Fatigue

The most homogenous picture appeared for associations of PB with energy (see Fig. 6a). That is, out of a total of 23 investigated real-life PB-energy relationships, 19 were significant. Most investigated relationships revealed PB to be significantly and positively correlated with feelings of energy; namely 8/9 investigated relationships showed higher PA to be associated with more energy, and 2/2 investigated relationships found that more SB was significantly related to less energy. In contrast, only 1/11 investigated relationships showed an opposite direction with more energy being significantly associated with less PA [95]. One study reported practical effect sizes on the association between PB and energy. It revealed that each one-unit increase in MVPA (minutes) beyond participants' usual level correlated with an increase of 0.06 units in energy (scale 1–5); for every one-unit increase in sedentary time (minutes) beyond participants’ usual level, a decrease of 0.05 units in energy was observed [81].

The studies on PB-fatigue/tired associations (Fig. 6b) showed either a significant negative investigated relationship direction, or non-significant associations (6/29). In particular, more PA was significantly related to less fatigue/tiredness (29), and more fatigue/tiredness was significantly related to less PA (4/15). All investigated relationships on SB and fatigue/tiredness showed non-significant relationships (0/5).

#### Physical Behavior and other Affective Well-Being Quantifications

The results for the PB-AWB association for rare AWB quantifications (i.e., sad, depressed, pleasure, anxiety, anger) are detailed in ESM 7.

## Discussion

### State of Evidence

Our synthesis of study findings revealed hardly any systematic effect of PB intensity in daily life PB-AWB associations of both temporal directions, while the review of other PB characteristics (such as duration and type) was hampered by methodological limitations in the field, which are currently being tackled. However, most studies investigated primarily incidental and unstructured PB (e.g., climbing stairs [96]), and found positive PB-AWB and AWB-PB associations even for low intensities. Incidental activities are characterized by relatively low energy expenditure, for example, gardening (metabolic equivalent: 3.8 [97]), and differ from volitional and structured PA, for example, playing handball (metabolic equivalent: 8.0). In a similar vein, differentiating the reviewed studies by AWB characteristics (such as emotions, affective states, and mood captured via different questionnaires) did not result in a clear overarching pattern for the reciprocal PB-AWB associations in everyday life. However, associations of PB with feelings of energy were homogenous across nearly all investigated relationships, implying a dominant role of subjective energy in interactions with PB in everyday human life. Of note, PB-AWB associations of both temporal directions appear to be dependent on population characteristics. For example, in people susceptible to mental disorders, a high relative number of significant investigated PB-AWB relationships were found compared to other populations. However, overall, findings were heterogeneous across investigated relationships, and our review raises the question of potential sources for this heterogeneity. Numerous reviews and meta-analyses on correlational, experimental, and quasi-experimental studies concluded that PB and AWB are positively related (e.g., [23, 24, 98]. Nevertheless, the everyday life perspective on the PB and AWB association had not been addressed for an extended period of time. It is important to emphasize that there are inherent differences between studies conducted in controlled laboratory and/or intervention settings versus AA investigations that specifically focus on real-life scenarios in real time, bypassing distortions seen in the laboratory [99]. It is not only experimental control in laboratory and interventional studies that contrasts with ecological validity of real-life studies but, for example, also the primary subject being researched; that is, structured exercise sessions in laboratory/interventional studies versus incidental PA comprising exercises as one of many PA facets in real-life studies. Therefore, we will discuss to what degree PB and AWB are related to each other in everyday life, and in both temporal directions, for example, depending on PB/AWB characteristics, contextual factors, and biological determinants. While we found more daily-life investigations of PB investigated relationships on AWB (242 vs. 161 for AWB-PB) in the present literature search, both temporal sequences of the PB-AWB phenomena promise to hold high relevance for human physical and mental health; for example, recent dual-process models and hedonism theories [8] on behavioral processes acknowledge this potential. The following section discusses both temporal sequences against the background that the observational data reviewed precludes any causal conclusions.

### Does the PB–AWB Association Differ Depending on PB Characteristics?

*Physical behavior intensity* In our review, we found heterogeneous associations between PB characteristics (e.g., activity intensity, duration, volume) and AWB. From a theoretical perspective, for example, one could have expected distinct influences of PB intensity on AWB. For example, the prominent inverse-U hypothesis [100, 101] suggests that especially moderate intensities provoke positive AWB in contrast to vigorous intensities, which are hypothesized to be associated with negative AWB. However, in general, our review showed no clear pattern of distinct effects of different PB intensities in the PB-AWB associations across daily real-life studies (e.g., comparing SB vs. LPA vs. MVPA).

Reviewing recent empirical evidence, the most prominent meta-analysis examining acute aerobic exercise on positive activated affect in the laboratory found positive affective responses at lower intensities compared to moderate or high-intensity exercise [24]. In contrast, in a meta-analysis examining regular aerobic exercise, the strongest positive effects occurred at low but also in highest intensities [23]. In line with these heterogeneous empirical findings, in a very recent meta-analysis compiling all correlational, experimental, and quasi-experimental studies investigating effects of PA on subjective well-being, activity intensity did not qualify as a significant moderator of effects [98].

We could not clearly determine a moderating role of activity characteristics on PB-AWB associations in the reviewed real-life studies via visual inspection, which is in line with other works that summarize laboratory, correlational, experimental, and quasi-experimental studies as outlined above. The heterogeneity of findings and the missing moderating role of activity intensity may be linked to a wealth of confounders. For example, following the dual-mode theory, fitter individuals may be more likely to benefit from high-intensity exercise [102].

The different results regarding the intensity of exercise are possibly due to the individual perception of the exertion of PA with the associated phenomena such as increased heart rate and rise in blood lactate [103–105]. These physiological responses to PA stimuli are dependent on the individual's fitness state and thus contribute to the degree of fatigue perceived during PA [102, 106, 107]. Untrained individuals will perceive the physical processes as more fatiguing than more trained individuals and thus generate a differentiated affective response [108, 109]. Therefore, one may be tempted to speculate that in studies including participants with heterogeneous fitness levels, not controlling for those may confuse effects, and similarly in real-life studies [110, 111].

In support of the idea that a wealth of confounders hide intensity effects within the PB-AWB associations, two real-life studies contrasting extreme forms of PB intensities found distinct effects on AWB. In particular, opposing incidental PA versus volitional PA revealed both in an investigation by Koch et al. [91] and an independent study by Jeckel and Sudeck [112] distinct effects, that is, volitional PA increased affective valence and calmness, but incidental PA increased energetic arousal. However, of course, one has to acknowledge that unstructured PA activity versus structured PA do also differ, in motives, duration, and social interaction, which limits our conclusion.

*Physical behavior duration* At a theoretical level, there are assumptions of an optimal PB duration for mood-enhancing effects. For example, a minimum PB duration to provoke effects on the central nervous system has been hypothesized to be necessary to improve AWB [113]. Conversely, extended durations of high-intense PA have been suggested to potentially induce fatigue, leading to a decline in AWB [114]. Distinct behavioral (e.g., stress response), neurophysiological (e.g., activity in prefrontal cortex or hippocampus [115, 116]), and neurochemical (e.g., lactate, cortisol, neurotrophins [117]) processes [104] have been researched and discussed as relevant for potential duration effects on the PB-AWB association.

Studying recent empirical evidence, a review including 38 studies showed such saturation effects between PB and AWB [118]. The tendency of the included studies showed that 10–30 min of PA had sufficient positive effects on AWB and longer sessions do not necessarily lead to additional benefits. This is in line with previous studies conducted in the laboratory regarding optimal affect response in terms of duration that have mainly been oriented towards short, 20-min PA periods [113]. In a prominent study that investigated duration effects, Ekkekakis et al. found an inverted-U dose–response relationship between PA duration and affect [100]. However, a meta-analysis examining effects of regular aerobic exercise could not find a specific exercise duration that was especially relevant to increase positive activated affect [23]. Similarly, in another meta-analysis across 158 laboratory studies focusing acute aerobic exercise on positive activated affect, the authors concluded that the reviewed evidence provides “support for the hypothesis of no differential effect of exercise duration on post-exercise positive activated affect” [24]. The interpretation of PB duration effects of studies reviewed in the present work must be viewed against the background that the investigation of the role of specific PB components (such as duration, intensity, and type of PA) and the aggregated time frames of effects within the PB-AWB associations in everyday life are known methodological challenges in the field, which is especially prominent with regard to the duration component (for details see Sect. 4.6 below). Accordingly, we could not draw general statements within this review, for which duration of PA and in which time periods in everyday life an optimal relationship between PB and AWB emerged. While overall the dose–response relationships of the duration of activity on AWB are difficult to infer from laboratory studies to everyday activities, some daily-life studies did specifically investigate duration effects and give first insights. For example, a study by Giurgiu et al. [86] showed that the duration of interrupting SB had no effect on AWB. Moreover, in a recent work, Ruissen and colleagues reviewed [84] custom-developed and applied methods (i.e., Bayesian hierarchical continuous-time structural equation models) for overcoming methodological challenges [85]; in the resulting study applying these procedures, Ruissen et al. found a relationship between incidental affective states and MVPA, which is particularly strong at 7- to 9-h time intervals.

### Does the PB-AWB Association Differ Depending on Affective Well-Being Characteristics?

PB may affect distinct components of AWB differently and vice versa. For example, there is evidence that effects of PA on positive affect are stronger than those on negative affect, at least in healthy populations [41]. However, reviewing the AA studies on a potential moderating role of the AWB dimension on the strengths of effects, we could not determine a clear overarching pattern. Of note, across the studies we reviewed, the quantification of AWB definitions fluctuates considerably: 28.8% used the MDMQ [38], 28.8% used the PANAS [37, 68], 4.6% used the circumplex model [70], 3% the POMS [71], 3% the DAMS [72], and 12.1% used self-developed questionnaires. In particular, the MDMQ quantifies affective well-being as a three-dimensional construct (i.e., valence, energetic arousal, calmness; for a detailed factor structure, empirical evidence, and discussion see [38]), while the PANAS builds upon an understanding of AWB as a two-dimensional construct. Within the reviewed studies using the PANAS as a basis, the items applied differed considerably even though they refer to PANAS as the same source (see ESM 6).

Most consistently, in this review, we found PB-AWB associations for the subjective energy dimension compared to all other AWB measures. Most of the PB-subjective energy associations investigated were statistically significant (19/23). Reviewing recent empirical evidence, in the meta-analysis of Reed and Ones [24], the effects found suggest that exercise led to increased positively activated affect—this was also evidenced for the effects of regular PA on positively activated affect [23]. Positive activated affect was described as a status of positive valence and activation [119], with the latter construct also being described as energy in previous studies [120]. That is, positive activated affect does not only capture affective components of valence but also comprises those of feeling energetic. For example, the PANAS questionnaire comprises the items “active, alert, …,” which clearly belong to this domain. Therefore, as already discussed in the work of Reed and Ones [24], it remains unclear whether the increases seen in positive activated affect through PB originate from affective components of valence or rather from those of feeling energetic. Against this background and the additional evidence generated in our review, we argue that especially feelings of energy seem to play a dominant role in interaction with PB in everyday human life.

From a mental health perspective, this PB-energy association has been argued to play an especially important role for patients with affective disorders. For example, one study [18] investigated the relationship between motor activity and subjective feelings of energy in bipolar patients (bipolar disorder I, II) and people with major depressive disorder. Bidirectional correlations were found between motor activity and subjective energy levels, while the association with momentary mood was unidirectional, implying a major role of the PB-energy association within individuals. This suggests that interventions aimed at increasing energy and activity might be even more beneficial than treatments aimed solely at mood elevation or stabilization in bipolar disorder and major depressive disorder.

Taking a neurobiological perspective, a recent study investigated non-exercise activity’s role in the regulation of AWB [19] and its neuronal correlates. Here, mean non-exercise activity levels were related to gray matter volume of the subgenual anterior cingulate cortex, a neuronal region shown to be involved in both affect regulation and risk for and recovery from mood disorders [19]. In everyday life and captured via AA methods, low subgenual anterior cingulate cortex gray matter volume predicted greater non-exercise activity vulnerability, leading to greater negative within-person influence of non-exercise activity on energy, while, in addition, the data indicated that people with low subgenual anterior cingulate cortex volumes also had greater energetic benefits when they achieved high levels of non-exercise activity. Put simply, participants showing neuronal risk markers for affective disorders compared to those with more resilient brain structures felt less energetic in their everyday life when being inactive but more energetic when engaging in PA. These momentary within-subject associations were related to trait well-being: for example, momentary subjective feelings of energy in real life predicted well-being captured via the established WHO-5 questionnaire and a range of other trait well-being indices [19, 121, 122]. These findings strengthen the conclusion that the PB-energy association may be of high value for prevention and treatment of affective disorders if such findings can be replicated in patient samples.

Moreover, in our review, 50 of 101 investigated relationships between PB and positive affect were statistically significant. As discussed above, these findings may be partly attributable to energy effects. Despite the different questionnaires and items used for positive affect quantification, evidence was found for associations between PA and subsequent within-subject positive affect, for example, when people were more active, they reported significantly higher levels of positive affect. For instance, after PA individuals felt more inspired, happy, and active. It seems worthwhile for future investigations to investigate effects of PB on positive affect components not linked to energy domains. First approaches to develop questionnaires specifically capturing affective responses to PA have already been developed [123]; however, to the best of our knowledge such instruments are not yet available for within-subject measurements.

Beyond positive affect, in our review, the evidence for PB affecting other domains of AWB and vice versa is mixed. For negative affect, only 34.3% (35/102) of investigated relationships were significant. These null findings are consistent with several other studies that found no significant within-subject association between PA and negative affect [41, 124]. However, some results indicated an inverse relationship between PA and depressive states [125–127]. Since only high negative values are entered in the PANAS questionnaire, information on low-activated negative states (e.g., fatigue) is not available [128]. Due to the null-findings between PB and negative affect, it might be advisable to split the construct of negative affect into single items (e.g., [129]) and thus allow low activation items.

Valence, a construct related to both positive affect and negative affect, however, is clearly different in its factor structure [38], for example, presenting no energy-related construct. In particular, associations between valence (e.g., feeling well and content) and prior time spent in PB or on subsequent PB were inconsistent; most investigated relationships were non-significant for PA predicting within-subject valence or valence predicting subsequent PA. Only 42.5% (17/40) of investigated relationships were significant. Concerning the link between PB and calmness, as a low activated positive AWB construct, the evidence was mixed and in part contradictory, i.e., some investigated relationships in our review were significant and revealed positive effects of PB on calmness (25.9%) while others revealed PB decreases calmness (18.5%) or vice versa. Concerning the link between PB and negative affect components, PA was not related to feelings of fatigue, tiredness, anger, anxiety, depressed feelings, or stress in most of the investigated relationships.

### Does the PB-AWB Association Differ Depending on Subgroups Researched?

This review shows that despite community based-samples of adult and youth populations, subgroups such as clinical samples are under-represented. Taken together, the few existing studies on subgroups are currently too small to draw overarching conclusions. However, the few investigations on vulnerable samples yield the impression of an increased relevance of the PB-AWB interaction in everyday life compared to healthy populations, especially in the mental health domain. For example, AA studies provide first mechanistic insights into the importance of PB-AWB associations for affective disorders; PB-AWB relationships seem to play an especially critical role in people showing brain structure characteristics of vulnerability for affective disorders [19] and in patients with bipolar disorder [18], but the underlying behavioral mechanisms remain to be investigated in depth. For example, we found only one study researching the PB-AWB association in patients with attention deficit hyperactivity disorder. This is surprising since alterations in both the PB and the AWB domain are central diagnosis criteria of the disorder. In a similar vein, Koch et al. [80] uncovered interactions of attention deficit hyperactivity disorder types and PB-AWB interactions in everyday life. In particular, patients being inattentive and hyperactive showed stronger PB-AWB associations compared to patients being predominantly inattentive and compared to healthy controls. Similarly, negative affect was related to PB solely in participants with a combined presentation, but not in the other two groups, which may, taken together, point towards a mechanism reinforcing hyperactivity, if replicated.

Furthermore, 16 of 66 studies investigated the PB-AWB association in children and adolescents. In particular, three of these studies found evidence for a positive association of PA and subsequent positive affect in children [130–132], and two studies showed higher levels of positive affect positively influence subsequent PA [79, 131]. Especially children who are overweight or obese benefit from increased PA and reduced SB resulting in elevated positive affect [131], which in turn may provoke sustained PA behavior [111] and thus potentially foster long-term mental and physical health. Evidence on the within-subject associations between PB and affective states at the ages of 13–18 years is mixed and points towards an idiographic relationship in this target group, suggesting that the association is unique to each individual and varies based on personal experiences, environmental influences, genetic factors, and other factors [84, 133, 134]. Further studies in adolescent samples are necessary to determine which particular PB and AWB components are related to each other and under which contextual conditions. Towards this aim, a reviewed study involving adolescents has already demonstrated that various PA motives (competitive vs. non-competitive PA) can yield diverse effects on AWB components. For instance, participating in non-competitive PA, such as skating as a leisure activity, resulted in increased feelings of energy and positive affect. In contrast, engaging in competitive PA, such as a volleyball match, led to a decrease in subjective energy [91]. In the elderly, our literature search yielded only three studies [77, 135, 136]. These three investigations provided mixed findings and therefore we are unable to draw any specific conclusions. In sum, future research endeavors exploring the (change of the) PB-AWB association across the lifespan (e.g., via a cohort studies) are highly warranted.

### Does the PB-AWB Association Differ Depending on Methodological Study Quality?

Based on our modified version of the QA, most studies were rated with a low or moderate risk of bias. Here we discuss selected categories from the QA such as PB measurement, AWB assessment, design features, compliance, and statistical modeling.

*PB measurement in AA studies* The terminology “physical behavior” (as discussed above, see Sect. 4.3), describes a recent scientific model [52] operationalizing highly complex human PB characterized by distinct features such as PB *type* (e.g., walking, standing, or sitting), *intensity* (e.g., LPA or MVPA), *purpose* (e.g., structured exercise or gardening), and *social-environmental context* (e.g., jogging alone through the city park or sitting with friends while watching movies). Here, various definitions and operationalizations exist while no consensus across disciplines has been reached thus far [137–139]. However, the variation of PB operationalization can influence the findings on PB-AWB associations [47]. In this review, we only included studies comprising a device-based PB measurement method to capture features as objectively as possible, i.e., without (retrospective) distortions from cognitive heuristics [28]. The detailed advantages and disadvantages of device-based versus self-reported PB methods are discussed elsewhere [47, 48]. Although accelerometry is broadly accepted as the gold-standard for device-based PB measurement methods in daily-life studies [47, 48, 52, 137], this method also comes with considerable degrees of freedom, challenges, and limitations in data assessment, preprocessing, and analyses, which lead to a wealth of different PB parametrizations that can influence the findings on PB-AWB associations [47].

In particular, as outlined in Sect. 3, the PB assessment design varied considerably between the studies reviewed within this work; for example, regarding (i) the *placement of the accelerometer* (e.g., hip-, wrist-, thigh-worn), (ii) the *devices used* (e.g., Actigraph, movisens Move), (iii) the *sampling frequency* (e.g., 10 vs. 30 Hz) defined, (iv) the *epoch length* installed (e.g., 1 vs. 10 vs. 600 s), (v) the *assessment duration* applied (e.g., 1 day to 3 weeks of accelerometry) and (vi) the *metrics or parameters* calculated with different software packages and distinct filtering algorithms, cut-offs, etc. (e.g., resulting in counts, movement acceleration intensity [milli-g], energy expenditure [metabolic equivalent], activity intensity [minutes spent in light, moderate, vigorous PA], body position/posture [sitting, standing, lying], activity patterns [activity breaks]).

As described earlier (see Sect. 3.3), the parameterization of PB included movement-based volume variables (i.e., raw acceleration data), time-based amount variables (e.g., MVPA), energy expenditure variables (e.g., metabolic equivalent), as well as postural and activity-based variables (e.g., standing, stepping). Each of these features has been shown to have an influence on PB quantification. For example, thigh-accelerometry has been shown to be most valid for SB assessment [52], length of measurement time frames have been associated with validity and reliability of data [140, 141], epoch lengths were recommended to be chosen as short as possible to represent spontaneous and short movement episodes adequately [142, 143], and the choice of cut-points should follow established recommendations to enable unbiased PB assessment [140, 144]. The influence of these features on PB quantification is discussed in a huge wealth of accelerometry literature (e.g., [145–147]. Therefore, obviously, the differences regarding these features of studies reviewed within this work may lead to heterogeneous findings on the PB-AWB association in both directions.

*AWB assessment in AA studies* There are extensive and ongoing discussions as well as empirical analyses on the advantages and limitations of different AWB quantifications, tackling constructs such as (core) affect, mood, and emotions. Beyond literature on these general conceptualizations, there is also considerable work on the application of AWB quantifications to the PB-AWB association. For example, in 2013 Ekkekakis summarized knowledge on this challenge in “A guide for health-behavioral research” [39], and just recently developed and validated an “Affective Exercise Experiences (AFFEXX) questionnaire” to enable the assessment of affective and motivational antecedents of PB [123]. However, this work mainly stems from laboratory and cross-sectional research, and its transfer into momentary, within-subject processes on the PB-AWB association that are central to this review is pending. In daily-life research and especially in the studies reviewed here, different questionnaires to quantify AWB, such as the two-dimensional PANAS [37] and the three-dimensional MDMQ [38], have been applied. For example, the often-used MDMQ for PA-AWB investigation in daily life, originally a German language questionnaire with 20 items, was specifically adapted for use in AA studies aiming to reduce the participant burden [38]. The resulting AA questionnaire was reduced to six bipolar items, representing the three mood dimensions valence, energetic arousal, and calmness validated to represent distinct mood components and showing high reliability for the assessment of mood changes on both the momentary within-subject (state-like) and between-subject (trait-like) level [38]. Recent work compared this MDMQ questionnaire with PANAS-like questionnaires on e-diaries, which were used in two studies reviewed. In particular, for the usage in these PA-AWB studies, the PANAS, which was not initially developed for AA studies and consisted originally of 20 items (10 positive affect/10 negative affect), had been reduced to the shorter form, for example, including 10 items (I-PANAS-SF). Such comparisons show the challenges that accompany the usage of different mood assessments. For example, the PANAS items forming the negative affect dimension offer hardly any variance in healthy samples, which can lead to ceiling effects and non-normally distributed residuals in multi-level analyses. On the one hand, this offers multiple insights into how distinct AWB components interact with PB and vice versa in everyday life. On the other hand, given that the number of studies applying the same AWB measurement is small, this precludes us from drawing overarching inferences from the studies reviewed. Therefore, beyond our call for more studies on distinct components of AWB, future investigations may be guided by key questions such as: (i) was the questionnaire developed for the purpose applied; (ii) is the questionnaire appropriate for the target group researched (e.g., clinical vs. community-based samples); (iii) is the questionnaire suitable for answering the research question (e.g., is one interested in energetic antecedents vs. tension responses of PB); and (iv) is the questionnaire validated for use in daily-life research (e.g., within-subject reliability on e-diaries). Here, the use of the “Experience Sampling Method Item Repository”, an open database including all AWB items of published daily-life studies, their fit to already existing questionnaires and their psychometric properties, may guide future studies.

*Design features, compliance issues, and statistical methods* In daily-life research on PB-AWB associations, compliance is defined as the ratio of answered versus triggered e-diary prompts and of wear versus non-wear time of the accelerometers [48]. Compliance is a measure of participant adherence to the study protocol, in particular, to the sampling schema. Therefore, AA compliance obviously depends on both the sampling schema applied and participant motivation [148]. Of the studies reviewed, nearly half of the investigations did not provide details on compliance of the accelerometer measurement, and 26 did not report any details, although this information is crucial to assess the quality and representativeness [47]. For example, since the acceleration values between sitting still and not wearing the device are almost similar, it is important to differentiate between wear and non-wear time. Only two studies reported details about the total wear time across all participants, the total wear time per participant, and reasons for noncompliance. In the studies reviewed, the average e-diary compliance rate was 79.17% (mean; SD = 29.0), ranging from 58.6% to 95%. This falls within the range of sound compliance rates according to current method guidelines [54]. Moreover, only seven studies reviewed reported their latency, with latency being defined as the time window from the e-diary prompt to the participants answering the respective prompt. This non-reporting had already been criticized in previous reviews [53, 149, 150] and is critical since high latency (such as 1 h) reduces the ecological validity and increases the probability of retrospective biases. Therefore, we suggest future studies thoroughly report accelerometer wear time, e-diary compliance, and other adherence measures such as latency; for a detailed overview see current reporting guidelines for AA studies [54]. Additionally, in study conceptualization especially sampling schemes should be carefully designed. For example, a large proportion of adults worldwide fail to meet the recommended PA levels [1]. Consequently, their everyday life is characterized by a high prevalence of sedentary behaviors, possibly with only infrequent instances of moderate to high physical activity [151, 152], which leads to restricted within-subject variance of PA [153]. This appears critical to consider in research on PB-AWB associations, for example by using activity-triggered e-diaries to enhance within-subject variance of interest [153] in PB. To capture these phases of high PA, it can be useful to apply triggered e-diaries (e.g., *activity-, *GPS-, *sedentary-triggered [86, 88, 90, 92–94, 154]) beyond fixed or random sampling designs, which draw from technological advances of accelerometer-smartphone Bluetooth connections and real-time analyses including interactive algorithms to trigger participants in phases of low and high PA (for a detailed discussion, see [148]). Such challenges have been especially encountered in studies with older or inactive samples [126, 154–157]. Further measures to improve participant compliance in daily-life studies on PB-AWB are critical, such as study personnel increasing participant motivation (for a detailed discussion, see Reichert et al. [48]). Most reviewed studies (41/66) collected data over 7 or less days. While in general designing an AA study requires an appropriate assessment duration to collect sufficient data for the analysis of momentary within-subject processes, both the person level (i.e., the number of participants) and the prompt level (i.e., e-diary entries) data are critical to statistical power but of different importance depending on the analysis planned (e.g., at the same power and alpha level, within-subject direct effects require much less data compared to cross-level interaction effects) [158]. However, an in this context crucial but often unattended aspect is that the sampling frequency must fit the process of interest to produce univocal results [28, 48, 159], which often conflicts with long assessment durations, for example, high-frequency e-diary assessments such as every 15 min across 10 h a day (which equals 40 prompts a day) to appropriately capture AWB within-person variation across more than 1 day will obviously pose a huge burden on participants and lead to compliance issues [48, 159]. Against this background, to capture both PB-AWB short-term responses and long-term effects, we expect that future PB-AWB studies may be designed to collect data over longer assessment durations yet concurrently draw from high-frequency assessments, which is possible via so-called measurement burst designs combining sparse and intense sampling phases [160, 161]. Following standard procedures in AA, most of the studies reviewed conducted two-level multilevel modeling. Against the background of limitations to these models which we detail in Sect. 4.6, we expect that in future, the field will draw from advanced statistical approaches to unravel the timing of effects and PB compositions in detail. A review published by Ruissen and colleagues [84] provides a comprehensive overview of some dynamic measurement and modeling approaches applicable to AA-studies in the PB-AWB field [85].

### Limitations

This review entails many strengths, but some aspects merit further discussion. First, in our work, we searched three databases, and thus it cannot be assured that some appropriate literature on PB-AWB associations has not been inadvertently missed. Moreover, we did not include unpublished work, or grey literature. We acknowledge that the exclusion of grey literature may represent a limitation of our review since this may have resulted in our literature overview not being fully comprehensive. However, the inclusion of grey literature, where quality standards are not uniformly assessed, into literature reviews is under debate [51]. Mixing peer-reviewed with non-peer-reviewed studies could introduce bias into the interpretation of results [51]. However, since we searched the most comprehensive and recognized databases, we do not expect the findings of our review to be critically biased by the search strategy. Second, the modified QA employed had been custom-developed, and should be further validated. Still, we would like to emphasize that our QA follows high standards, given that it was guided by and includes items of already existing and validated QAs [44, 53, 54] (see ESM 8). As such, we assume that we have covered relevant QA aspects. Of note, following established recommendations [45], our QA is not primarily intended to reflect the hierarchical quality of studies, for example, via between-study rankings, but rather to detect potential flaws and thus better reflect the internal validity of studies. Beyond a risk of bias rating, our modified QA was mainly guided by the concept to rate whether studies provided sufficient information for future studies to replicate the investigations conducted. Third, we did not include intervention studies, but rather only observational real-life investigations. This precludes causal conclusions and direct recommendations for interventions. However, since there are currently only very few intervention studies including daily life methods (e.g., combining experimental manipulation and ecological validity), this proposal should be substantiated by future reviews. Fourth, the studies reviewed did not report uniform standardized effect sizes. Critically, reliable effect sizes in intensive longitudinal data analyses must be informed by a wealth of statistical parameters (e.g., variances on the different analyses levels [158]). Therefore, it was not possible to conduct a meta-analysis solely with the information provided in the papers. However, to give hints on the meaningfulness of effects found in the studies reviewed, we provided readers with a summary of practical effect sizes reported. Future work on PB-AWB associations should include statistical parameters to enable uniform standardized effect size, or alternatively, researchers may aim for conducting individual participant data meta-analysis in a future open research framework. Fifth, in the studies reviewed, a large proportion of convenience samples were investigated (e.g., students or university employees), limiting generalizability. Sixth, most the AA studies aggregated PA across distinct time frames prior to and/or following the e-diary prompts, a parameterization we described as “aggregated time frames.” For example, in several studies reviewed, aggregated time frame equaled 15 min before and/or after the e-diary prompts. Accordingly, in these studies, researchers investigated associations of PA occurring 15 min before and/or after the e-diary rating with AWB. Importantly, this does not give any information about the particular composition of PA conducted within the aggregated time frame. More precisely and drawing from a prominent example derived from the studies reviewed, if parameterizing PA as minutes of MVPA within the 15 min before an AWB rating, a value of 8 min MVPA may result from a person running 8 min in a row across 15 min, but also from this person achieving 8 MVPA min in total across 15 min through four interspersed MVPA bouts of 2 min each. If data entail values of 15 min of MVPA, this may even stem from exercising sessions by far exceeding 15 min. Of note, studies under investigation differed in their operationalizations of average PA within the aggregated time frames, for example, some used the parameters time spent in LPA, MVPA, or SB, while others were interested in metric operationalizations of PA (ESM 9). Moreover, the underlying parameterization does not give information on the type of PA, nor it does allow for a precise investigation of the timing of effects (e.g., at which time lag after being physically active is AWB being affected most). Accordingly, this way of parameterization of PA does not allow direct inferences on the PA composition provoking potential AWB effects. While many studies differentiated their analyses by PA intensity (e.g., LPA, MVPA), this challenge is particularly salient to the PA duration and timing of effects domains against the background of the aim of the present work to summarize existing studies. Hence, to receive more information on the underlying physical activity composition provoking potential AWB effects, the parameterization and related statistical modelling is a critical challenge to the field to be tackled in the upcoming years. Fortunately, in a comprehensive work, Ruissen and colleagues reviewed [84] custom-developed and applied methods (i.e., Bayesian hierarchical continuous-time structural equation models) for overcoming these limitations [85]. Seventh, the QA of methods used to study PB-AWB associations revealed large heterogeneity, which limits interpretability of the results (for an in-depth discussion, see Sect. 4.6). Therefore, researchers may streamline their methodological approaches and engage in a more detailed reporting of methods used (e.g., accelerometry data preprocessing procedures). Eighth, only a small proportion of the studies reviewed conducted a (post hoc) power analysis to estimate the appropriate sample size or did not report it. Therefore, some of the results may be underpowered which may have led to type-2 error inflation in our review.

## Conclusions, Practical Recommendations, and Future Directions

Our search revealed that the number of daily-life studies on PB-AWB has increased rapidly. In sum, the reviewed evidence on PB-AWB associations under ecological valid conditions is heterogeneous, that is, the direction and strength of relationships is ambiguous across studies. Therefore, one might be tempted to speculate that PB and AWB are not related to each other in each and every situation and in all humans, but are dependent on contextual factors (such as time, situational, and social context, weather conditions), PB and AWB components (such as PB duration and intensity; emotions, affect, mood) and biological determinants.

Amalgamation of the findings revealed that PB intensity barely revealed any systematic effect on everyday life AWB and vice versa, while the review of other PB characteristics (such as PA duration and type) is hampered by methodological limitations in the field that are currently being tackled. However, in general, most studies investigated primarily incidental PB, and studies found positive AWB effects even for low intensities; these findings should be followed up by novel AA approaches to research PB characteristics, and they can fuel the discussion about whether the World Health Organization notion “every move counts” [1] may be extended to everyday-life AWB. Similarly, AWB characteristics (such as emotions, affective states, mood) do not fully explain variance of PB-AWB associations, but, importantly, PB relations with subjective energy were largely homogenous across studies. This points to a dominant role of feelings of energy, a reasonable finding against the evidence from mental health studies and previous meta-analyses on positive activated affect. A high relative amount of significant investigated PB-AWB relationships were found in people susceptible to mental disorders compared to other populations. We found a large heterogeneity of methods applied to study PB-AWB associations, which further complicates scrutiny of real-life evidence on PB-AWB associations. While overall the quality of studies reviewed was rated moderate to high, there is considerable room for improvements. In particular, PB measurement via accelerometry is considered the gold standard and was set as an inclusion criterion in this review, but the devices used and procedures applied show large variability. While repeated AWB assessment in real-time is at least in part conducted via questionnaires validated for AA purposes, barely any study of those reviewed used questionnaires specified for the individual everyday-life PB-AWB association purpose. AA sampling procedures were not always tailored to the PB-AWB process of interest, and compliance reporting was in part insufficient, especially for accelerometry. Therefore, over and above method improvements, streamlining of methodological procedures to investigate PB-AWB association, and especially more transparent reporting of methods, are critical for future investigations in the field.

Since the direction and strength of the PB-AWB associations vary across studies, this suggests that the association is not universally consistent but may amongst other influences (e.g., biological determinants) also depend on daily-life contextual factors. Contextual influences are known to be key determinants of human behavior and feelings [162]. In contrast to laboratory studies, real-life investigations offer the possibility of studying these moderation effects. For example, environmental factors (such as outdoor vs. indoor settings, nature vs. built environments, as well as air and noise pollution) are shown to influence both PB and AWB [162–165]. Accordingly, such environmental influences may also play a potential moderating role on the PB-AWB association, and their consideration should be a central aspect of future AA studies. Supporting this hypothesis, a study showed that PA being performed outdoors revealed higher affective benefits compared to indoor PA [135], a finding consistent with other studies [162, 166–170]. Another example of contextual influences is PB-AWB moderation effects by weather; poor weather conditions have been associated with lower levels of MVPA [78], while higher temperatures were linked to increased PA levels [171]. Moreover, situational contexts, such as work versus leisure environments, have also been found to influence the PB-AWB association [172]; for example, the frequency and intensity of sedentary breaks have a more pronounced effect on energetic arousal when individuals are at home compared to being at work [78, 86]. Furthermore, social contexts should be a focal point in future research, since influences on the PB-AWB real-life association are highly conceivable. For example, engaging in PA in social settings has been found to enhance AWB [135], to increase the duration of activities [164], and a study demonstrated influences of partner support on the interaction of SB and AWB [77]. These moderation effects could also extend to the complex contextual interactions within families and among friends [79, 132, 165, 167, 173–175]. In conclusion, contextual factors are integral to our understanding of PB-AWB associations, and we argue that investigating these interactions in future real-life settings is essential for gaining comprehensive insights. The PB-AWB association is highly relevant to both physical and mental health in humans as outlined above. This puts forward highly promising future follow-up research questions, which can be critically informed by this review. First, it emphasizes the ongoing need to tackle the issue of causality in more depth. For example, the reviewed studies show PB-AWB correlations in both temporal directions, which leads to the assumption of a circular relationship [85]. Second, the issue arises how the PB-AWB association can be exploited to proceed toward precision medicine approaches. For example, the specificity of PB-AWB associations for distinct populations found in this review can set the basis to build “acute dynamic process phenotypes” for the prediction of prospective health behavior [48, 176, 177]. Third, this includes the question of how the extracted knowledge can shape and refine existing health behavior theories and even promote novel health behavior models. For example, the strong PB-AWB link with feelings of energy in both directions found in the present synthesis of everyday-life studies perfectly fits with innovative health behavior theories hypothesizing PB engagement to be mediated by cravings for PA [13] and the affective-reflective theory [178].

To tackle these follow-up questions, future research can draw from methodological advancements. For example, sophisticated Granger causality [179] approaches have been suggested for intensive longitudinal data modeling [180, 181], and in future, experimental manipulations in everyday life (e.g., [182]) can help to approach issues of PB-AWB causality. Second, technological advancements such as high-resolution smartphone sensing (e.g., application-use, calls and text message tracking, voice pitch [150, 183–186]), physiology tracking in real-life (such as skin, heart rate), and combinations with laboratory testing (such as neuroimaging, intestinal microbes [19]; multiparametric sensor fusion [187, 188]), can be exploited to proceed towards precision medicine approaches. Third, meta-analytic strategies with individual participant data can scrutinize evidence to shape and refine existing health behavior theories and to inform novel health behavior models. Together, these insights will help to promote and develop (mobile) interventions for prevention and therapy of human physical and mental health.

### Supplementary Information

Below is the link to the electronic supplementary material.

Distribution of reviewed studies (DOCX 29 KB)

PRISMA checklist (DOCX 19 KB)

Comprehensive search term (DOCX 13 KB)

Adapted Quality Assessment Tool (DOCX 21 KB)

Detailed Quality Assessment (DOCX 45 KB)

Affective well-being categories (XLSX 48 KB)

Affective well-being and physical behavior (further items; a) (JPG 260 KB)

Affective well-being and physical behavior (further items; b) (JPG 226 KB)

Modified quality assessment development (DOCX 14 KB)

Average physical activity across the aggregated time frames (DOCX 35 KB)

## Declarations

### Availability of data and material

The data are available from the authors on request.

## References

[1] World Health Organization. Global status report on physical activity 2022. World Health Organization. 2022. https://iris.who.int/bitstream/handle/10665/363607/9789240059153-eng.pdf?sequence=1. Accessed 15 Apr 2023.

[2] Warburton DER, Nicol CW, Bredin SSD (2006). Health benefits of physical activity: the evidence. CMAJ.

[3] Bull FC, Al-Ansari SS, Biddle SJH, Borodulin K, Buman MP, Cardon G (2020). World Health Organization 2020 guidelines on physical activity and sedentary behaviour. Br J Sports Med.

[4] Owen N, Healy GN, Matthews CE, Dunstan DW (2010). Too much sitting: the population health science of sedentary behavior. Exerc Sport Sci Rev.

[5] Dunton GF (2018). Sustaining health-protective behaviors such as physical activity and healthy eating. JAMA.

[6] Pearce M, Garcia L, Abbas A, Strain T, Schuch FB, Golubic R (2022). Association between physical activity and risk of depression: a systematic review and meta-analysis. JAMA Psychiat.

[7] Singh B, Olds T, Curtis R, Dumuid D, Virgara R, Watson A (2023). Effectiveness of physical activity interventions for improving depression, anxiety and distress: an overview of systematic reviews. Br J Sports Med.

[8] Williams DM, Rhodes RE, Conner M (2018). Affective determinants of health behavior.

[9] Ekkekakis P (2003). Pleasure and displeasure from the body: perspectives from exercise. Cogn Emot.

[10] Evans JSBT, Stanovich KE (2013). Dual-process theories of higher cognition: advancing the debate. Perspect Psychol Sci.

[11] Williams DM, Rhodes RE, Conner MT (2019). Conceptualizing and intervening on affective determinants of health behaviour. Psychol Health.

[12] Brand R, Schweizer G (2015). Going to the gym or to the movies?: situated decisions as a functional link connecting automatic and reflective evaluations of exercise with exercising behavior. J SPORT Exerc Psychol.

[13] Stults-Kolehmainen MA, Blacutt M, Bartholomew JB, Gilson TA, Ash GI, McKee PC (2020). Motivation states for physical activity and sedentary behavior: desire, urge, wanting, and craving. Front Psychol.

[14] Stephens T (1988). Physical activity and mental health in the United States and Canada: evidence from four population surveys. Prev Med.

[15] Goodwin RD (2003). Association between physical activity and mental disorders among adults in the United States. Prev Med.

[16] Stanton R, Happell B (2014). Exercise for mental illness: a systematic review of inpatient studies. Int J Ment Health Nurs.

[17] National Collaborating Centre for Mental Health (UK). Depression in adults with a chronic physical health problem: treatment and management (NICE Clinical Guideline 91). British Psychological Society. 2010. https://www.ncbi.nlm.nih.gov/books/NBK82916/. 22259826

[18] Merikangas KR, Swendsen J, Hickie IB, Cui L, Shou H, Merikangas AK (2019). Real-time mobile monitoring of the dynamic associations among motor activity, energy, mood, and sleep in adults with bipolar disorder. JAMA Psychiat.

[19] Reichert M, Braun U, Gan G, Reinhard I, Giurgiu M, Ma R (2020). A neural mechanism for affective well-being: subgenual cingulate cortex mediates real-life effects of nonexercise activity on energy. Sci Adv.

[20] Biddle S (2016). Physical activity and mental health: evidence is growing. World Psychiatry.

[21] Kelly P, Williamson C, Niven AG, Hunter R, Mutrie N, Richards J (2018). Walking on sunshine: scoping review of the evidence for walking and mental health. Br J Sports Med.

[22] White RL, Babic MJ, Parker PD, Lubans DR, Astell-Burt T, Lonsdale C (2017). Domain-specific physical activity and mental health: a meta-analysis. Am J Prev Med.

[23] Reed J, Buck S (2009). The effect of regular aerobic exercise on positive-activated affect: a meta-analysis. Psychol Sport Exerc.

[24] Reed J, Ones DS (2006). The effect of acute aerobic exercise on positive activated affect: a meta-analysis. Psychol Sport Exerc.

[25] Fahrenberg J, Myrtek M, Pawlik K, Perrez M (2007). Ambulatory assessment—monitoring behavior in daily life settings. Eur J Psychol Assess.

[26] SAA—society for ambulatory assessment. 17.12.2020. https://ambulatory-assessment.org/. Accessed 17 Dec 2023

[27] Ebner-Priemer UW, Trull TJ (2009). Ambulatory assessment: an innovative and promising approach for clinical psychology. Eur Psychol.

[28] Trull TJ, Ebner-Priemer U (2013). Ambulatory assessment. Annu Rev Clin Psychol.

[29] Mehl MR, Conner TS, Csikszentmihalyi M (2014). Handbook of research methods for studying daily life.

[30] Thomas DL, Diener E (1990). Memory accuracy in the recall of emotions. J Pers Soc Psychol.

[31] Shiffman S, Stone AA, Hufford MR (2008). Ecological momentary assessment. Annu Rev Clin Psychol.

[32] Caspersen CJ, Powell KE, Christenson GM (1985). Physical activity, exercise, and physical fitness: definitions and distinctions for health-related research. Public Health Rep.

[33] Tremblay MS, Aubert S, Barnes JD, Saunders TJ, Carson V, Latimer-Cheung AE (2017). Sedentary behavior research network (SBRN)—terminology consensus project process and outcome. Int J Behav Nutr Phys Act.

[34] Lischetzke T, Eid M (2006). Why extraverts are happier than introverts: the role of mood regulation. J Pers.

[35] Russell JA (1980). A circumplex model of affect. J Pers Soc Psychol.

[36] Russell JA. Cross-cultural similarities and differences in affective processing and expression. In: Jeon M, editor. Emotions and affect in human factors and human-computer interaction. London: Elsevier Academic Press; 2017. pp. 123–141. 10.1016/B978-0-12-801851-4.00004-5

[37] Watson D, Clark LA, Tellegen A (1988). Development and validation of brief measures of positive and negative affect: the PANAS scales. J Pers Soc Psychol.

[38] Wilhelm P, Schoebi D (2007). Assessing mood in daily life. Eur J Psychol Assess.

[39] Ekkekakis P (2013). The measurement of affect, mood, and emotion: a guide for health-behavioral research.

[40] Barrett LF, Lewis M, Haviland-Jones JM (2018). Handbook of emotions.

[41] Liao Y, Shonkoff ET, Dunton GF (2015). The acute relationships between affect, physical feeling states, and physical activity in daily life: a review of current evidence. Front psychol.

[42] Bourke M, Hilland TA, Craike M (2021). A systematic review of the within-person association between physical activity and affect in children’s and adolescents’ daily lives. Psychol Sport Exerc.

[43] Bolger N, Laurenceau J-P (2013). Intensive longitudinal methods: an introduction to diary and experience sampling research.

[44] National Heart, Lung, and Blood Institute. Study quality assessment tools. 2020. https://www.nhlbi.nih.gov/health-topics/study-quality-assessment-tools. Accessed 4 Nov 2023.

[45] Page MJ, Moher D, Bossuyt PM, Boutron I, Hoffmann TC, Mulrow CD (2021). PRISMA 2020 explanation and elaboration: updated guidance and exemplars for reporting systematic reviews. BMJ-Br Med J..

[46] Page MJ, McKenzie JE, Bossuyt PM, Boutron I, Hoffmann TC, Mulrow CD (2021). The PRISMA 2020 statement: an updated guideline for reporting systematic reviews. BMJ.

[47] Burchartz A, Anedda B, Auerswald T, Giurgiu M, Hill H, Ketelhut S (2020). Assessing physical behavior through accelerometry—State of the science, best practices and future directions. Psychol Sport Exerc.

[48] Reichert M, Giurgiu M, Koch ED, Wieland LM, Lautenbach S, Neubauer AB (2020). Ambulatory assessment for physical activity research: State of the science, best practices and future directions. Psychol Sport Exerc.

[49] Egloff B, Tausch A, Kohlmann C-W, Krohne HW (1995). Relationships between time of day, day of the week, and positive mood: exploring the role of the mood measure. Motiv Emot.

[50] Paez A (2017). Gray literature: an important resource in systematic reviews. J Evid-Based Med..

[51] Mahood Q, Van Eerd D, Irvin E (2014). Searching for grey literature for systematic reviews: challenges and benefits. Res Synth Methods.

[52] Stevens ML, Gupta N, Inan Eroglu E, Crowley PJ, Eroglu B, Bauman A (2020). Thigh-worn accelerometry for measuring movement and posture across the 24-hour cycle: a scoping review and expert statement. BMJ Open Sport Exerc Med.

[53] Liao Y, Skelton K, Dunton G, Bruening M (2016). A systematic review of methods and procedures used in ecological momentary assessments of diet and physical activity research in youth: an adapted STROBE checklist for reporting EMA studies (CREMAS). J Med Internet Res.

[54] Trull TJ, Ebner-Priemer UW (2020). Ambulatory assessment in psychopathology research: a review of recommended reporting guidelines and current practices. J Abnorm Psychol.

[55] Thomas BH, Ciliska D, Dobbins M, Micucci S (2004). A process for systematically reviewing the literature: providing the research evidence for public health nursing interventions. Worldv Evid Based Nurs..

[56] Koo TK, Li MY (2016). A guideline of selecting and reporting intraclass correlation coefficients for reliability research. J Chiropr Med.

[57] Shannon CE (1949). Communication in the presence of noise. Proc IRE.

[58] Kang M, Rowe DA (2015). Issues and challenges in sedentary behavior measurement. Meas Phys Educ Exerc Sci.

[59] Rowlands AV (2007). Accelerometer assessment of physical activity in children: an update. Pediatr Exerc Sci.

[60] Chen KY, Bassett DR (2005). The technology of accelerometry-based activity monitors: current and future. Med Sci Sports Exerc.

[61] ActiGraph LLC. https://www.actigraph.nl/en/. Accessed 24 Feb 2023.

[62] movisens GmbH. https://www.movisens.com/en/. Accessed 24 Feb 2023.

[63] Becker Meditec. varioport-e. https://bisigma.de/pdf/vario_e-kurzinfo.pdf. Accessed 24 Feb 2023.

[64] Fitbit. https://www.fitbit.com/global/us/home. Accessed 24 Feb 2023.

[65] Steyer R, Schwenkmezger P, Notz P, Eid M (1997). Der Mehrdimensionale Befindlichkeitsfragebogen (MDBF).

[66] Ebesutani C, Regan J, Smith A, Reise S, Higa-McMillan C, Chorpita BF (2012). The 10-item positive and negative affect schedule for children, child and parent shortened versions: application of item response theory for more efficient assessment. J Psychopathol Behav Assess.

[67] Krohne HW, Egloff B, Kohlmann C-W, Tausch A (1996). Untersuchungen mit einer deutschen Form der Positive and Negative Affect Schedule (PANAS): [Investigations with a German version of the positive and negative affect schedule (PANAS)]. Diagnostica.

[68] Mackinnon A, Jorm AF, Christensen H, Korten AE, Jacomb PA, Rodgers B (1999). A short form of the Positive and Negative Affect Schedule: evaluation of factorial validity and invariance across demographic variables in a community sample. Personal Individ Differ.

[69] Thompson ER (2007). Development and validation of an internationally reliable short-form of the positive and negative affect schedule (PANAS). J Cross-Cult Psychol..

[70] Posner J, Russell JA, Peterson BS (2005). The circumplex model of affect: an integrative approach to affective neuroscience, cognitive development, and psychopathology. Dev Psychopathol.

[71] McNair DM, Lorr M, Droppleman LF (1971). Manual for the profile of mood states.

[72] Fukui I (1997). The depression and anxiety mood scale (DAMS): scale development and validation. Jpn J Behav Ther.

[73] Rhodes RE, Hausenblas HA, Rebar AL (2023). Psychology of physical activity and sedentary behavior.

[74] Borg DN, Barnett AG, Caldwell AR, White NM, Stewart IB (2023). The bias for statistical significance in sport and exercise medicine. J Sci Med Sport.

[75] Büttner F, Toomey E, McClean S, Roe M, Delahunt E (2020). Are questionable research practices facilitating new discoveries in sport and exercise medicine? The proportion of supported hypotheses is implausibly high. Br J Sports Med.

[76] Cumming G. The New Statistics (2014). Why and how. Psychol Sci.

[77] Zhaoyang R, Martire LM (2019). Daily sedentary behavior predicts pain and affect in knee arthritis. Ann Behav Med.

[78] Zenk SN, Horoi I, Jones KK, Finnegan L, Corte C, Riley B (2017). Environmental and personal correlates of physical activity and sedentary behavior in African American women: an ecological momentary assessment study. WOMEN Health.

[79] Yang C-H, Huh J, Mason TB, Belcher BR, Kanning M, Dunton GF (2020). Mother-child dyadic influences of affect on everyday movement behaviors: evidence from an ecological momentary assessment study. Int J Behav Nutr Phys Act.

[80] Koch ED, Freitag CM, Mayer JS, Medda J, Reif A, Grimm O (2022). The dynamical association between physical activity and affect in the daily life of individuals with ADHD. Eur Neuropsychopharmacol.

[81] Cushing CC, Mitchell TB, Bejarano CM, Walters RW, Crick CJ, Noser AE. Bidirectional associations between psychological states and physical activity in adolescents: a mHealth pilot study. J Pediatr Psychol. 2017;jsw099. 10.1093/jpepsy/jsw099 28131985

[82] Stevenson BL, Kunicki ZJ, Brick L, Blevins CE, Stein M, Abrantes AM (2022). Using ecological momentary assessments and fitbit data to examine daily associations between physical activity, affect and alcohol cravings in patients with alcohol use disorder. Int J Behav Med.

[83] Schwerdtfeger A, Eberhardt R, Chmitorz A, Schaller E (2010). Momentary affect predicts bodily movement in daily life: an ambulatory monitoring study. J Sport Exerc Psychol.

[84] Ruissen GR, Zumbo BD, Rhodes RE, Puterman E, Beauchamp MR (2022). Analysis of dynamic psychological processes to understand and promote physical activity behaviour using intensive longitudinal methods: a primer. Health Psychol Rev.

[85] Ruissen GR, Beauchamp MR, Puterman E, Zumbo BD, Rhodes RE, Hives BA (2022). Continuous-time modeling of the bidirectional relationship between incidental affect and physical activity. Ann Behav Med.

[86] Giurgiu M, Koch ED, Plotnikoff RC, Ebner-Priemer UW, Reichert M (2020). Breaking up sedentary behavior optimally to enhance mood. Med Sci Sports Exerc.

[87] Kanning M, Schoebi D (2016). Momentary affective states are associated with momentary volume, prospective trends, and fluctuation of daily physical activity. Front Psychol.

[88] Reichert M, Tost H, Reinhard I, Schlotz W, Zipf A, Salize H-J (2017). Exercise versus nonexercise activity: E-diaries unravel distinct effects on mood. Med Sci Sports Exerc.

[89] Giurgiu M, Ebner-Priemer UW, Dumuid D (2022). Compositional insights on the association between physical activity and sedentary behavior on momentary mood in daily life. Psychol Sport Exerc.

[90] Giurgiu M, Koch ED, Ottenbacher J, Plotnikoff RC, Ebner-Priemer UW, Reichert M (2019). Sedentary behavior in everyday life relates negatively to mood: an ambulatory assessment study. Scand J Med Sci Sports.

[91] Koch ED, Tost H, Braun U, Gan G, Giurgiu M, Reinhard I (2020). Relationships between incidental physical activity, exercise, and sports with subsequent mood in adolescents. Scand J Med Sci Sports.

[92] Reichert M, Tost H, Reinhard I, Zipf A, Salize H-J, Meyer-Lindenberg A, Ebner-Priemer UW (2016). Within-subject associations between mood dimensions and non-exercise activity: an ambulatory assessment approach using repeated real-time and objective data. Front Psychol.

[93] Koch ED, Tost H, Braun U, Gan G, Giurgiu M, Reinhard I (2018). Mood dimensions show distinct within-subject associations with non-exercise activity in adolescents: an ambulatory assessment study. Front Psychol.

[94] Giurgiu M, Plotnikoff RC, Nigg CR, Koch ED, Ebner-Priemer UW, Reichert M (2020). Momentary mood predicts upcoming real-life sedentary behavior. Scand J Med Sci Sports.

[95] Madden DR, Nok Lam C, Redline B, Dzubur E, Rhoades H, Intille SS (2020). Real-time data collection to examine relations between physical activity and affect in adults with mental illness. J Sport Exerc Psychol.

[96] Tremblay MS, Esliger DW, Tremblay A, Colley R (2007). Incidental movement, lifestyle-embedded activity and sleep: new frontiers in physical activity assessment. Can J Public Health.

[97] Ainsworth BE, Haskell WL, Herrmann SD, Meckes N, Bassett DR, Tudor-Locke C (2011). 2011 Compendium of physical activities: a second update of codes and MET values. Med Sci Sports Exerc.

[98] Buecker S, Simacek T, Ingwersen B, Terwiel S, Simonsmeier BA (2021). Physical activity and subjective well-being in healthy individuals: a meta-analytic review. Health Psychol Rev.

[99] Fahrenberg J, Myrtek M (1996). Ambulatory assessment: computer-assisted psychological and psychophysiological methods in monitoring and field studies.

[100] Ekkekakis P, Hall E, Petruzzello S (2005). Variation and homogeneity in affective responses to physical activity of varying intensities: an alternative perspective on dose-response based on evolutionary considerations. J Sports Sci.

[101] Ojanen M (1994). Can the true effects of exercise on psychological variables be separated from placebo effects. Int J Sport Psychol.

[102] Ekkekakis P, Petruzzello SJ (1999). Acute aerobic exercise and affect: current status, problems and prospects regarding dose-response. Sports Med.

[103] Durstine JL, Grandjean PW, Davis PG, Ferguson MA, Alderson NL, DuBose KD (2001). Blood lipid and lipoprotein adaptations to exercise: a quantitative analysis. Sports Med.

[104] Basso JC, Suzuki WA (2017). The effects of acute exercise on mood, cognition, neurophysiology, and neurochemical pathways: a review. Brain Plast Amst Neth..

[105] Stevens CJ, Baldwin AS, Bryan AD, Conner M, Rhodes RE, Williams DM (2020). Affective determinants of physical activity: a conceptual framework and narrative review. Front Psychol.

[106] Pintar JA, Robertson RJ, Kriska AM, Nagle E, Goss FL (2006). The influence of fitness and body weight on preferred exercise intensity. Med Sci Sports Exerc.

[107] Ekkekakis P, Hall EE, Petruzzello SJ (2004). Practical markers of the transition from aerobic to anaerobic metabolism during exercise: rationale and a case for affect-based exercise prescription. Prev Med.

[108] Ekkekakis P, Lind E (2006). Exercise does not feel the same when you are overweight: the impact of self-selected and imposed intensity on affect and exertion. Int J Obes.

[109] Williams DM, Dunsiger S, Miranda R, Gwaltney CJ, Emerson JA, Monti PM (2015). Recommending self-paced exercise among overweight and obese adults: a randomized pilot study. Ann Behav Med Publ Soc Behav Med..

[110] Parfitt G, Rose EA, Burgess WM (2006). The psychological and physiological responses of sedentary individuals to prescribed and preferred intensity exercise. Br J Health Psychol.

[111] Rhodes RE, Kates A (2015). Can the affective response to exercise predict future motives and physical activity behavior? A systematic review of published evidence. Ann Behav Med.

[112] Jeckel S, Sudeck G (2016). Physical activity and affective well-being in everyday life: comparing sport activities and daily physical activities regarding acute and sustainable associations. Z Für Gesundheitspsychol..

[113] Berger BG, Motl RW (2000). Exercise and mood: a selective review and synthesis of research employing the profile of mood states. J Appl Sport Psychol.

[114] Steptoe A, Cox S (1988). Acute effects of aerobic exercise on mood. Health Psychol.

[115] Rajab AS, Crane DE, Middleton LE, Robertson AD, Hampson M, MacIntosh BJ (2014). A single session of exercise increases connectivity in sensorimotor-related brain networks: a resting-state fMRI study in young healthy adults. Front Hum Neurosci.

[116] Byun K, Hyodo K, Suwabe K, Ochi G, Sakairi Y, Kato M (2014). Positive effect of acute mild exercise on executive function via arousal-related prefrontal activations: an fNIRS study. Neuroimage.

[117] Goldfarb AH, Hatfield BD, Armstrong D, Potts J (1990). Plasma beta-endorphin concentration: response to intensity and duration of exercise. Med Sci Sports Exerc.

[118] Chan JSY, Liu G, Liang D, Deng K, Wu J, Yan JH. Special issue—therapeutic benefits of physical activity for mood: a systematic review on the effects of exercise intensity, duration, and modality. J Psychol. 2019;153:102–25. 10.1080/00223980.2018.1470487. 10.1080/00223980.2018.1470487 30321106

[119] Russell JA (2003). Core affect and the psychological construction of emotion. Psychol Rev.

[120] Thayer RE (1996). The origin of everyday moods: Managing energy, tension, and stress.

[121] Brähler E, Mühlan H, Albani C, Schmidt S (2007). Teststatistische Prüfung und Normierung der deutschen Versionen des EUROHIS-QOL Lebensqualität-Index und des WHO-5 Wohlbefindens-Index. Diagnostica.

[122] Topp CW, Østergaard SD, Søndergaard S, Bech P (2015). The WHO-5 Well-Being Index: a systematic review of the literature. Psychother Psychosom.

[123] Ekkekakis P, Zenko Z, Vazou S (2021). Do you find exercise pleasant or unpleasant? The Affective Exercise Experiences (AFFEXX) questionnaire. Psychol SPORT Exerc.

[124] Kerrigan SG, Schumacher L, Manasse SM, Loyka C, Butryn ML, Forman EM (2020). The association between negative affect and physical activity among adults in a behavioral weight loss treatment. Psychol Sport Exerc.

[125] Kim J, Nakamura T, Kikuchi H, Sasaki T, Yamamoto Y (2013). Co-variation of depressive mood and locomotor dynamics evaluated by ecological momentary assessment in healthy humans. PLoS ONE.

[126] Kim J, Nakamura T, Kikuchi H, Yoshiuchi K, Sasaki T, Yamamoto Y (2015). Covariation of depressive mood and spontaneous physical activity in major depressive disorder: toward continuous monitoring of depressive mood. IEEE J Biomed Health Inform.

[127] Langguth N, Schmid J, Gawrilow C, Stadler G (2016). Within-person link between depressed affect and moderate-to-vigorous physical activity in adolescence: an intensive longitudinal approach. Appl Psychol-Health Well Being..

[128] Ekkekakis P, Hartman ME, Ladwig MA. Affective Responses to Exercise. In: Tenenbaum G, Eklund RC, Boiangin N, editors. Handbook of sport psychology. Hoboken: Wiley; 2020. pp. 231–253. 10.1002/9781119568124.ch12.

[129] Cushing CC, Bejarano CM, Mitchell TB, Noser AE, Crick CJ (2018). Individual differences in negative affectivity and physical activity in adolescents: an ecological momentary assessment study. J Child Fam Stud.

[130] Dunton GF, Huh J, Leventhal AM, Riggs N, Hedeker D, Spruijt-Metz D (2014). Momentary assessment of affect, physical feeling states, and physical activity in children. Health Psychol.

[131] Smith KE, Haedt-Matt A, Mason TB, Wang S, Yang C-H, Unick JL (2020). Associations between naturalistically assessed physical activity patterns, affect, and eating in youth with overweight and obesity. J Behav Med.

[132] Wen CKF, Liao Y, Maher JP, Huh J, Belcher BR, Dzubur E (2018). Relationships among affective states, physical activity, and sedentary behavior in children: moderation by perceived stress. Health Psychol.

[133] Molenaar PCM (2004). A manifesto on psychology as idiographic science: bringing the person back into scientific psychology, this time forever. Meas Interdiscip Res Perspect.

[134] Reichert M, Brüßler S, Reinhard I, Braun U, Giurgiu M, Hoell A (2022). The association of stress and physical activity: Mind the ecological fallacy. Ger J Exerc Sport Res.

[135] Cabrita M, Lousberg R, Tabak M, Hermens HJ, Vollenbroek-Hutten MMR (2017). An exploratory study on the impact of daily activities on the pleasure and physical activity of older adults. Eur Rev Aging Phys Act..

[136] Wilhelm LO, Pauly T, Ashe MC, Hoppmann CA (2021). Daily physical activity in older age. GeroPsych.

[137] Troiano RP, Berrigan D, Dodd KW, Mâsse LC, Tilert T, McDowell M (2008). Physical activity in the United States measured by accelerometer. Med Sci Sports Exerc.

[138] Freedson P, Bowles HR, Troiano R, Haskell W (2012). Assessment of physical activity using wearable monitors: recommendations for monitor calibration and use in the field. Med Sci Sports Exerc.

[139] Strath SJ, Kaminsky LA, Ainsworth BE, Ekelund U, Freedson PS, Gary RA (2013). Guide to the assessment of physical activity: Clinical and research applications: a scientific statement from the American Heart Association. Circulation.

[140] Migueles JH, Cadenas-Sanchez C, Ekelund U, Delisle Nyström C, Mora-Gonzalez J, Löf M (2017). Accelerometer data collection and processing criteria to assess physical activity and other outcomes: a systematic review and practical considerations. Sports Med.

[141] Trost SG, McIver KL, Pate RR (2005). Conducting accelerometer-based activity assessments in field-based research. Med Sci Sports Exerc.

[142] Edwardson CL, Gorely T (2010). Epoch length and its effect on physical activity intensity. Med Sci Sports Exerc.

[143] Altenburg TM, Wang X, van Ekris E, Andersen LB, Møller NC, Wedderkopp N (2021). The consequences of using different epoch lengths on the classification of accelerometer based sedentary behaviour and physical activity. PLoS ONE.

[144] Vähä-Ypyä H, Vasankari T, Husu P, Mänttäri A, Vuorimaa T, Suni J (2015). Validation of cut-points for evaluating the intensity of physical activity with accelerometry-based mean amplitude deviation (MAD). PLoS ONE.

[145] Trost SG (2020). Population-level physical activity surveillance in young people: are accelerometer-based measures ready for prime time?. Int J Behav Nutr Phys Act.

[146] Keadle SK, Lyden KA, Strath SJ, Staudenmayer JW, Freedson PS (2019). A framework to evaluate devices that assess physical behavior. Exerc Sport Sci Rev.

[147] Pedišić Ž, Bauman A (2015). Accelerometer-based measures in physical activity surveillance: current practices and issues. Br J Sports Med.

[148] Ebner-Priemer UW, Koudela S, Mutz G, Kanning M (2013). Interactive multimodal ambulatory monitoring to investigate the association between physical activity and affect. Front Psychol.

[149] Degroote L, DeSmet A, Bourdeaudhuij I, van Dyck D, Crombez G (2020). Content validity and methodological considerations in ecological momentary assessment studies on physical activity and sedentary behaviour: a systematic review. Int J Behav Nutr Phys Act.

[150] Vries LP, Baselmans BML, Bartels M (2020). Smartphone-based ecological momentary assessment of well-being: a systematic review and recommendations for future studies. J Happiness Stud.

[151] Guthold R, Stevens GA, Riley LM, Bull FC (2018). Worldwide trends in insufficient physical activity from 2001 to 2016: a pooled analysis of 358 population-based surveys with 1·9 million participants. Lancet Glob Health.

[152] Matthews CE, Carlson SA, Saint-Maurice PF, Patel S, Salerno EA, Loftfield E (2021). Sedentary behavior in US adults: fall 2019. Med Sci Sports Exerc.

[153] Giurgiu M, Niermann C, Ebner-Priemer U, Kanning M (2020). Accuracy of sedentary behavior-triggered ecological momentary assessment for collecting contextual information: development and feasibility study. JMIR mHealth uHealth.

[154] Kanning M, Ebner-Priemer U, Schlicht W (2015). Using activity triggered e-diaries to reveal the associations between physical activity and affective states in older adult’s daily living. Int J Behav Nutr Phys Act.

[155] Bossmann T, Kanning M, Koudela-Hamila S, Hey S, Ebner-Priemer UW (2013). The association between short periods of everyday life activities and affective states: a replication study using ambulatory assessment. Front Psychol.

[156] Haaren-Mack B, Loeffler SN, Haertel S, Anastasopoulou P, Stumpp J, Hey S (2013). Characteristics of the activity-affect association in inactive people: an ambulatory assessment study in daily life. Front Psychol.

[157] Stavrakakis N, Booij SH, Roest AM, De Jonge P, Oldehinkel AJ, Bos EH (2015). Temporal dynamics of physical activity and affect in depressed and nondepressed individuals. Health Psychol.

[158] Arend MG, Schäfer T (2019). Statistical power in two-level models: a tutorial based on Monte Carlo simulation. Psychol Methods.

[159] Santangelo P, Reinhard I, Mussgay L, Steil R, Sawitzki G, Klein C (2014). Specificity of affective instability in patients with borderline personality disorder compared to posttraumatic stress disorder, bulimia nervosa, and healthy controls. J Abnorm Psychol.

[160] Epstein DH, Marrone GF, Heishman SJ, Schmittner J, Preston KL (2010). Tobacco, cocaine, and heroin: craving and use during daily life. Addict Behav.

[161] Heinz A, Kiefer F, Smolka MN, Endrass T, Beste C, Beck A (2020). Addiction Research Consortium: losing and regaining control over drug intake (ReCoDe)-From trajectories to mechanisms and interventions. Addict Biol.

[162] Dunton GF, Liao Y, Intille S, Huh J, Leventhal A (2015). Momentary assessment of contextual influences on affective response during physical activity. Health Psychol.

[163] Bejarano CM, Cushing CC, Crick CJ (2019). Does context predict psychological states and activity? An ecological momentary assessment pilot study of adolescents. Psychol SPORT Exerc.

[164] Boyle HK, Dunsiger SI, Bohlen LC, Emerson JA, Lee HH, Stevens CJ (2020). Affective response as a mediator of the association between the physical and social environment and physical activity behavior. J Behav Med.

[165] Sallis JF, Prochaska JJ, Taylor WC (2000). A review of correlates of physical activity of children and adolescents. Med Sci Sports Exerc.

[166] Doherty ST, Lemieux CJ, Canally C (2014). Tracking human activity and well-being in natural environments using wearable sensors and experience sampling. Soc Sci Med.

[167] Dunton GF, Liao Y, Intille S, Wolch J, Pentz MA (2011). Physical and social contextual influences on children’s leisure-time physical activity: an ecological momentary assessment study. J Phys Act Health.

[168] Thompson Coon J, Boddy K, Stein K, Whear R, Barton J, Depledge MH (2011). Does participating in physical activity in outdoor natural environments have a greater effect on physical and mental wellbeing than physical activity indoors? A systematic review. Environ Sci Technol.

[169] Tost H, Reichert M, Braun U, Reinhard I, Peters R, Lautenbach S (2019). Neural correlates of individual differences in affective benefit of real-life urban green space exposure. Nat Neurosci.

[170] Yang C-H, Maher JP, Ponnada A, Dzubur E, Nordgren R, Intille S (2021). An empirical example of analysis using a two-stage modeling approach: within-subject association of outdoor context and physical activity predicts future daily physical activity levels. Transl Behav Med..

[171] Timm I, Reichert M, Ebner-Priemer UW, Giurgiu M (2023). Momentary within-subject associations of affective states and physical behavior are moderated by weather conditions in real life: an ambulatory assessment study. Int J Behav Nutr Phys Act.

[172] Sers S, Timm I, Vries EA, Wäsche H, Woll A, Bender O (2023). Insights on physical behavior while working from home: an ecological momentary assessment study. Scand J Med Sci Sports.

[173] Kanning M, Do B, Mason TB, Belcher BR, Yang C-H, Dunton GF (2020). Doing exercise or sport together with one’s child is positively associated with mothers’ momentary affect in daily life, but not with higher levels of overall physical activity. BMC Public Health.

[174] Dunton GF, Liao Y, Dzubur E, Leventhal AM, Huh J, Gruenewald T (2015). Investigating within-day and longitudinal effects of maternal stress on children’s physical activity, dietary intake, and body composition: protocol for the MATCH study. Contemp Clin Trials.

[175] Kim J, Conroy DE, Smyth JM (2020). Bidirectional associations of momentary affect with physical activity and sedentary behaviors in working adults. Ann Behav Med.

[176] Dunton GF, Wang W-L, Intille SS, Dzubur E, Ponnada A, Hedeker D (2022). How acute affect dynamics impact longitudinal changes in physical activity among children. J Behav Med.

[177] Smith KE, Mason TB, Wang W-L, Schumacher LM, Pellegrini CA, Goldschmidt AB (2022). Dynamic associations between anxiety, stress, physical activity, and eating regulation over the course of a behavioral weight loss intervention. Appetite.

[178] Brand R, Ekkekakis P (2018). Affective-reflective theory of physical inactivity and exercise: foundations and preliminary evidence. Ger J Exerc Sport Res..

[179] Granger CWJ (1969). Investigating causal relations by econometric models and cross-spectral methods. Econometrica.

[180] Molenaar PCM (2019). Granger causality testing with intensive longitudinal data. Prev Sci.

[181] Schuurman NK, Ferrer E, De Boer-Sonnenschein M, Hamaker EL (2016). How to compare cross-lagged associations in a multilevel autoregressive model. Psychol Methods.

[182] Schmiedek F, Neubauer AB (2020). Experiments in the wild: introducing the within-person encouragement design. Multivar Behav Res.

[183] D’Alfonso S (2020). AI in mental health. Curr Opin Psychol.

[184] Reeves B, Robinson T, Ram N (2020). Time for the human screenome project. Nature.

[185] Sano A, Taylor S, McHill AW, Phillips AJK, Barger LK, Klerman E (2018). Identifying objective physiological markers and modifiable behaviors for self-reported stress and mental health status using wearable sensors and mobile phones: observational study. J Med Internet Res.

[186] Mehl MR, Gosling SD, Pennebaker JW (2006). Personality in its natural habitat: manifestations and implicit folk theories of personality in daily life. J Pers Soc Psychol.

[187] Haaren-Mack B, Bussmann JBJ, Ebner-Priemer UW. Physical activity monitoring. In: Paul RH, Salminen LE, Heaps J, Cohen LM, editors. The Wiley encyclopedia of health psychology. Hoboken: Wiley; 2020. pp. 447–457. 10.1002/9781119057840.ch95.

[188] Mehl MR, Eid M, Wrzus C, Harari GM, Ebner-Priemer UW (2024). Mobile sensing in psychology: methods and applications.

[189] Bai Y, Copeland WE, Burns R, Nardone H, Devadanam V, Rettew J (2022). Ecological momentary assessment of physical activity and wellness behaviors in college students throughout a school year: longitudinal naturalistic study. JMIR Public Health Surveill.

[190] Bourke M, Hilland TA, Craike M (2021). Contextual influences on the within-person association between physical activity and affect in adolescents: an ecological momentary assessment study. J Behav Med.

[191] Bourke M, Hilland TA, Craike M (2023). Domain specific association between physical activity and affect in adolescents’ daily lives: an ecological momentary assessment study. Psychol Health.

[192] Curtiss JE, Pinaire M, Fulford D, McNally RJ, Hofmann SG (2022). Temporal and contemporaneous network structures of affect and physical activity in emotional disorders. J Affect Disord.

[193] DeMasi O, Feygin S, Dembo A, Aguilera A, Recht B (2017). Well-being tracking via smartphone-measured activity and sleep: cohort study. JMIR Mhealth Uhealth.

[194] Difrancesco S, Penninx BWJH, Merikangas KR, Van Hemert AM, Riese H, Lamers F (2022). Within-day bidirectional associations between physical activity and affect: a real-time ambulatory study in persons with and without depressive and anxiety disorders. Depress Anxiety.

[195] Elavsky S, Kishida M, Mogle JA (2016). Concurrent and lagged relations between momentary affect and sedentary behavior in middle-aged women. Menopause.

[196] Hevel DJ, Dunton GF, Maher JP (2020). Acute bidirectional relations between affect, physical feeling states, and activity-related behaviors among older adults: an ecological momentary assessment study. Ann Behav Med Publ Soc Behav Med.

[197] Jeckel S, Sudeck G (2018). Sport activities in daily routine: situational associations between individual goals, activity characteristics, and affective well-being. Ger J Exerc Sport Res.

[198] Kanning M, Ebner-Priemer U, Brand R (2012). Autonomous regulation mode moderates the effect of actual physical activity on affective states: an ambulant assessment approach to the role of self-determination. J Sport Exerc Psychol.

[199] Kanning M (2013). Using objective, real-time measures to investigate the effect of actual physical activity on affective states in everyday life differentiating the contexts of working and leisure time in a sample with students. Front Psychol.

[200] Kanning M, Niermann C, Ebner-Primer U, Giurgiu M (2021). The context matters - not all prolonged sitting bouts are equally related to momentary affective states: an ambulatory assessment with sedentary-triggered E-diaries. Int J Behav Nutr Phys Act.

[201] Kracht CL, Beyl RA, Maher JP, Katzmarzyk PT, Staiano AE (2021). Adolescents’ sedentary time, affect, and contextual factors: an ecological momentary assessment study. Int J Behav Nutr Phys Act.

[202] Kuehnhausen J, Leonhardt A, Dirk J, Schmiedek F (2013). Physical activity and affect in elementary school children’s daily lives. Front Psychol.

[203] Le F, Yap Y, Tung NYC, Bei B, Wiley JF (2022). The associations between daily activities and affect: a compositional isotemporal substitution analysis. Int J Behav Med.

[204] Li Y-M, Konstabel K, Mõttus R, Lemola S (2022). Temporal associations between objectively measured physical activity and depressive symptoms: an experience sampling study. Front Psychiatry.

[205] Liao Y, Chou C-P, Huh J, Leventhal A, Dunton G (2017). Examining acute bi-directional relationships between affect, physical feeling states, and physical activity in free-living situations using electronic ecological momentary assessment. J Behav Med.

[206] Liao Y, Chou C-P, Huh J, Leventhal A, Dunton G (2017). Associations of affective responses during free-living physical activity and future physical activity levels: an ecological momentary assessment study. Int J Behav Med.

[207] McLean DC, Nakamura J, Csikszentmihalyi M (2018). Reconsidering the experience machine: self-reported versus objective measures of physical activity to increase positive affect. J Health Psychol.

[208] Michalak J, Niemeyer H, Tschacher W, Baumann N, Chi Zhang X, Adolph D (2022). Subjective and objective measures of activity in depressed and non-depressed individuals in everyday life. J Exp Psychopathol.

[209] Pannicke B, Reichenberger J, Schultchen D, Pollatos O, Blechert J (2020). Affect improvements and measurement concordance between a subjective and an accelerometric estimate of physical activity. Eur J Health Psychol.

[210] Pinto BM, Kindred MD, Dunsiger SI, Williams DM (2021). Sedentary behavior among breast cancer survivors: a longitudinal study using ecological momentary assessments. J Cancer Surviv Res Pract.

[211] Poppe L, Paepe AL, van Ryckeghem DML, van Dyck D, Maes I, Crombez G (2021). The impact of mental and somatic stressors on physical activity and sedentary behaviour in adults with type 2 diabetes mellitus: a diary study. PeerJ.

[212] Powell R, Allan JL, Johnston DW, Gao C, Johnston M, Kenardy J (2009). Activity and affect: repeated within-participant assessment in people after joint replacement surgery. Rehabil Psychol.

[213] Shin GD (2020). Investigating the impact of daily life context on physical activity in terms of steps information generated by wearable activity tracker. Int J Med Inf.

[214] Smith KE, Mason TB, O’Connor SM, Wang S, Dzubur E, Crosby RD (2021). Bi-directional associations between real-time affect and physical activity in weight-discordant siblings. J Pediatr Psychol.

[215] Sudeck G, Jeckel S, Schubert T (2018). Individual differences in the competence for physical-activity-related affect regulation moderate the activity-affect association in real-life situations. J Sport Exerc Psychol.

[216] Takano K, Sakamoto S, Tanno Y (2013). Ruminative self-focus in daily life: associations with daily activities and depressive symptoms. Emotion.

[217] Vetrovsky T, Omcirk D, Malecek J, Stastny P, Steffl M, Tufano JJ (2021). Morning fatigue and structured exercise interact to affect non-exercise physical activity of fit and healthy older adults. BMC Geriatr.

[218] Walsh RFL, Smith LT, Titone MK, Ng TH, Goel N, Alloy LB (2022). The relationship between physical activity states and depressive symptoms: using ambulatory assessment to characterize day-to-day associations among individuals with and without bipolar spectrum disorder. Depress Anxiety.

[219] Williams DR, Martin SR, Liu S, Rast P (2020). Bayesian multivariate mixed-effects location scale modeling of longitudinal relations among affective traits, states, and physical activity. Eur J Psychol Assess.

