# Supplementary material for: The Within-Subject Association of Physical Behavior and Affective Well-Being in Everyday Life: A Systematic Literature Review
Source: Sports Med. 2024 May 6;54(6):1667–705. doi: 10.1007/s40279-024-02016-1 (PMC11239742; doi:10.1007/s40279-024-02016-1)
Supplement: Supplementary file 1 — Distribution of reviewed studies (DOCX 29 KB) [file 40279_2024_2016_MOESM1_ESM.docx]

**ESM Fig 1** Accumulated number of studies included in this review by year of publication.
