# Supplementary material for: The Within-Subject Association of Physical Behavior and Affective Well-Being in Everyday Life: A Systematic Literature Review
Source: Sports Med. 2024 May 6;54(6):1667–705. doi: 10.1007/s40279-024-02016-1 (PMC11239742; doi:10.1007/s40279-024-02016-1)
Supplement: Supplementary file 3 — Comprehensive search term (DOCX 13 KB) [file 40279_2024_2016_MOESM3_ESM.docx]

**ESM 3.** Detailed search terms in three databases.

| Database | Complete search term |
| --- | --- |
| Scopus: | TITLE-ABS-KEY ( "physical activit*" OR exercis* OR "sedentary behav*" OR sedentar* OR "physical inactivit*" ) AND TITLE-ABS-KEY ( mood* OR emotion* OR affect OR "affec* stat*" OR valence OR calmness OR "energetic arousal" ) AND TITLE-ABS-KEY ( "ambulatory assessment" OR "ecological momentary assessment" OR "experience sampling method*" OR "electronic sampling method" OR "ambulatory monitoring" OR acceler* OR "physical activity monitoring" OR "interactive assessment" OR "e*diar*" OR "electronic diar*" ) AND ( LIMIT-TO ( LANGUAGE , "English" ) ) |
| Web of Science: | ((TS=("physical activit*" OR exercis* OR "sedentary behav*" OR sedentar* OR "physical inactivit*") AND TS=(mood* OR emotion* OR affect OR "affec* stat*" OR valence OR calmness OR "energetic arousal") AND TS=("ambulatory assessment" OR "ecological momentary assessment" OR "experience sampling method*" OR "electronic sampling method" OR "ambulatory monitoring" OR acceler* OR "physical activity monitoring" OR "interactive assessment" OR "e*diar*" OR "electronic diar*"))) AND LANGUAGE: (English) |
| PubMed: | ((physical activity[Title/Abstract] OR exercise[Title/Abstract] OR exercises[Title/Abstract] OR sedentary behavior[Title/Abstract] OR sedentary behaviour[Title/Abstract] OR sedentariness[Title/Abstract] OR physical inactivity[Title/Abstract]) AND (mood[Title/Abstract] OR moods[Title/Abstract] OR emotion[Title/Abstract] OR emotions[Title/Abstract] OR affect[Title/Abstract] OR affects[Title/Abstract] OR affective state[Title/Abstract] OR affective states[Title/Abstract] OR affective-states[Title/Abstract] OR valence[Title/Abstract] OR calmness[Title/Abstract] OR energetic arousal[Title/Abstract])) AND (ambulatory assessment[Title/Abstract] OR ecological momentary assessment[Title/Abstract] OR experience sampling method[Title/Abstract] OR (("electronics"[MeSH Terms] OR "electronics"[All Fields] OR "electronic"[All Fields]) AND sampling method[Title/Abstract]) OR ambulatory monitoring[Title/Abstract] OR accelerometry[Title/Abstract] OR accelerometer[Title/Abstract] OR physical activity monitoring[Title/Abstract] OR interactive assessment[Title/Abstract] OR e-diary[Title/Abstract] OR ediary[Title/Abstract] OR ediaries[Title/Abstract] OR electronic diary[Title/Abstract] OR electronic diaries[Title/Abstract]) AND English[lang] |
