# Supplementary material for: The Within-Subject Association of Physical Behavior and Affective Well-Being in Everyday Life: A Systematic Literature Review
Source: Sports Med. 2024 May 6;54(6):1667–705. doi: 10.1007/s40279-024-02016-1 (PMC11239742; doi:10.1007/s40279-024-02016-1)
Supplement: Supplementary file 4 — Adapted Quality Assessment Tool (DOCX 21 KB) [file 40279_2024_2016_MOESM4_ESM.docx]

**ESM 4.** **Adapted Quality Assessment Tool**

| **Topic** |  | **Item** | **Description** |
| --- | --- | --- | --- |
| Title | | | |
|  | Title |  |  |
|  |  | 1 | 1. Include ambulatory assessment or ecological momentary assessment in title |
| Introduction | | | |
|  | Rationale | 2 | 1. Briefly introduce the concept of AA and provide reasons for utilizing AA for this study or topic of interests (e.g., to examine time-varying predictors of unhealthy eating occasions in children’s daily lives) |
| Methods | | | |
|  | Participant Training | 3 | 1. Indicate if, and by what methods, training of participants for AA protocol was used 2. Indicate if, and by what methods, training of participants for accelerometer was used (how to wear device correctly; placement, wearing times, data protection and return of the device) |
|  | AA Technology | 4 | Describe what technology, if any, was used. Include the following information:   1. Device (e.g., mobile phone, portable computer), 2. Model (e.g., Nexus 4), 3. Operating system (e.g., android, windows), 4. EMA program name |
|  | ACC Technology | 5 | 1. Sensor brand and model of ACC (e.g., Actigraph accelerometer GT1 M) 2. Operating system (e.g., data merger movisens) 3. Sensor placement (must indicate location and side of the body) 4. Sampling frequency (Hz), 5. Signal axes used (x, y, z) 6. Filter (low, high, band, e.g., low-frequency extension filter) |
|  | Assessment duration | 6 | 1. AA: state the number of days each wave of the study lasted, 2. AA: and how many weekdays versus weekend days 3. ACC: State the number of days each wave of the study lasted 4. ACC: and how many weekdays versus weekend days |
|  | AA prompting design | 7 | 1. Indicate the prompting strategy used for the study (e.g., event-based, interval-based, or a combination of the two). If using interval-based strategy, indicate what type of schedule is used (e.g., fixed, random, or hybrid interval) |
|  | AA prompt frequency | 8 | 1. Intended frequency of prompts per day. Break down by weekdays and weekend days if applicable |
|  | Parameterization | 9 | 1. AA: the origin of items: the source of items used in the AA (e.g., existing AA questionnaire, self-made) 2. ACC: accelerometer signals “outcome metric” (counts, time, intensity, type of PB) 3. ACC: epoch length used (sec, min, h) |
|  | Design features | 10 | 1. Describe any design feature to address potential sources of bias (e.g., reactivity; devices that display the archived activity) or participant burden (e.g., AA questions appearing in different orders) |
|  | Statistical methods | 11 | 1. Psychometric properties of items (Cronbach’s alpha, omega) 2. Model description (formula and / or text) 3. Centering (person, group, grand mean) 4. Random versus fixed effects 5. Measurements (p, Beta, effect size) |
|  | Criteria for defining non-wear / non-wear-time definition (exclusion criteria) | 12 | 1. Of AA (e.g., <30% responses to e‐diary prompts) 2. Of ACC (e.g., ≥60 min of continuous 0s) |
| Results | | | |
|  | Latency | 13 | 1. Report the amount of time from prompt signal to answering of prompt |
|  | Delay possibility | 14 | 1. Ability to suspend/delay responses |
|  | Compliance rate and missing data | 15 | Describe final data set:   1. Report total answered AA prompts across all subjects 2. The average number of AA prompts answered per person/group (report compliance rate both by monitoring days and waves, if applicable) 3. Indicate reasons for noncompliance, if known 4. ACC: report total wear time across all subjects 5. ACC: report total wear time per person 6. Indicate reasons for noncompliance, if known (number of participants non-compliant or who had accelerometer malfunction issues) |
| Discussion | | | |
|  | Limitations | 16 | 1. Discuss sources of potential bias when using AA methods / ACC (e.g., reactivity, use of technology, sampling, …) |

Abbreviations: AA = ambulatory assessment; ACC = accelerometry; PB = physical behavior; HZ = hertz; sec = seconds; min = minutes; h = hours

**Table S2a. Detailed valuation process:**

| 1 item | true = 1  false = 0 |
| --- | --- |
| 2 items | 2 true = 1  1 true / 1 false = 0,5  2 false = 0 |
| 3 items | 3 true = 1  2 true / 1 false = 0,5  1 true / 2 false = 0  0 true /3 false = 0 |
| 4 items | 4 true = 1  3 true = 1  2 true = 0,5  1 true = 0  0 true = 0 |
| 5 items | 5 true = 1  4 true = 1  3 true = 0,5  2 true = 0  1 true = 0  0 true = 0 |
| 6 items | 6 true = 1  5 true = 1  4 true = 1  3 true = 0,5  2 true = 0  1 true = 0  0 true = 0 |
