# Supplementary material for: The Within-Subject Association of Physical Behavior and Affective Well-Being in Everyday Life: A Systematic Literature Review
Source: Sports Med. 2024 May 6;54(6):1667–705. doi: 10.1007/s40279-024-02016-1 (PMC11239742; doi:10.1007/s40279-024-02016-1)
Supplement: Supplementary file 5 — Detailed Quality Assessment (DOCX 45 KB) [file 40279_2024_2016_MOESM5_ESM.docx]

|  | Bai et al. [189] | Bossmann et al. [155] | Bourke et al. [190] | Bourke et al. [191] | Cabrita et al. [135] | Curtiss et al. [192] | Cushing et al. [81] | Cushing et al. [129] | DeMasi et al. [193] | Difrancesco et al. [194] | Dunton et al. [162] | Elavsky et al. [195] | Giurgiu et al. [89] |
| --- | --- | --- | --- | --- | --- | --- | --- | --- | --- | --- | --- | --- | --- |
| **1. Title** | 1 | 1 | 1 | 1 | 0 | 0 | 0 | 1 | 0 | 1 | 1 | 0 | 0 |
| **2. Rationale** | 0 | 1 | 0 | 0 | 1 | 0 | 1 | 0 | 1 | 1 | 0 | 1 | 1 |
| **3. Training** | 0 | 0.5 | 0.5 | 0.5 | 1 | 0.5 | 1 | 1 | 0 | 0 | 0 | 0.5 | 1 |
| **4. AA technology** | 1 | 1 | 0.5 | 0.5 | 0.5 | 1 | 1 | 0.5 | 1 | 0 | 1 | 1 | 1 |
| **5. ACC technology** | 0.5 | 1 | 1 | 1 | 0.5 | 0 | 1 | 1 | 0.5 | 0 | 0 | 0.5 | 1 |
| **6. Assessment duration** | 0.5 | 0.5 | 1 | 1 | 0.5 | 0.5 | 0.5 | 0.5 | 0.5 | 0.5 | 1 | 0.5 | 1 |
| **7. AA prompting design** | 1 | 1 | 1 | 1 | 1 | 0 | 1 | 1 | 1 | 1 | 1 | 1 | 1 |
| **8. AA prompt frequency** | 1 | 0 | 1 | 1 | 1 | 1 | 1 | 1 | 1 | 1 | 1 | 1 | 1 |
| **9. Parameterization** | 0.5 | 1 | 1 | 1 | 0.5 | 0 | 1 | 1 | 0.5 | 1 | 1 | 1 | 1 |
| **10. Design features** | 0 | 0 | 0 | 1 | 1 | 0 | 1 | 0 | 1 | 1 | 1 | 0 | 1 |
| **11. Statistical methods** | 0 | 1 | 1 | 1 | 0 | 0 | 1 | 1 | 0.5 | 0 | 1 | 1 | 1 |
| **12. Defining non-wear** | 1 | 0 | 0.5 | 1 | 0 | 0 | 0.5 | 0.5 | 1 | 0 | 0.5 | 1 | 1 |
| **13. Latency** | 1 | 0 | 0 | 1 | 0 | 0 | 0 | 0 | 0 | 0 | 0 | 0 | 0 |
| **14. Delay possibility** | 0 | 1 | 1 | 1 | 0 | 0 | 0 | 0 | 0 | 0 | 0 | 0 | 1 |
| **15. Compliance rate/missing data** | 0 | 0 | 0.5 | 1 | 0.5 | 0.5 | 0 | 0 | 0 | 0.5 | 0.5 | 0.5 | 0.5 |
| **16. Limitations** | 1 | 1 | 1 | 1 | 1 | 1 | 1 | 1 | 1 | 0 | 1 | 1 | 0 |
| **Total score** | **8.5** | **10** | **11** | **14** | **8.5** | **4.5** | **11** | **9.5** | **9** | **7** | **10** | **10** | **12.5** |

|  | Giurgiu et al. [90] | Giurgiuet al. [86] | Giurgiu et al. [94] | Haaren-Mack et al. [156] | Hevel et al. [196] | Jeckel & Sudeck [197] | Jeckel & Sudeck [112] | Kanning [199] | Kanning & Schoebi [87] | Kanning et al. [173] | Kanning et al. [198] | Kanning et al. [154] | Kanning et al. [200] |
| --- | --- | --- | --- | --- | --- | --- | --- | --- | --- | --- | --- | --- | --- |
| **1. Title** | 1 | 0 | 0 | 1 | 1 | 0 | 0 | 0 | 0 | 0 | 1 | 0 | 1 |
| **2. Rationale** | 1 | 1 | 1 | 1 | 1 | 1 | 0 | 1 | 1 | 1 | 1 | 1 | 1 |
| **3. Training** | 1 | 1 | 1 | 1 | 1 | 1 | 1 | 0 | 0 | 0.5 | 0 | 1 | 0.5 |
| **4. AA technology** | 1 | 1 | 1 | 1 | 1 | 1 | 1 | 0.5 | 0.5 | 1 | 1 | 1 | 1 |
| **5. ACC technology** | 1 | 1 | 1 | 1 | 0 | 0.5 | 0.5 | 1 | 1 | 0 | 1 | 1 | 1 |
| **6. Monitoring period** | 1 | 1 | 1 | 0.5 | 0.5 | 1 | 1 | 1 | 1 | 0.5 | 0.5 | 1 | 1 |
| **7. AA prompting design** | 1 | 1 | 1 | 1 | 1 | 1 | 1 | 1 | 1 | 1 | 1 | 1 | 1 |
| **8. AA prompt frequency** | 1 | 1 | 1 | 1 | 1 | 1 | 1 | 1 | 1 | 1 | 1 | 1 | 1 |
| **9. Parameterization** | 1 | 1 | 1 | 1 | 1 | 1 | 1 | 1 | 1 | 1 | 1 | 1 | 1 |
| **10. Design features** | 0 | 1 | 1 | 0 | 0 | 0 | 1 | 1 | 0 | 0 | 0 | 1 | 1 |
| **11. Statistical methods** | 1 | 1 | 1 | 0 | 0.5 | 1 | 1 | 1 | 0.5 | 1 | 1 | 1 | 1 |
| **12. Defining non-wear** | 0.5 | 0 | 1 | 0 | 0.5 | 0 | 1 | 0 | 0.5 | 0.5 | 0 | 0 | 0 |
| **13. Latency** | 0 | 0 | 0 | 1 | 0 | 0 | 0 | 0 | 1 | 0 | 0 | 0 | 0 |
| **14. Delay possibility** | 1 | 1 | 1 | 1 | 1 | 1 | 1 | 1 | 1 | 1 | 0 | 0 | 0 |
| **15. Compliance rate/missing data** | 0.5 | 0.5 | 1 | 0 | 0 | 0.5 | 0.5 | 0 | 0 | 0 | 0 | 1 | 0 |
| **16. Limitations** | 1 | 1 | 1 | 1 | 1 | 1 | 1 | 1 | 1 | 1 | 1 | 0 | 1 |
| **Total score** | **13** | **12.5** | **14** | **11.5** | **10.5** | **11** | **12** | **10.5** | **10.5** | **9.5** | **9.5** | **11** | **11.5** |

|  | Kim et al. [175] | Kim et al. [125] | Kim et al. [126] | Koch et al. [80] | Koch et al. [93] | Koch et al. [91] | Kracht et al. [201] | Kuehnhausen et al. [202] | Langguth et al. [127] | Le at al. [203] | Li et al. [204] | Liao et al. [206] | Liao et al. [205] |
| --- | --- | --- | --- | --- | --- | --- | --- | --- | --- | --- | --- | --- | --- |
| **1. Title** | 0 | 1 | 0 | 0 | 1 | 0 | 1 | 0 | 0 | 0 | 1 | 1 | 1 |
| **2. Rationale** | 1 | 1 | 1 | 1 | 1 | 1 | 1 | 1 | 0 | 0 | 0 | 1 | 1 |
| **3. Training** | 1 | 1 | 1 | 0 | 1 | 1 | 1 | 0 | 1 | 0 | 0.5 | 0 | 1 |
| **4. AA technology** | 0 | 0.5 | 0.5 | 0.5 | 1 | 1 | 1 | 0 | 0.5 | 0.5 | 1 | 0 | 1 |
| **5. ACC technology** | 0 | 1 | 1 | 1 | 1 | 1 | 0 | 1 | 1 | 0.5 | 1 | 0 | 0 |
| **6. Assessment duration** | 1 | 1 | 0.5 | 0.5 | 1 | 0.5 | 1 | 0.5 | 1 | 0.5 | 0.5 | 1 | 1 |
| **7. AA prompting design** | 1 | 1 | 1 | 1 | 1 | 1 | 1 | 0 | 1 | 1 | 1 | 1 | 1 |
| **8. AA prompt frequency** | 1 | 1 | 1 | 1 | 1 | 1 | 1 | 1 | 1 | 1 | 1 | 1 | 1 |
| **9. Parameterization** | 0.5 | 1 | 1 | 1 | 1 | 1 | 1 | 0.5 | 1 | 1 | 1 | 0.5 | 1 |
| **10. Design features** | 1 | 0 | 1 | 0 | 0 | 1 | 1 | 1 | 0 | 0 | 1 | 0 | 1 |
| **11. Statistical methods** | 1 | 0.5 | 1 | 1 | 1 | 1 | 1 | 0.5 | 1 | 0.5 | 1 | 0 | 1 |
| **12. Defining non-wear** | 0.5 | 0 | 0.5 | 0.5 | 0.5 | 1 | 1 | 0.5 | 1 | 0.5 | 0 | 0.5 | 1 |
| **13. Latency** | 0 | 0 | 0 | 0 | 0 | 0 | 0 | 0 | 1 | 1 | 0 | 0 | 0 |
| **14. Delay possibility** | 0 | 1 | 0 | 0 | 1 | 1 | 1 | 0 | 0 | 0 | 0 | 0 | 1 |
| **15. Compliance rate/missing data** | 0.5 | 0.5 | 0 | 0 | 1 | 0.5 | 0.5 | 0.5 | 1 | 0 | 0 | 0 | 0.5 |
| **16. Limitations** | 1 | 1 | 1 | 0 | 1 | 0 | 1 | 0 | 1 | 1 | 1 | 0 | 1 |
| **Total score** | **9.5** | **11.5** | **10.5** | **7.5** | **13.5** | **12** | **13.5** | **6.5** | **11.5** | **7.5** | **10** | **6** | **13.5** |

|  | Madden et al. [95] | McLean et al. [207] | Merikangas et al. [18] | Michalak et al. [208] | Pannicke et al. [209] | Pinto et al. [210] | Poppe et al. [211] | Powell et al. [212] | Reichert et al. [88] | Reichert et al. [92] | Ruissen et al. [85] | Schwerdtfeger et al. [83] | Shin et al. [213] |
| --- | --- | --- | --- | --- | --- | --- | --- | --- | --- | --- | --- | --- | --- |
| **1. Title** | 0 | 0 | 0 | 0 | 0 | 1 | 0 | 0 | 0 | 1 | 0 | 1 | 0 |
| **2. Rationale** | 1 | 1 | 1 | 0 | 1 | 1 | 1 | 1 | 1 | 1 | 0 | 1 | 1 |
| **3. Training** | 1 | 1 | 0 | 0 | 1 | 1 | 0.5 | 1 | 1 | 1 | 1 | 1 | 0 |
| **4. AA technology** | 1 | 1 | 1 | 1 | 0 | 1 | 1 | 0.5 | 1 | 1 | 1 | 1 | 0.5 |
| **5. ACC technology** | 0 | 0.5 | 0 | 1 | 0 | 0 | 0.5 | 1 | 1 | 1 | 0.5 | 1 | 0 |
| **6. Monitoring period** | 0.5 | 0.5 | 0.5 | 0.5 | 1 | 1 | 0.5 | 0.5 | 0.5 | 0.5 | 0.5 | 1 | 1 |
| **7. AA prompting design** | 1 | 1 | 1 | 1 | 1 | 1 | 1 | 1 | 1 | 1 | 1 | 1 | 1 |
| **8. AA prompt frequency** | 1 | 1 | 1 | 1 | 1 | 1 | 1 | 1 | 1 | 1 | 1 | 1 | 1 |
| **9. Parameterization** | 1 | 0 | 0.5 | 1 | 1 | 1 | 1 | 0.5 | 1 | 1 | 1 | 1 | 0 |
| **10. Design features** | 1 | 1 | 1 | 1 | 1 | 0 | 1 | 0 | 0 | 0 | 1 | 1 | 0 |
| **11. Statistical methods** | 1 | 0 | 0 | 1 | 1 | 0 | 1 | 1 | 1 | 1 | 1 | 1 | 0 |
| **12. Defining non-wear** | 0.5 | 0.5 | 0 | 0 | 1 | 0 | 1 | 1 | 0.5 | 0.5 | 0.5 | 0.5 | 0 |
| **13. Latency** | 0 | 0 | 0 | 0 | 0 | 0 | 0 | 0 | 0 | 0 | 1 | 0 | 0 |
| **14. Delay possibility** | 1 | 0 | 0 | 0 | 1 | 0 | 1 | 1 | 1 | 1 | 0 | 1 | 1 |
| **15. Compliance rate/missing data** | 1 | 1 | 0 | 0 | 0.5 | 0.5 | 0 | 1 | 0.5 | 1 | 0 | 0 | 0 |
| **16. Limitations** | 1 | 1 | 0 | 1 | 1 | 1 | 1 | 1 | 1 | 1 | 1 | 1 | 1 |
| **Total score** | **12** | **9.5** | **6** | **8.5** | **11.5** | **9.5** | **11.5** | **11.5** | **11.5** | **13** | **10.5** | **13.5** | **6.5** |

|  | Smith et al. [131] | Smith et al. [214] | Stavrakakis et al. [157] | Stevenson et al. [82] | Sudeck et al. [215] | Takano et al. [216] | Vetrovsky et al. [217] | Walsh et al. [218] | Wen et al. [132] | Wilhelm et al. [136] | Williams et al. [219] | Yang et al. [79] | Zenk et al. [78] | Zhaoyang & Martire [77] |
| --- | --- | --- | --- | --- | --- | --- | --- | --- | --- | --- | --- | --- | --- | --- |
| **1. Title** | 0 | 0 | 0 | 1 | 0 | 0 | 0 | 1 | 0 | 0 | 0 | 1 | 1 | 0 |
| **2. Rationale** | 1 | 1 | 1 | 0 | 1 | 0 | 1 | 0 | 1 | 1 | 1 | 1 | 1 | 1 |
| **3. Training** | 1 | 1 | 1 | 1 | 1 | 1 | 1 | 0 | 1 | 1 | 0.5 | 0 | 1 | 0.5 |
| **4. AA technology** | 0.5 | 0 | 0.5 | 0.5 | 1 | 0 | 0.5 | 0 | 1 | 0 | 0 | 1 | 0 | 0 |
| **5. ACC technology** | 0.5 | 0.5 | 0.5 | 0.5 | 1 | 1 | 1 | 0.5 | 0.5 | 0.5 | 0 | 0 | 0.5 | 0.5 |
| **6. Assessment duration** | 1 | 1 | 0.5 | 0.5 | 0.5 | 1 | 1 | 0.5 | 0.5 | 0.5 | 0.5 | 1 | 0.5 | 0.5 |
| **7. AA prompting design** | 1 | 1 | 1 | 1 | 1 | 1 | 1 | 1 | 1 | 1 | 1 | 1 | 1 | 0 |
| **8. AA prompt frequency** | 1 | 1 | 1 | 1 | 1 | 1 | 1 | 1 | 1 | 1 | 1 | 1 | 1 | 1 |
| **9. Parameterization** | 1 | 1 | 1 | 1 | 1 | 1 | 1 | 1 | 1 | 1 | 1 | 1 | 1 | 0.5 |
| **10. Design features** | 0 | 0 | 1 | 1 | 0 | 1 | 0 | 0 | 1 | 0 | 1 | 1 | 0 | 0 |
| **11. Statistical methods** | 0,5 | 1 | 0 | 1 | 1 | 1 | 0.5 | 1 | 1 | 1 | 1 | 1 | 1 | 1 |
| **12. Defining non-wear** | 0.5 | 0.5 | 0.5 | 0 | 0 | 0.5 | 1 | 0 | 0.5 | 1 | 0 | 0.5 | 1 | 1 |
| **13. Latency** | 0 | 0 | 1 | 0 | 0 | 1 | 0 | 0 | 0 | 1 | 0 | 0 | 0 | 0 |
| **14. Delay possibility** | 0 | 0 | 0 | 0 | 1 | 0 | 1 | 0 | 1 | 0 | 0 | 1 | 0 | 0 |
| **15. Compliance rate/missing data** | 0.5 | 1 | 0 | 1 | 1 | 0.5 | 0.5 | 0 | 0 | 0 | 0 | 0 | 0 | 0.5 |
| **16. Limitations** | 1 | 1 | 1 | 1 | 1 | 1 | 1 | 1 | 1 | 1 | 0 | 1 | 1 | 1 |
| **Total score** | **9.5** | **10** | **10** | **10.5** | **11.5** | **11** | **11.5** | **7** | **11.5** | **10** | **7** | **11.5** | **10** | **7.5** |
