# Supplementary figures and images for: The Within-Subject Association of Physical Behavior and Affective Well-Being in Everyday Life: A Systematic Literature Review
Source: Sports Med. 2024 May 6;54(6):1667–705. doi: 10.1007/s40279-024-02016-1 (PMC11239742; doi:10.1007/s40279-024-02016-1)

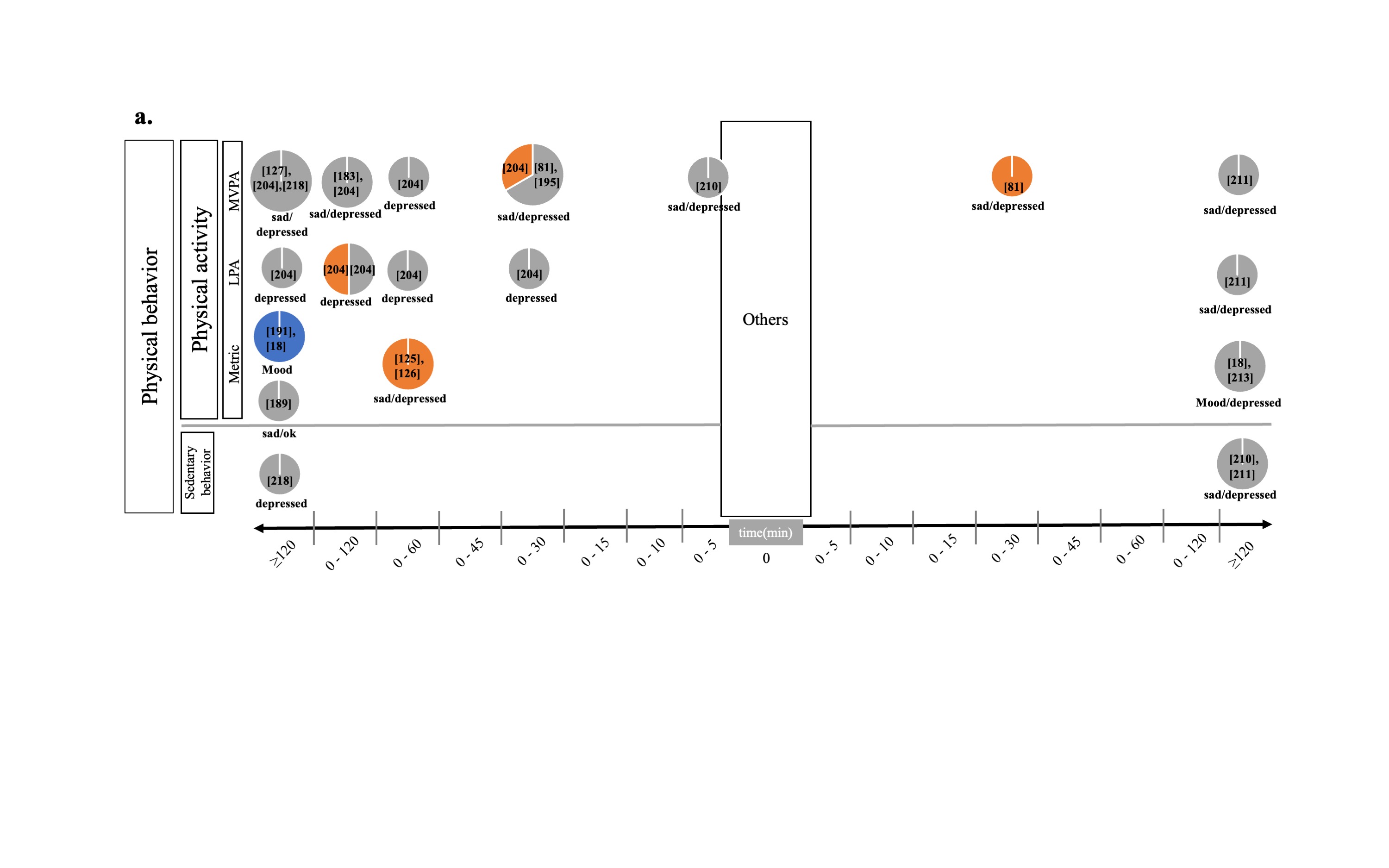

Supplement: Supplementary file 7 — Affective well-being and physical behavior (further items; a) (JPG 260 KB) [file 40279_2024_2016_MOESM7_ESM.jpg]

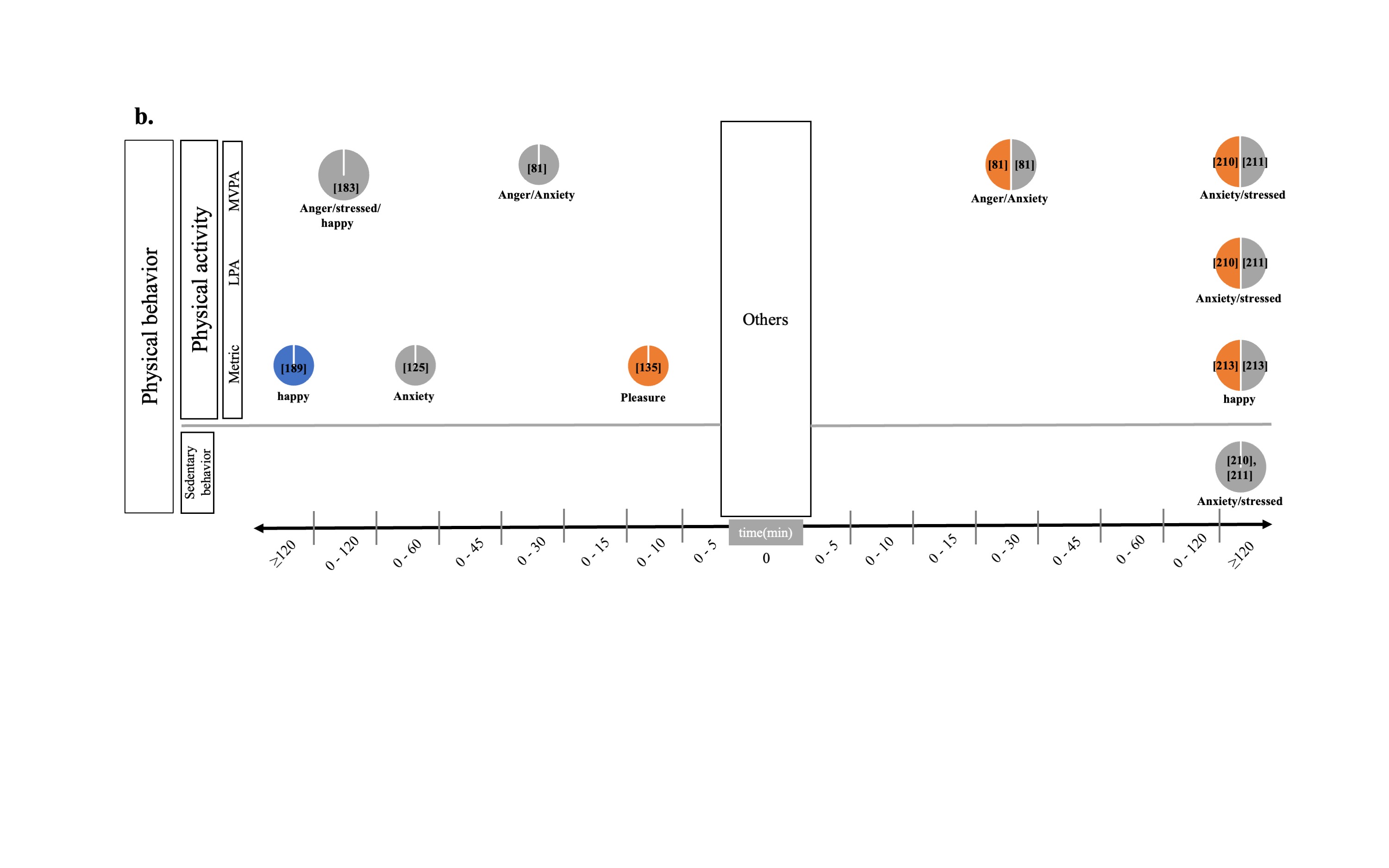

Supplement: Supplementary file 8 — Affective well-being and physical behavior (further items; b) (JPG 226 KB) [file 40279_2024_2016_MOESM8_ESM.jpg]
