# Supplementary material for: The Within-Subject Association of Physical Behavior and Affective Well-Being in Everyday Life: A Systematic Literature Review
Source: Sports Med. 2024 May 6;54(6):1667–705. doi: 10.1007/s40279-024-02016-1 (PMC11239742; doi:10.1007/s40279-024-02016-1)
Supplement: Supplementary file 9 — Modified quality assessment development (DOCX 14 KB) [file 40279_2024_2016_MOESM9_ESM.docx]

ESM 8. Overview of categories across QA tools and AA guidelines.

| **QA – Timm et al.** | **CREMAS – Liao et al.** | **Guidelines AA – Trull & Ebner-Priemer** |
| --- | --- | --- |
| Title | Title |  |
| Rationale | Rationale | Rationale |
| Training | Training | Training |
| AA Technology | AA Technology | Describe hardware and software |
| ACC Technology |  |  |
| Assessment duration | Assessment duration | Assessment duration |
| Prompt design | Prompt design | Prompt design |
| Parametrization |  | Parametrization |
| Design features | Design features | Design feature |
| Statistic |  | Statistic |
| Inclusion criteria |  |  |
| Latency | Latency |  |
| Compliance/Missing data | Compliance/Missing data | Compliance/Missing data |
| Limitation | Limitation |  |
|  | Wave duration | Justify sample size |
|  | Conclusion |  |
