# Supplementary material for: The Within-Subject Association of Physical Behavior and Affective Well-Being in Everyday Life: A Systematic Literature Review
Source: Sports Med. 2024 May 6;54(6):1667–705. doi: 10.1007/s40279-024-02016-1 (PMC11239742; doi:10.1007/s40279-024-02016-1)
Supplement: Supplementary file 10 — Average physical activity across the aggregated time frames (DOCX 35 KB) [file 40279_2024_2016_MOESM10_ESM.docx]

| **Study** | **n** | **Female %** | **Age**  mean (range) | **Participants characteristics** sample (specifics) | **Physical behavior assessment** unit; timeframe; direction; | **PB mean/day**  parameter in minutes/day or week: mean ± SD, range | **PB within the aggregated time frame**  parameter and unit/timeframe: mean ± SD, range |
| --- | --- | --- | --- | --- | --- | --- | --- |
| Bai et al. [189] | 805 | 71.3% | NR (18-25) | Adults (students) | Metric; 1440 min; before | Steps/d: NR ± NR, 8989-9566 (mo-fr); 8533 ± 286, NR (sa); 7327 ± 286, NR (su) | NR |
| Bossmann et al. [155] | 62 | 14.5% | 21.4 (19-30) | Adults (students) | Metric; 10 min; before; | NR | Milli-g/min: 62 ± 64.9,  12.8-765.2 |
| Bourke et al. [190] | 119 | 46.4% | 14.7 (NR) | Adolescents | MVPA; 15 min; before | NR | MET/15 min prior prompt: 2.59 ± NR, NR (recreational PA); 2.46 ± NR, NR (active travel); 2.34 ± NR, NR (household PA) |
| Bourke et al. [191] | 119 | 46.4% | 14.7 (13-17) | Adolescents | MVPA; 15 min; before; | NR | MVPA min/15 min prior prompt: 2.23 ± 2.43, NR |
| Cabrita et al. [135] | 10 | 60% | 68.7 (65-83) | Elders | Metric; 10 min; before | NR | NR |
| Curtiss et al. [192] | 34 | 73.53% | 28.97 (18-55) | Adults (MDD, anxiety) | NR; 0 min; NR | NR | NR |
| Cushing et al. [129] | 26 | 42.3% | 15.96 (13-18) | Adolescents | MVPA; 30 min; bidirectional | MVPA min/d: 30.63 ± 28.75, NR | MVPA min/30 min prior prompt: 1.14 ± 2.79, NR; MVPA min/30 min after prompt: 1,20 ± 2,94, NR |
| Cushing et al. [81] | 26 | 42.3% | 15.67 (13-18) | Adolescents | MVPA; 30 min; bidirectional | MVPA min/d: 30.63 ± 28.75, NR | MVPA min/30 min prior prompt: 1.14 ± 2.79, NR; MVPA min/30 min after prompt: 1.20 ± 2.94, NR |
| DeMasi et al. [193] | 53 | 49% | 19.83 (NR) | Adults (students) | Metric; 1440 min; before | Activity min/d: 118.78 ± 32.67, NR | NR |
| Difrancesco et al. [194] | 359 | 63.7 % | 49.5 (NR) | Adults (MDD, anxiety) | LPA, MVPA; 180 min; bidirectional | NR | NR |
| Dunton et al. [162] | 119 | 52% | NR (9-13) | Children  (Healthy PLACES) | MVPA; 30 min; bidirectional | MVPA min/d: 22.24 min ± 14.43, 4.39 –96.20 | MVPA min/15min window: 0.75 min ± 2.13, 0–30 |
| Elavsky et al. [195] | 121 | 100% | 51.5 (40-60) | Adults | SB; 180-360 min; bidirectional | SB h/d: 12.6 ± 1.7, NR | NR |
| Giurgiu et al. [90] | 92 | 63% | 33.7 (22-62) | Adults (university employee) | Metric, SB; 15-30 min; before | SB h/d: 7.2 ± 3.8, 5-22 | PA milli-g/min: 65.03 ± 15.3, 31.7-95.5 |
| Giurgiu et al. [86] | 92 | 65% | 33.73 (22-62) | Adults (university employee) | SB; 80 min; before | SB h/d: 7.6 ± 2.88, 0 – 16.09 | NR |
| Giurgiu et al. [94] | 92 | 65% | 33.88 (22-62) | Adults (university employee) | SB; 30 min; after | SB h/d: 8.03 ± 2.71, 1.55 – 16.09 | NR |
| Giurgiu et al. [89] | 103 | 55.1% | 22.1 (19.3-24.9) | Adults (students) | LPA, MVPA; 60 min; after | MVPA h/d: 1.15 ± 0.56, 0-3.53; LPA h/d: 5.02 ± 2.46, 0-13.91 | LPA min/60 min prior prompt: 21 ± NR, NR; MVPA min/60 min prior prompt: 1 ± NR, NR |
| Haaren et al. [156] | 29 | NR | 21.3 (NR) | Adults (students) | Metric, LPA; 15/-30 min; before | NR | MET across all 15 min episodes: 1.44 ± 0.42, NR. LPA min across all 15 min episodes: 1.62 ± 2.46, NR; MPA min across all 15 min episodes: 0.51 ± 1.38, NR; VPA min across all 15 min episodes: 0.04 ± 0.51, NR |
| Hevel et al. [196] | 103 | 62.5% | 72.4 (60-98) | Adults | Metric; 15/-30 min; bidirectional | Stepping min/d: 250.55 ± 119.36, NR; standing min/d: 90.72 ± 50.15, NR | Stepping min/15 min prior prompt: 1.49 ± 0.7, NR; Stepping min/15 min after prompt: 1.44 ± 0.65, NR; Stepping min/30 min prior prompt: 3.02 ± 1.36, NR; Stepping min/30 min after prompt: 2.94 ± 1.32, NR; Standing min/15 min prior prompt: 4.02 ± 1.58, NR; Standing min/15 min after prompt: 4.08 ± 1.63, NR; Standing min/30 min prior prompt: 8.02 ± 3.12, NR; Standing min/30 min after prompt: 8.06 ± 3.18, NR |
| Jeckel & Sudeck [112] | 46 | 54.4% | 32 (21-59) | Adults | Metric; 15/-720 min; before | MET/h: 5.41 ± 3.09, 0.64-13.96 | NR |
| Jeckel & Sudeck [197] | 46 | 54.4% | 32 (21-59) | Adults | Metric; 15 min; bidirectional; | MET/h: 5.41 ± 3.09, 0.64-13.96 | NR |
| Kanning et al. [198] | 44 | 47.7% | 26.2 (NR) | Adults (students) | Metric; 10 min; before | NR | Milli-g/min across all 10-min episodes: 77.3 ± 94.3, 0.8-994.4 |
| Kanning [199] | 87 | 54% | 24.6 (NR) | Adults (students) | Metric; 10 min; before | NR | Milli-g/min: 84.4 ± NR, 0.4 – 994.4 |
| Kanning et al. [154] | 74 | 49% | 60.1 (50-70) | Adults | Metric; 10 min; before | NR | Milli-g/10 min prior prompt: 105.5 ± 137.3, 0.01-1307.5 |
| Kanning & Schoebi [87] | 65 | 57% | 24.6 (NR) | Adults (students) | Metric; 5-/45 min; after | NR | Milli-g/min: 90.25 ± 27.31, 0 – 1330.71 (between subject); 104.94 ± 47.42, 0-1330.71 (within subject) |
| Kanning et al. [173] | 202 | 100% | 41 (24-57) | Adults (mothers of 8 to 12-year-old children) | MVPA; 120 min; before | MVPA min/d: 21.35 ± 15.18, 1.56-86.56; LPA min/d: 198.52 ± 65.95, 29.06-397.75 | NR |
| Kanning et al. [200] | 308 | 50.3% | 27.4 (17-66) | Adults (students and employees) | SB; 30 min; before | NR | Sedentary bouts of 30 min: 5.4 ± 2.7, 1.8-15.6 (study 1); 2 ± 0.5, 1-3.4 (study 2); Sedentary bouts of 20 min: 8.7 ± 4.6, 1.5-20.5 (study 3); 6.4 ± 3.78, 1-17 (study 4) |
| Kim et al. [125] | 113 | 28.3% | adolescents: 13.6 (NR) undergraduates: 21.6 (NR) office workers: 41.0 (NR) | Adolescents, adults (undergraduates and office workers) | Metric; 60 min; bidirectional | NR | Locomotor activity/60 min around EMA: 144.44 ± 22.31, NR |
| Kim et al. [126] | 57 | MDD: 14.3% HC: 0% | MDD: 34 (22–42); HC: 40.7 (23–58) | Adults (with and without MDD) | Metric; 60 min; before | NR | Locomotor activity/60 min around EMA: 111.59 ± 5.08, NR (MDD); 132.61 ± 3.08, NR (HC) |
| Kim et al. [175] | 122 | 76.4% | 41.3 (19-63) | Adults | Metric; 5-/60-/120 min; bidirectional | NR | Activity counts prior prompt: 28.1–30.7 ± 16.3–19.5, 5.8–93.0; Activity counts after prompt: 25.2–29.9 ± 16.8–20.4, 1.4–116.3 |
| Koch et al. [93] | 113 | 48% | 15.02 (12-17) | Adolescents  (URGENY) | Metric; 10 min; after | Milli-g/participant/week: 40.86 ± 11.87, 13.32-74.78 | NR |
| Koch et al. [91] | 113 | 48% | 15.02 (12-17) | Adolescents  (URGENY) | Metric; 15 min; before | Milli-g/participant/week: 40.86 ± 11.87, 13.32-74.78 | NR |
| Koch et al. [80] | 185 | 54.1% | 26.65 (14-45) | Adolescents, adults (with and without ADHD) | Metric; 10 min; before | NR | NR |
| Kracht et al. [201] | 284 | 54% | 12.6 (10-16) | Adolescents (TIGER Kids study) | LPA, MVPA; 30 min; before | NR | MVPA min/30 min prior prompt: 1.0 ± 2.3, NR; LPA min/30 min prior prompt: 7.6 ± 5.7, NR |
| Kuehnhausen et al. [202] | 82 | 45% | 117.2 (97-132) months | Children (FLUX) | MVPA; 1440 min; before | Activity min/d: 103 ± 81, NR; VPA min/day: 21 ± 24, NR | NR |
| Langguth et al. [127] | 72 | 37% | 17.36 (12-26) | Adolescents | MVPA; 1440 min; before | Activity hours: 1.28 ± 0.48, NR (weekday); 0.92 ± 0.75, NR (weekend) | NR |
| Le et al. [203] | 361 | 72.5% | 22.79 (NR) | Adults | LPA, MVPA; 1140 min; NR | MVPA h/d: 2.56 ± 1.01, NR; LPA h/d: 8.71 ± 1.37, NR; | NR |
| Li et al. [204] | 78 | 71.79% | 25.46 (NR) | Adults | LPA, MVPA; 0-180 min | NR | Milli-g/min/0-180 min prior prompt: 66.13–70 ± 22.09–27.09, NR; MET/min/0-180 min prior prompt: 1.59–1.63 ± 0.19–0.25, NR; Milli-g/min/0-180 min after prompt: 64.34–68.94 ± 22.75–29.42 22.77, NR; MET/min/0-180 min after prompt: 1.58–1.62 ± 0.21–0.26, NR; |
| Liao et al. [205] | 117 | 72.5% | 40.4 (NR) | Adults  (MOBILE) | MVPA, LPA; 15-/30 min; bidirectional | MVPA min/d: 26.79 ± 22.32, NR (basline); 20.84 ± 11.90, NR (wave 2); 22.21 ± 15.74, NR (wave 3) | NR |
| Liao et al. [206] | 117 | 73% | 39.8 (NR) | Adults  (MOBILE) | MVPA, LPA; 15-/30 min; bidirectional | NR | MVPA min/15–30 min prior prompt: 0.36–0.72 ± 0.43–0.85, NR; LPA min/15–30 min prior prompt: 4.12–6.78 ± 1.35–2.54, NR; MVPA min/15–30 min after prompt: 0.33–0.7 ± 0.41–0.83, NR; LPA min/15–30 min after prompt: 3.49–7.19 ± 1.25–2.36, NR |
| Madden et al. [95] | 21 | 76.2 % | 49 (NR) | Adults (MDD, bipolar, schizophrenia) | MVPA; 30 min; bidirectional | NR | MVPA min/30 min prior prompt: 1.2 ± 3.0, NR; MVPA min/30 min after prompt: 1.0 ± 2.5, NR |
| McLean et al. [207] | 75 | 63% | 31 (NR) | Adults | Metric; 60 min; before | NR | Steps/60 min prior prompt: 532.81 ± 261.63, 162.44–1605.38 |
| Merikangas et al. [18] | 242 | 61.9% | 48 (NR) | Adults (MDD, bipolar) (NIMH) | Metric; 240 min; bidirectional | NR | NR |
| Michalak et al. [208] | 71 | 60.6% | 39.33 (NR) | Adults (MDD) | Metric; 60 min; before | Acceleration (g): 0.09 ± 0.03 (MDD patients); 0.09 ± 0.03 (controls) | NR |
| Pannicke et al. [209] | 37 | 75.7% | 23.5 (19-28) | Adults | LPA, MPA, VPA and SB; 150 min; before | MVPA min/d: 45.05 ± 42.29 | LPA min/150 min: 21.37 ± 19.48, NR; MPA min/150 min: 6.76 ± 11.84, NR; VPA min/150 min: 0.63 ± 3.93, NR; |
| Pinto et al. [210] | 22 | 100% | 51.5 (NR) | Adults (breast cancer survivors) | MVPA; 1440 min; after | MVPA min/week: 30.18 ± 27.41, NR; | NR |
| Poppe et al. [211] | 38 | 34.2% | 63.18 (50-81) | Adults (with type 2 diabetes mellitus) | LPA, MVPA; 1440 min; after | LPA min/d: 35.54 ± 7.18, 21.57–51.91; MVPA min/d: 6.31 ± 2.82, 0.74–13.93 | NR |
| Powell et al. [212] | 29 | 36% | 71.4 (46-85) | Adults (after joint replacement surgery) | Metric; 60-/1440 min; bidirectional | NR | Activity monitor past activity: 11.08 ± 9.31; activity monitor future activity: 12.34 ± 12.39 |
| Reichert et al. [92] | 106 | 62.4% | 23.4 (18-27) | Adults (URGENCY) | Metric; 10 min; after | Exercise min/week: 186.2 ± 137.8, NR | Non-exercise activity in milli-g/min/participant: 36.3 ± 9.8, 14.3–58. 6 |
| Reichert at al. [88] | 106 | 62.4% | 23.4 (18-27) | Adults | Metric; 15/1440 min; before | Exercise min/week: 188.8 ± 138, 20–570 | Non-exercise activity in milli-g/min/participant: 36.3 ± 9.8, 14.3–58. 6 |
| Ruissen et al. [85] | 126 | 48.4% | 27.71 (18-40) | Adults | MVPA; n.a.*; bidirectional | MVPA min/week: 189.11 ± 184.38, 0–1045.50; length of MVPA bouts: 17.91 ± 5.37, 10.00–43.50 | NR |
| Schwerdtfeger et al. [83] | 124 | 51.6% | 31.67 (18-73) | Adults | Metric, MVPA, LPA; 1-30 min; after | NR | Counts/min/1-min-window: 542.18 ± 1285.21, 0–11838; counts/min/5-min-window: 654.02 ± 1236.97, 0–9771.80; Counts/min/15-min-window: 724.96 ± 1239.65, 0–10383.30; counts/min/30-min-window: 771.74 ± 1195.66, 0–9849.80 |
| Shin et al. [213] | 27 | 29.6% | NR (19-44) | Adults | Metrics; 1440 min; after | Steps/d: 9376 ± NR, 5467 – 16997 | NR |
| Smith et al. [131] | 17 | 58.8% | 10.59 (NR) | Children (with overweight/obesity) | LPA, MVPA; 30-/60-/120 min; bidirectional | LPA min/d: 668.84 ± 148.96, 56.0–961.5; MVPA min/d: 14.31 ± 16.73, 0.0–116.0 | MVPA min/60 min prior prompt: 0.25 ± 1.20, 0–14; LPA min/60 min prior prompt: 33 ± 15,81, 0–59; MVPA min/60 min after prompt: 0.14 ± 0.61, 0–8; LPA min/60 min after prompt: 33.03 ± 15.95, 0–60 |
| Smith et al. [214] | 77 | 41.6% | 15.36 (13-17) | Adolescents (with and without overweight) | Metric, MVPA; 60 min; bidirectional | MVPA min/d: 23.38 ± 18.34, NR | MVPA min/60 min prior prompt: 1.42 ± 1.33, NR; MVPA min/60 min after prompt: 1.15 ± 1.33, NR; Activity counts 15s epochs/60 min prior prompt: 20231.65 ± 8504.27, NR; Activity counts 15s epochs/60 min after prompt: 17829.70 ± 8553.89 |
| Stavrakakis et al. [157] | 20 | 70% (each group) | depressed: 36.4 (22-49) nondepressed: 36.7 (24-46) | Adults (with and without MDD) (MOOVD) | Metric; 360 min; bidirectional | EE/day: 233 ± 77, 126-385 (depressed); 258 ± 69, 120-369 (non-depressed) | NR |
| Stevenson et al. [82] | 25 | 56% | 40 (NR) | Adults (alcohol use disorder) | Metric; 60-/1440 min; before | Steps/d: 8183 ± 5560, 0–30279 | Steps/60 min prior prompt: 553 ± 662, 0-5806 |
| Sudeck et al. [215] | 64 | 58.3% | 35.18 (20-63) | Adults | Metric; 15 min; before | NR | Milli-g/min/15 min prior prompt: 76.24 ± 26.14, 18.86 – 171.62 |
| Takano et al. [216] | 41 | 22% | NR | Adults (undergraduate students) | Metric; 15 min; before | NR | Physical activity [log-transformed]: 5.10 ± 1.51, 0-8.32 |
| Vetrovsky et al. [217] | 28 | 75% | 68 (NR) | Adults | Metric, MVPA; 720 min; after | MVPA min/d: 54 ± 38, NR | NR |
| Walsh et al. [218] | 111 | 60.36% | 22.01 (18-27) | Adults (bipolar) | LPA, MPA, VPA; 1440 min; before | LPA %/activity/day: 33.7 ± 7.75, NR; MPA %/activity/day: 40.12 ± 12.06, NR; VPA %/activity/day: 13.4 ± 5.35 | NR |
| Wen et al. [132] | 202 | 51.67% | 9.6 (8-12) | Children  (MATCH) | MVPA; 30-/, 60 min; bidirectional | NR | MVPA min/30 min prior prompt: 1.83 ± 3.93, 0-30; MVPA min/60 min prior prompt: 3.64 ± 7.16, 0-30; MVPA min/30 min after prompt: 1.78 ± 3.88, 0-30; MVPA min/60 min after prompt: 3.46 ± 6.94, 0-60; |
| Wilhelm et al. [136] | 123 | 63% | 71.83 (64-85) | Elders | Metric; 1440 min after | NR | Counts/min: 292.12 ± 151.65, 41-20-1315.60; Steps/min: 9.34 ±4.64, 0.8-35 |
| Williams et al. [219] | 194 | 71% | 40.72 (20-74) | Adults | Metric; 1440 min; after | NR | NR |
| Yang et al. [79] | 185 | Mothers: 100%; Children: 53% | Mothers: 41.03 (NR); children: 9.51 (NR) | Children, adults (MATCH) | MVPA; 45 min; bidirectional | NR | MVPA min/45 min: 2.345 ± 4.125, 1–45 (children); 1.177 ± 3.057; 1–45 (adults); |
| Zenk et al. [78] | 128 | 100% | NR (25-64) | Adults | MVPA; 1440 min; bidirectional; | MVPA min/d: 15.7 ± 11.2, NR; | NR |
| Zhaoyang & Martire [77] | 152 | 58.04% | 65.39 (NR) | Elders (knee osteoarthritis) | MVPA; 1440 min; bidirectional | MVPA h/d: 1.16 ±0.72, NR; | NR |

*Due to its novel statistical approach, this study cannot be reviewed within the data extraction framework which we custom-developed to the methods applied by most of the AA studies in the PB-AWB field. Abbreviations: d = day; EE = energy expenditure; h = hours; HC = healthy controls; LPA = light physical activity; MDD = major depressive disorder; MET = metabolic equivalent of task; min = minutes; MPA = moderate physical activity; MVPA = moderate to vigorous physical activity; NR = not reported; PA = physical activity; SB = sedentary behavior; SD = standard deviation; VPA = vigorous physical activity
